# Engineering Spin States of Isolated Copper Species in a Metal–Organic Framework Improves Urea Electrosynthesis

**DOI:** 10.1007/s40820-023-01127-0

**Published:** 2023-06-21

**Authors:** Yuhang Gao, Jingnan Wang, Yijun Yang, Jian Wang, Chuang Zhang, Xi Wang, Jiannian Yao

**Affiliations:** 1 Key Laboratory of Photochemistry, Beijing National Laboratory for Molecular Sciences, Institute of Chemistry, Chinese Academy of Sciences, Beijing, 100190 People’s Republic of China; 2 University of Chinese Academy of Sciences, Beijing, 100049 People’s Republic of China; 3 Molecular Plus and Collaborative Innovation Center of Chemical Science and Engineering, Tianjin University, Tianjin, 300072 People’s Republic of China; 4 Department of Physics, School of Physical Science and Engineering, Beijing Jiaotong University, Beijing, 100044 People’s Republic of China; 5 Research Center for Magnetic and Spintronic Materials National Institute for Materials Science, Tsukuba, 305-0047 Japan

**Keywords:** Electrocatalysis, Urea synthesis, Metal–organic framework, Spin catalysis, C–N coupling

## Abstract

The catalytic activities are generally believed to be relevant to the electronic states of their active center, but understanding this relationship is usually difficult. Here, we design two types of catalysts for electrocatalytic urea via a coordination strategy in a metal–organic frameworks: Cu^III^-HHTP and Cu^II^-HHTP. Cu^III^-HHTP exhibits an improved urea production rate of 7.78 mmol h^−1^ g^−1^ and an enhanced Faradaic efficiency of 23.09% at − 0.6 V vs. reversible hydrogen electrode, in sharp contrast to Cu^II^-HHTP. Isolated Cu^III^ species with *S* = 0 spin ground state are demonstrated as the active center in Cu^III^-HHTP, different from Cu^II^ with *S* = 1/2 in Cu^II^-HHTP. We further demonstrate that isolated Cu^III^ with an empty ${d}_{{{\text{x}}^{2}\text{-y}}^{2}}^{0}$ orbital in Cu^III^-HHTP experiences a single-electron migration path with a lower energy barrier in the C–N coupling process, while Cu^II^ with a single-spin state (${d}_{{{\text{x}}^{2}\text{-y}}^{2}}^{1}$) in Cu^II^-HHTP undergoes a two-electron migration pathway.

**Supplementary Information:**

The online version contains supplementary material available at 10.1007/s40820-023-01127-0.

## Highlights

- The single-atom Cu species with *S* = 0 spin ground state in Cu^III^-HHTP have been fabricated.
- The Cu^III^-HHTP exhibits remarkable performance with a high urea yield of 7.780 mmol h^−1^ g^−1^ with the corresponding Faradaic efficiency of 23.09% at − 0.6 V (vs. RHE).
- Low spin state and empty (${d}_{{{\text{x}}^{2}\text{-y}}^{2}}^{0}$) orbitals are favorable to enhance the production urea of C–N coupling process.

**Supplementary Information:**

The online version contains supplementary material available at 10.1007/s40820-023-01127-0.

## Graphical abstract

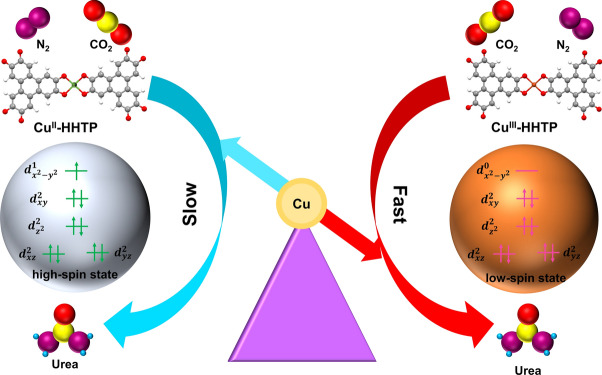

## Introduction

As an important raw material for the chemical industry, urea is generally synthesized by reacting NH_3_ and CO_2_ under harsh conditions (> 400 °C, > 100 bar). The electrocatalysis route toward urea synthesis seems to be another better choice, which obviously is a moderate reaction (N_2_ + CO_2_ + 6H^+^  + 6e^−^ → CO(NH_2_)_2_ + H_2_O) [1–3]. The essential issue in the electrocatalytic urea field remains the design of efficient catalysts. Recently, some attractive progress has been made in this area [4–7]. For example, Yu and co-workers employed the In(OH)_3_-S electrocatalyst for direct and selective urea synthesis from nitrate and carbon dioxide at ambient conditions [8]. In addition, Wang et al. fabricated a new class of oxygen vacancy-enriched CeO_2_ catalyst with the stabilization of the crucial intermediate of *NO via inserting into vacant sites, which was conducive to the subsequent C–N coupling process rather than protonation [9]. Moreover, Zhang’s group reported a novel Mott–Schottky Bi-BiVO_4_ hetero-structures with a remarkable urea yield rate [10]. Furthermore, Ozin and co-workers realized a novel direct urea synthesis from NH_3_ and CO_2_ over new Pd/LTA-3A catalysts powered solely by solar energy, in which Pd nanoparticles served the dual functions for urea formation and LTA-3A removed by-product H_2_O to shift the equilibrium toward urea production [11].

It is well-known that the catalytic activities of heterogeneous catalysts are generally believed to be relevant to the electronic states (including the spin and orbital of electrons) of the active center, but designing efficient catalysts and then understanding the relationship is usually difficult due to the catalysts’ complexity [12–18]. Metal–organic frameworks (MOFs)-based isolated catalysts as unambiguously models make it viable owing to the well-defined feature [19–26]. However, it is still a big challenge to design suitable MOFs-based isolated electrocatalysts for simultaneous fixation of N_2_ and CO_2_ into urea, aiming to investigate the relationship between spin states of single-atom active species and electrocatalytic performance [27–35].

To address the issue “whether the spin states of isolated active species in a catalyst affect the electrocatalytic urea performance?”, herein, we reported on two kinds of MOFs-based single-atom catalysts through a feasible coordination reaction method: Cu^III^-HHTP and Cu^II^-HHTP. When tested in electrocatalytic urea production, Cu^III^-HHTP catalyst exhibited an enhanced urea production rate of 7.78 mmol h^−1^ g^−1^ and an improved Faradaic efficiency of 23.09% at − 0.6 V vs. reversible hydrogen electrode (RHE), which much outperformed Cu^II^-HHTP. We demonstrated that isolated Cu^III^ species with *S* = 0 spin ground state served as the active center in Cu^III^-HHTP, which differed from the isolated Cu^II^ one with *S* = 1/2 spin ground state in Cu^II^-HHTP. Based on in situ Attenuated Total Reflectance Fourier transform Infrared (ATR-FTIR), X-ray emission spectroscopy (XES), operando XAFS measurements, in situ Raman characterization and the density-functional theory (DFT) calculations, we showed that spin states of single-atom copper site played the key role in producing urea. For Cu^III^-HHTP, single-atom Cu^III^ active site possibly experienced a single-electron migration path with a lower energy barrier to facilitate C–N coupling, namely, the σ orbital electron of N_2_ leaped into the empty Cu-*3d* electron orbital (${d}_{{{\text{x}}^{2}\text{-y}}^{2}}^{0}$). For Cu^II^-HHTP, isolated Cu^II^ species possibly underwent a two-electrons migration path with a higher barrier during C–N coupling, that is, the σ orbital electron of N_2_ leaped into the Cu-*3d* orbital electron (${d}_{{{\text{x}}^{2}\text{-y}}^{2}}^{1}$) and then migrated into the empty N_2_-π* orbital.

## Experimental and Calculation

### Materials

2,3,6,7,10,11-Hexahydroxytripheny-lene (HHTP, 97%), Potassium hydrogen carbonate (KHCO_3_, ≥ 99.7%, Alfa), Thiosemicarbazide (CH_5_N_3_S, 99%), Diacetylmonoxime(C_4_H_7_NO_2_, AR), phosphoric acid (H_3_PO_4_, ≥ 85%), sulfuric acid (H_2_SO_4_, ≥ 85%), iron chloride (FeCl_3_, 99.9%), Cuprouschloride (98%), Cupric acetate anhydrous (Cu_2_(OAc)_4_, 99%), N,N-Dimethylformamide (DMF, ≥ 99%) were purchased from Alfa Aesar China Co., Ltd. All reagents were used without further purification.

### Synthesis of Cu^II^-HHTP and Cu^III^-HHTP

The 0.04 mmol Cu_2_(OAc)_4_ and 0.02 mmol HHTP powder were dispersed in 10 mL of deionized water in a 20 mL glass vial, which was sonicated at 60 Hz for 15 min. Adding 1.5 mL DMF to the mixed solution and heating at 60 °C for 12 h to obtain blue Cu^II^-HHTP powder [36]. The resulting products were centrifuged and washed three times in water, ethanol and DMF, alternately. The collected solids were dried at room temperature and saved for further use. Cu^III^-HHTP was slightly modified according to the previous literature report [37]. The preparation of Cu^III^-HHTP was similar to Cu^II^-HHTP catalyst, except for the reaction temperature of 80 °C and in H_2_O_2_. Then, the Cu^III^-HHTP was washed with ethanol and deionized water to remove any impurities and dried in a vacuum oven at 60 °C overnight.

### Characterizations

#### Materials Characterization

Scanning electron microscope (SEM) was performed on a JSM-7800F Prime electron microscope. A transmission electron microscope (TEM) was operated by a JEM-2100F working at 200 kV. X-ray absorption spectra (XAS) measurements were collected at the beamline 1W1B of the Beijing Synchrotron Radiation Facility (BSRF, Beijing). X-ray photoelectron spectroscopy (XPS) was carried out on an ESCALAB250Xi using an Al Kα X-rays as the excitation source. Raman spectra of as-made materials were measured by using HORIBA France, LABRAM HR Evolution. In situ Raman spectroscopy analysis was carried out on a confocal Raman spectrometer with a wavelength of 532 nm. Nitrogen temperature programmed desorption (N_2_-TPD) and carbon dioxide temperature programmed desorption (CO_2_-TPD) were carried out on the AutoChem II 2920. X-ray diffraction pattern (XRD) was conducted by PANanalytical X’PERT PRO MPD diffractometer (Cu Kα radiation = 0.15418 Å, scanned range of 3–90°) to identify the crystalline structure of the as-prepared MOFs. Fourier Transform Infrared Spectrometer (FT-IR) and the spin state of the catalysts were used by VERTEX 70v Bruker spectrometer and MPMS-3 (Quantum Design), respectively. ^1^H NMR spectra were carried out in Bruker AVANCE III HD 700 MHz spectrometer.

#### Electrochemical Measurements

The electrochemical measurements were collected on a three-electrode configuration workstation CHI 660E (Chenhua, Shanghai), where the reference and counter electrodes were Ag/AgCl and graphite rod, correspondingly. The H-type cell was divided by Nafion 211 membrane and used as the electrolyzer for CO_2_ and N_2_ electrochemical reduction reaction in 0.1 M KHCO_3_ solution. Electrocatalytic synthesis of urea measurements is carried out by constant potential electrolysis with a potential window ranging from − 0.3 to − 0.7V (vs. RHE) [1]. Based on the Nernst equation: E (vs. RHE) = E (vs. Ag/AgCl) + 0.0591 × pH + 0.197 (pH = 6.8 in CO_2_-saturated electrolyte and N_2_ + CO_2_-saturated electrolyte in 0.1 M KHCO_3_; pH = 8.3 for N_2_-saturated electrolyte in 0.1 M KHCO_3_). All the potentials were recorded relative to the reversible hydrogen electrode (RHE). The high purity N_2_ (99.999% purity) and CO_2_ (99.99% purity) inlet gases contain traces of impurities such as nitrogen oxides which need to be pre-cleaned by a saturator filled with 0.05 M NaOH and a saturator filled with a 0.05 M H_2_SO_4_ solution to remove the impurities. The electrosynthesis of urea is carried out in a carbon dioxide and nitrogen saturated 0.1 M KHCO_3_ solution for 2 h by electrolysis at room temperature and atmospheric pressure. The results of the double-layer capacitance were used to assess the electrochemically active surface area (ECSA) of the samples, measured in the potential range of − 0.3 ~ − 0.7V against RHE.

##### ***Analysis and Calculation of Urea and NH***_***3***_*** Yield Rate***

The urea products were acquired and analyzed by UV–Vis spectrophotometers of Thermo Fisher (GENSYS 150), which was automated for high-throughput options and room-light resistance. The liquid products of electrolysis were measured by Bruker AVANCE III HD 700 MHz spectrometer. The liquid product was quantified with a linear regression standard equation. The Faradaic efficiency was defined as the amount of electric charge devoted to the synthesis of urea divided by the total charge passed through the electrodes during the electrolysis. Based on the chemical equation (N_2_ + CO_2_ + 6H +  + 6e^–^ → CON_2_H_4_ + H_2_O), six electrons are needed to produce one urea molecule [5]. Thus, the FE can be calculated using Eq. 1:

1 $$\begin{array}{*{20}c} {{\text{FE}} = \frac{{Q_{{{\text{urea}}}} }}{Q} = \frac{{6 \times F \times V \times C_{{{\text{urea}}}} }}{{M_{{{\text{urea}}}} \times Q}}} \\ \end{array}$$

The rate of formation of urea is calculated using Eq. 2:

2 $$\begin{array}{*{20}c} {{\text{Urea}}\;{\text{yield}}\;{\text{rate}} = \frac{{C_{{{\text{urea}}}} \times V}}{{M_{{{\text{cat}}}} \times T \times M_{{{\text{urea}}}} }}} \\ \end{array}$$

where FE is the Faradaic efficiency of the urea product; *Q* is the total number of the charge consumed during the reaction and *Q*_urea_ is the charge corresponding to reduction products; *F* is Faraday constant (96,485 C mol^−1^); *V* is the volume of the cathodic reaction electrolyte in the H-type electrolyzer; *C*_urea_ represents the measurement of the mass concentration of urea in the solution after electrolysis using a UV spectrophotometer; *M*_urea_ is the mass of the catalyst loaded on the carbon cloth. *T* is the time at which the reduction potential is applied [1].

Analysis and calculation of NH_3_ yield rate: The Faradaic efficiency was defined as the amount of electric charge devoted to the synthesis of NH_3_ divided by the total charge passed through the electrodes during the electrolysis [38, 39]. Based on the chemical equation (N_2_ + 6H^+^  + 6e^–^ → 2NH_3_), three electrons are needed to produce one urea molecule. Thus, the FE can be calculated using Eq. 3:

3 $$\begin{array}{*{20}c} {{\text{FE}} = \frac{{Q_{{{\text{NH3}}}} }}{Q} = \frac{{3 \times 0.318 \times F \times V \times C_{{{\text{NH4Cl}}}} }}{{M_{{{\text{NH3}}}} \times Q}}} \\ \end{array}$$

The rate of urea formation was calculated using Eq. 4:

4 $$\begin{array}{*{20}c} {{\text{NH}}_{{3}} \; {\text{yield rate}} = \frac{{0.318 \times C_{{{\text{NH4Cl}}}} \times V}}{{M_{{{\text{cat}}}} \times T \times M_{{{\text{NH4Cl}}}} }}} \\ \end{array}$$

where FE is the Faradaic efficiency of the urea product; *Q* is the total number of the charge consumed during the reaction and Q_NH3_ is the charge corresponding to reduction products; *F* is Faraday constant (96,485 C mol^−1^); *V* is the volume of the cathodic reaction electrolyte in the H-type electrolyzer; ${\text{C}}_{\text{NH4Cl}}$ represents the measurement of the mass concentration of urea in the solution after electrolysis using a UV spectrophotometer; ${\text{M}}_{\text{NH4Cl}}$ is the mass of the catalyst loaded on the carbon cloth. T is the time at which the reduction potential is applied.

#### XAFS Measurements

The X-ray absorption finds structure spectra (Cu K-edge) were collected at 1W1B station in Beijing Synchrotron Radiation Facility (BSRF). The storage rings of BSRF were operated at 2.5 GeV with a maximum current of 250 mA. Using Si (111) double-crystal monochromator, the data collection was carried out in transmission mode using ionization chamber. All spectra were collected in ambient conditions. Operando XAFS measurements: The working electrode was prepared and mounted onto a homemade in situ XAFS fluorescence cell with a three-electrode configuration, where Ag/AgCl (saturated with potassium chloride) and graphite rod were served as the reference and counter electrodes, respectively [40]. During operando XAFS measurements, CO_2_ and N_2_ were continuously bubbled with a flow rate of 20 mL min^−1^ to saturate the 0.1 M KHCO_3_ solution. All operando XAFS spectra were collected in fluorescence mode at room temperature. The linear combination fitting (LCF) was conducted to calculate the content of Cu^2+^ and Cu^3+^ in these treated samples.

#### XES Measurements

The operando Cu Kβ XES measurements were conducted at the 4W1B beamline in Beijing Synchrotron Radiation Facility (BSRF). In a homemade single-chamber XES apparatus, Ag/AgCl (with saturated KCl solution) and graphite rod were selected as reference and counter electrodes, respectively. The Cu Kβ XES spectra were recorded at operating potentials in a decreasing sequence from 0.00, − 0.30, − 0.40, − 0.50 to − 0.70 V vs. RHE, respectively.

#### In situ ATR-FTIR Measurements

The experimental catalyst was obtained by adding 4 mg of catalyst and 20 µL of Nafion (5%) solution to 2 mL of deionized water and sonicating for 1 h. Three-electrode electrochemical cell of a single chamber contains a working electrode, a graphite rod as counter electrode, and a saturated silver chloride electrode as a reference electrode. Electric current was applied to a homemade infrared reflector cell using a CHI 660E electrochemical workstation (Chenhua). All ATR-FTIR spectral acquisitions were carried out after a constant potential was applied to the electrode for 30 min. In situ ATR-IR spectra were measured on a Thermo-Fisher Nicolet II spectrometer, equipped with an MCT cryogenic detector. The measurements were all obtained by 64 scans at a spectral resolution of 4 cm^−1^. The chronoamperometric tests from− 0.3 to− 0.7 *V*_RHE_ were carried out on a CHI 660E electrochemical workstation accompanied by the spectrum collection.

### Computational Details

All of the calculations were performed by means of spin-polarized density-functional theory (DFT) methods using the Vienna Ab initio Simulation Package (VASP) [41]. The exchange–correlation energy was described using the revised Perdew–Burke–Ernzerhof exchange–correlation density functional (PBE) within the generalized-gradient approximation (GGA) [42]. Description of electron–ion interactions by the projector-enhanced wave (PAW) method. The plane-wave cutoff energy was set to 400 eV. The convergence criteria of energy and force were set to 10^–4^ and 0.03 eV Å^−1^, respectively. A vacuum layer of 15 Å was adopted to avoid the interaction between the periodic slabs.

## Results and Discussion

### Characterization of Cu^II^-HHTP and Cu^III^-HHTP

**Fig. 1:**
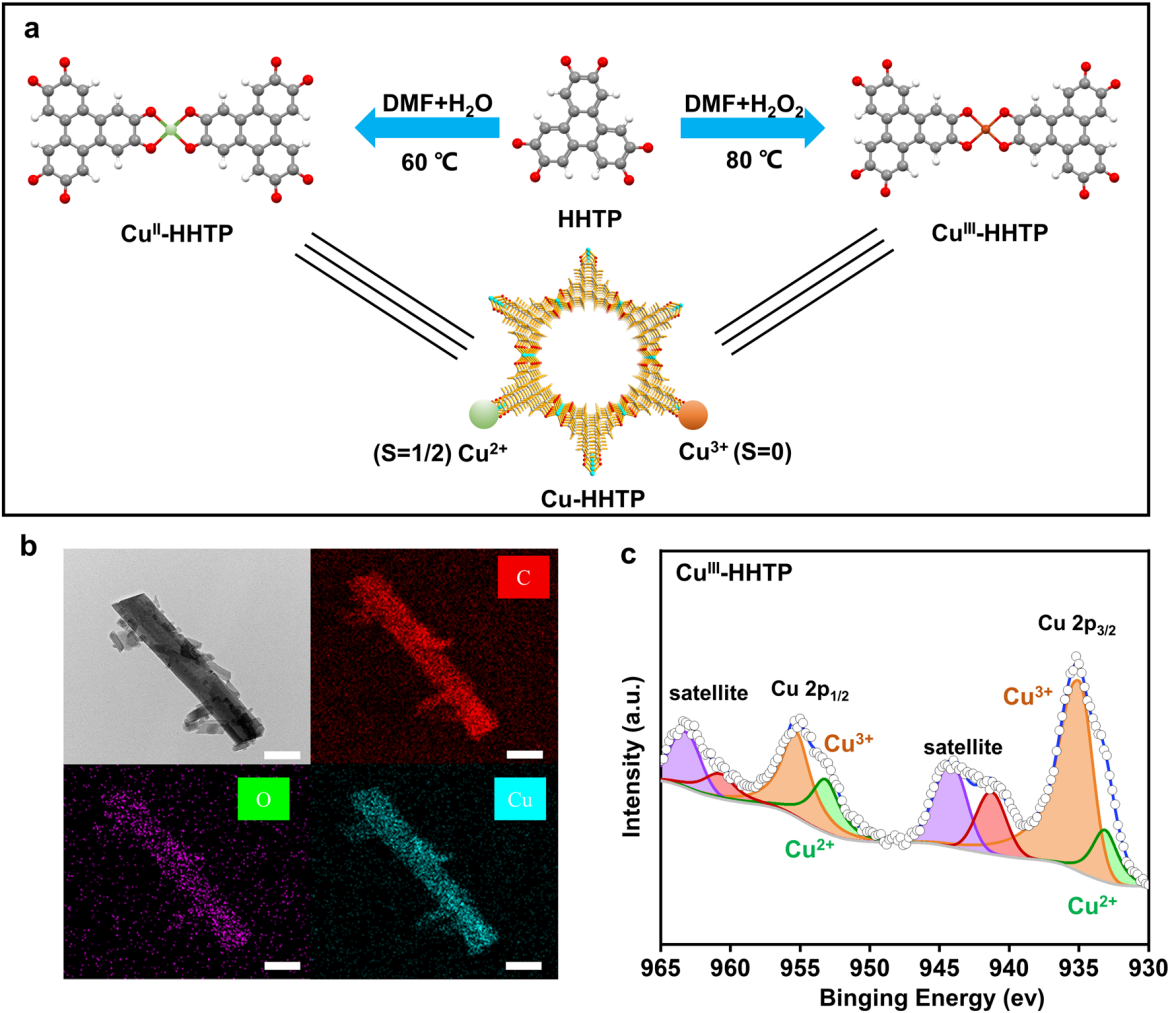
**a** Schematic diagram of the synthesis route. **b** TEM and EDS mapping images of Cu^III^-HHTP. Scale bar: 200 nm. **c** Cu 2*p* spectrum of Cu^III^-HHTP

Both Cu^III^-HHTP and Cu^II^-HHTP catalysts were synthesized via a coordination reaction of 2,3,6,7,10,11-hexahydroxytri-phenylene (HHTP) with Cu^2+^ ions (Fig. 1a), the former was fabricated with the help of the oxidizing agent (H_2_O_2_). Field emission scanning electron microscopy (FESEM) and transmission electron microscopy (TEM) were used to observe their rod-like morphologies (Figs. S1-S2). As illustrated in the energy-dispersive X-ray spectroscopy (EDS) mapping of Cu^III^-HHTP (Fig. 1b), there was a homogeneous distribution of Cu, C, and O elements in it, indicating successful introduction of Cu into a MOF of HHTP. Another evidence from the Fourier transform infrared (FTIR) spectra of two catalysts (Fig. S3), the peak centered at 1211 cm^−1^ was attributed to Cu–O bond, which confirmed formation of Cu–O species in them. The valence states of Cu species in Cu^III^-HHTP and Cu^II^-HHTP catalysts were analyzed by X-ray photoelectron spectroscopy (XPS). As shown in Cu 2*p* XPS spectra of Cu^III^-HHTP (Fig. 1c), the Cu 2*p*_3/2_ and Cu 2*p*_1/2_ signals with the binding energy of 935.5/955.1 eV could be ascribed to Cu^3+^, suggesting the formation of Cu^3+^ species in it. Similarly, the peaks of Cu 2*p*_3/2_ and Cu 2*p*_1/2_ (Fig. S4) were observed at 932.0/934.6 and 951.8/954.5 eV, respectively, demonstrating that the presence of Cu^2+^ species in Cu^II^-HHTP. Nearly the same XRD pattern of two catalysts indicated that Cu^III^-HHTP retained the same crystalline structure as Cu^II^-HHTP (Fig. S5). Raman spectra of two catalysts (Fig. S6) also demonstrated the little differences between them due to the similar *I*_*D*_/*I*_*G*_ ratio.

**Fig. 2:**
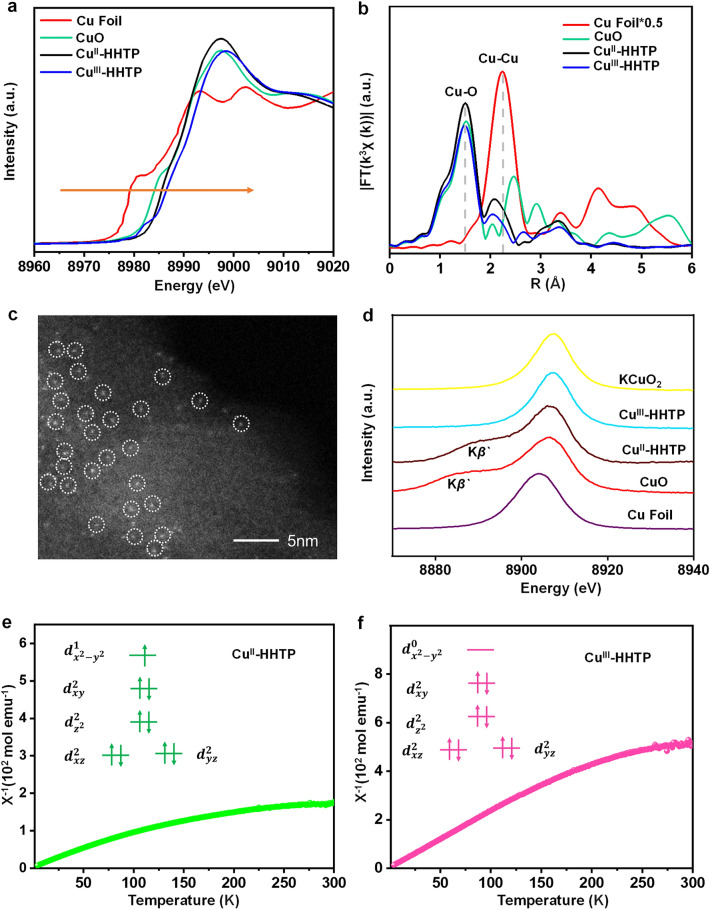
**a** Cu K-edge XANES spectra and **b** Fourier transform EXAFS spectra of Cu-based samples. **c** HAADF-STEM image of Cu^III^-HHTP. **d** Cu Kβ XES of different samples and standard reference materials. Temperature-dependent inverse susceptibility 1/χ **e** Cu^II^-HHTP and **f** Cu^III^-HHTP

To find out the difference in catalytic performance between Cu^III^-HHTP and Cu^II^-HHTP, two catalysts were characterized in detail. The X-ray absorption near-edge structure (XANES) and Extended X-ray absorption fine structure (EXAFS) spectroscopy were conducted to study their chemical states and coordination environment of the Cu center [17, 18]. As shown in Fig. 2a, Cu K-edge XANES spectra of Cu^III^-HHTP show a slight shift to higher energy compared with Cu^II^-HHTP, suggesting a rise of the Cu valence state in Cu^III^-HHTP catalyst. Importantly, The Cu^III^-HHTP pre-edge peak is more obvious than the Cu^II^-HHTP, due to Cu^III^ species with more empty *d* orbitals allowing for an increased electronic transition. As depicted in Fig. 2b, a sole Cu–O peak centered at 1.50 Å can be observed, while no Cu–Cu peak located at 2.34 Å (corrected distance) can be discerned, indicating the isolated Cu-atom structure of Cu^III^-HHTP and Cu^II^-HHTP. As shown in Figs. 2c and S7, the well-defined single-atom structures were then confirmed by the aberration-corrected high-angle annular darkfield scanning TEM (AC HAADF-STEM). The spin states of single-atom Cu species and electronic configuration of the Cu-3*d* orbitals in two catalysts were measured by X-ray emission spectroscopy (XES) and the temperature variable magnetic susceptibility. XES is a powerful tool to confirm the spin states, in which the number of unpaired *3d* electrons can be analyzed by the K*β'* satellite peak due to 3*p-*3*d* exchange coupling [27]. As depicted in Fig. 2d, Cu^III^-HHTP had an insignificant K*β'* intensity splitting similar to the reference KCuO_2_, implying that the Cu-3*d* orbitals of Cu^III^-HHTP did not hold the unpaired electron with the possible electron configuration (${d}_{{{\text{x}}^{2}\text{-y}}^{2}}^{0}$). In contrast, Cu^II^-HHTP showed distinct K*β'*, indicating that its Cu-3*d* orbitals contained unpaired electrons with the possible electronic configuration (${d}_{{{\text{x}}^{2}\text{-y}}^{2}}^{1}$) as compared with the reference CuO. The peak position and intensity of Cu^III^-HHTP are almost identical to KCuO_2_, which may imply that the two Cu species are in a similar valence state and both in the Cu-O_4_ coordination mode. In addition, the comparison of the characteristic peaks of Cu K*β*_1,3_ with the reference samples again suggested that the average oxidation state of single-atom Cu species was + 3 for Cu^III^-HHTP, whereas the value of single-atom Cu species was + 2 for Cu^II^-HHTP. From temperature-dependent susceptibility curves (Fig. 2e, f), it can be seen that the isolated Cu species embedded in Cu^III^-HHTP and Cu^II^-HHTP were Cu^III^ with S = 0 spin ground state and Cu^II^ with 1/2 spin ground state, respectively [14]. In addition, *μ*_eff_ values of Cu^II^-HHTP/Cu^III^-HHTP were calculated of 4.47/2.04, which were related to the number of unpaired electrons in them (Fig. S8, Table S1). Therefore, the 3*d* electron configuration of single-atom Cu center in two catalysts are illustrated in the insets shown in Fig. 2e, f. That is, there was an empty Cu-3*d* electron orbital (${d}_{{{\text{x}}^{2}\text{-y}}^{2}}^{0}$) in Cu^III^-HHTP and the single-spin state (${d}_{{{\text{x}}^{2}\text{-y}}^{2}}^{1}$) in Cu^II^-HHTP.

### Electrocatalytic Performance

**Fig. 3:**
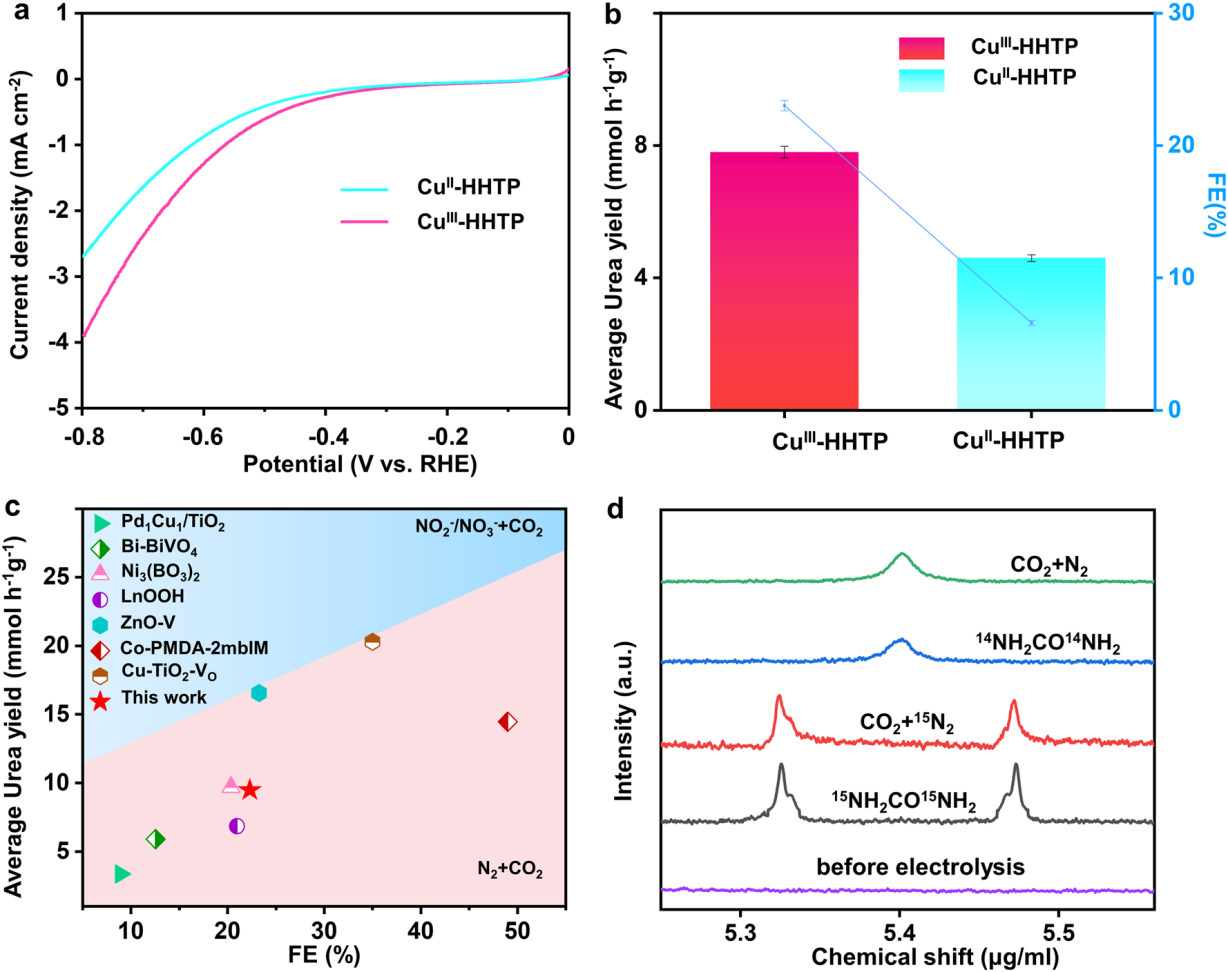
**a** The linear sweep voltammetry (LSV) of Cu^III^-HHTP and Cu^II^-HHTP catalysts in CO_2_ + N_2_ saturated electrolyte. **b** The urea yield rate and Faradaic efficiencies of Cu^III^-HHTP and Cu^II^-HHTP at− 0.6 V versus RHE during a testing period of 2 h. **c** Comparison of Cu^III^-HHTP with the established electrocatalysts at their maximum reported urea yield. **d** ^1^H NMR spectra of electrolyte saturated with ^15^N_2_ + CO_2_ / ^14^N_2_ + CO_2_ after 2 h electrolysis, standard ^15^NH_2_CO^15^NH_2_ / ^14^NH_2_CO^14^NH_2_ solution and before electrolysis

An H-shaped electrochemical cell with a three-electrode configuration was used to measure the urea performance of above two catalysts (Fig. S9). In situ ATR-FTIR tests were performed under working conditions to understand the catalytic process (Fig. S10). Herein, an independent evaluation approach consisting of the diacetyl monoxime and indophenol blue tests were utilized (Figs. S11-S12), where the urea, NH_3_ and other gas by-products (e.g., H_2_ and CO) were analyzed by the UV–vis spectrophotometry and online gas chromatography. And, the possible liquid by-products, including N_2_H_4_, NO_2_^−^, and NO_3_^−^, were quantified by ultraviolet–visible (UV–Vis) spectrophotometry (Figs. S13-S15) [43]. More importantly, the potential by-product of N_2_H_4_, NO_2_^−^ and NO_3_^−^ was not detected in the solution that had been reacted for 2 h in the N_2_ + CO_2_ saturated 0.1 M KHCO_3_ electrolyte at each potential (Figs. S16-S18). As depicted in the typical linear sweep voltammogram (LSV) (Fig. S19), Cu^III^-HHTP catalyst in a CO_2_ + N_2_ saturated electrolyte (pink curve) showed much stronger current density compared with the cases in CO_2_ or N_2_ saturated electrolytes, demonstrating urea electrocatalysis happened in it. It is noted that Cu^III^-HHTP catalyst obviously exhibited superior electrocatalytic activity than Cu^II^-HHTP (Fig. 3a), since it had higher current density over the same potential range. Therefore, Cu^III^-HHTP delivered an ultrahigh urea yield of 7.78 mmol h^−1^ g_cat_^−1^ and corresponding improved Faradaic efficiency (FE) of 23.09% (Figs. 3b and S20) at the optimal potential of − 0.6 V vs. reversible hydrogen electrode (RHE). As can be seen from Fig. 3c and Table S2, this urea yield value was 2 times higher than that of Cu^II^-HHTP and surpassed the reported hitherto electrocatalysts for urea synthesis in the N_2_ + CO_2_ testing system. However, the higher potential inhibited urea synthesis (Fig. S21) possibly due to the excessive CO release and enhanced competing hydrogen evolution reactions (HER). More importantly, Cu^III^-HHTP exhibited good catalytic stability. The time-dependent current density curves recorded for 2 h at various potentials illustrate its superior durability (Fig. S22), and there was almost negligible decay in the current density at − 0.6 V (vs. RHE) after 18 h chronoamperometry test (Fig. S23). Notably, the urea yield and FE kept well for five consecutive cycles (Fig. S24). Moreover, the electrochemical impedance spectra (EIS) of two catalysts also indicated that it might be related to the charge transfer (Fig. S25). Furthermore, we measured and then calculated the electrochemically active surface area (ECSA) of two catalysts (Figs. S26-S27), which was an essential parameter for the evaluation of electrochemical reactivity. It is obvious that Cu^III^-HHTP had larger ECSA than Cu^II^-HHTP (570.0 vs. 337.5 cm^−2^), indicating that the Cu^III^ species were more active than Cu^II^ one. Isotope-labeled nuclear magnetic resonance (NMR) was used to disclose the origin of urea product over Cu^III^-HHTP. As shown in Fig. 3d, the doublet coupling peak of CO(^15^NH_2_)_2_ appeared on the ^1^H NMR spectrum only when ^15^N_2_ and CO_2_ were used as feeding gases, suggesting that the urea production was derived from an electrocatalytic process on Cu^III^-HHTP catalyst rather than the contamination nitrogen species from the ambient environment. It is also noted that the urea concentrations calculated by ^1^H-NMR were essentially identical to the UV/Vis method (Figs. S28-S29). Therefore, it is demonstrated that the detected urea came from the simultaneous catalyzed reaction of N_2_ and CO_2_ over the Cu^III^-HHTP catalyst. This was further testified by a series of control experiments (Fig. S30), including a CO_2_/N_2_ saturated solution at − 0.6 V, a mixed gas (N_2_ + CO_2_)-saturated electrolyte at open circuit potential, CO_2_ + N_2_ saturated solution without applied voltage and before/after 2 h electrolysis at bare carbon cloth.

**Fig. 4:**
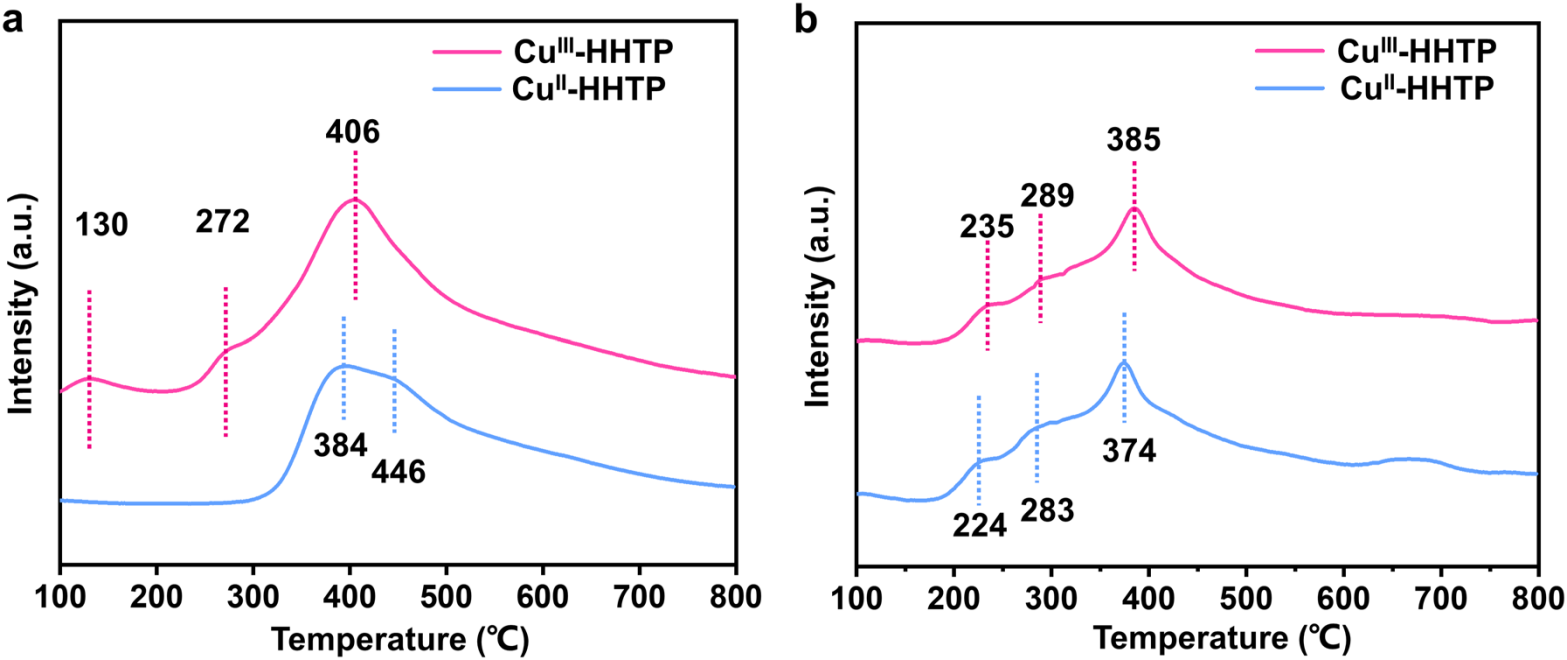
**a** N_2_-TPD spectra of Cu^III^-HHTP and Cu^II^-HHTP catalysts. **b** CO_2_-TPD spectra of Cu^III^-HHTP and Cu^II^-HHTP catalysts

To further study the target adsorption sites of CO_2_ and N_2_ and their coupling effect over two catalysts, the N_2_-temperature programmed desorption (N_2_-TPD) and CO_2_-TPD spectra were measured. As illustrated by the N_2_-TPD spectrum (Fig. 4a), the Cu^III^-HHTP sample exhibits new prominent TPD peaks at 130 and 272 ℃, which can be attributed to the physical adsorption of N_2_ [44]. This indicates that N_2_ binding is stronger on the surface of the Cu^III^-HHTP catalyst than on that of the Cu^II^-HHTP. The N_2_ desorption peak of Cu^III^-HHTP spectrum presents a peak located at 406 °C, while the N_2_ desorption peak shifts to 446 ℃ for Cu^II^-HHTP, and an additional peak at 384 °C appears. The peak shift to a higher temperature and the additional appearance prove that the desorption of N_2_ is more difficult, cumbersome and unfavorable to facilitate the desorption of subsequent electrocatalytic synthesis products. As shown in Fig. 4b, the CO_2_ desorption peaks of Cu^III^-HHTP were at 235, 289 and 385 °C, which were slightly higher than those of Cu^III^-HHTP (224, 283 and 374 °C, respectively). The Cu^III^-HHTP with a low spin state has a higher chemisorption capacity for CO_2_ compared to Cu^II^-HHTP. It demonstrated that the stronger N_2_ and CO_2_ binding strength over Cu^III^-HHTP catalysts might facilitate the subsequent C-N coupling process of urea.

**Fig. 5:**
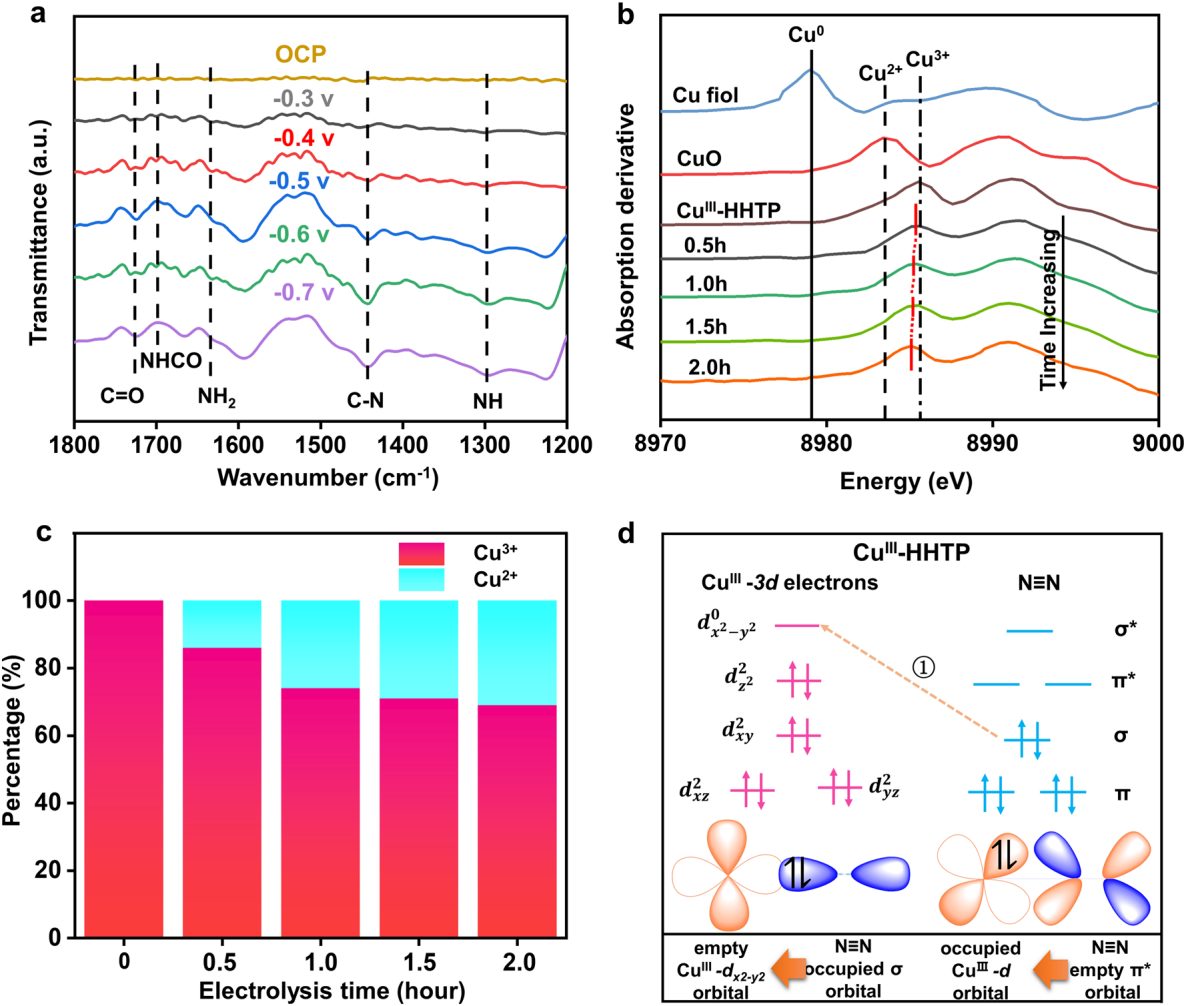
**a** In situ ATR-FTIR spectra with negative scan from− 0.3 to− 0.7 V vs. RHE. **b** Cu K-edge first-order derivatives of the Cu^III^-HHTP recorded over time at − 0.6 V versus RHE. **c** The linear combination (LCF) fitting result of the Cu K-edge XANES spectra recorded over time at − 0.6 V versus RHE. **d** Illustration of the single-electron migration pathway of Cu^III^-HHTP during C–N coupling

To reveal the possible intermediates and reaction paths during urea synthesis, in situ ATR-FTIR spectra were then measured (Fig. 5a). The most remarkable feature in it is that the strongest peak located at 1449 cm^−1^ was attributed to the stretching mode of C–N, which consequently provided strong evidence on urea electrocatalytic synthesis. The simultaneous emergence of other peaks such as -CO and -NH_2_ meant the *NCONH intermediate products formed in the catalysis, which were regarded as the key factor in effective urea production. The infrared band at around 1694 cm^−1^ has been observed to be the consequence of *NHCO species. The generation of NHCO intermediates, representing the intermediate species for N_2_ and CO_2_ reduction, respectively, implying that urea production is associated with the co-adsorption of N_2_ and CO_2_ [4]. Notably, the C–N infrared signal of Cu^III^-HHTP was considerably stronger than Cu^II^-HHTP (Fig. S31), and the intensity maximum was found at -0.6 V vs. RHE, which coincides with the electrocatalytic tests. These meant that the C–N coupling pathways over two catalysts were different and Cu^III^-HHTP was more favorable to C–N coupling. Herein we employed climbing image nudged elastic band (CI-NEB) to calculate the transition states of the C–N bond formation step over two catalysts. As shown in Fig. S32, for Cu^III^-HHTP, a lower barrier of 1.405 eV needed to surmount for the formation of *NCONH*, as compared with 2.398 eV for Cu^II^-HHTP, indicating the more kinetically accessible of Cu^III^-HHTP. It is worth noting that the energy barrier of N_2_ activation was the largest in the whole C–N coupling process, it again demonstrated that it was a rate-determining step in the urea synthesis. The possible electron configuration evolution of single-atom Cu species was investigated by an in situ XES. Figure S33a shows the Cu K*β* XES spectra of Cu^III^-HHTP with the scan range from− 0.3 to− 0.9 V (vs. RHE), which were regularly recorded by electrolysis under saturated solution (CO_2_ + N_2_) for 2 h. It can be found that there was a negative shift in the Cu K*β*_1,3_ emission energy with reaction potential, indicating that the oxidation state (+ 3) of Cu^III^ decreased during C–N coupling. The in situ XES spectra with a slight negative shift (0.1 eV) of the main Kβ_1,3_ line at OCP to− 0.7V vs. RHE. That is, the electrons from the σ orbitals of N_2_ were most likely to migrate into the empty orbitals (${d}_{{{\text{x}}^{2}\text{-y}}^{2}}^{0}$) of single-atom Cu^III^ site. In comparison, there were little changes in the in situ XES for Cu^II^-HHTP under the same conditions (Fig. S33b). These once more suggested that Cu^III^-HHTP indeed preferred to produce urea compared with Cu^II^-HHTP owing to its special spin states, since both catalysts had isolated Cu structure and similar Cu–O coordination numbers. In addition, a slight K*β'* for Cu^III^-HHTP appears during the reaction, demonstrating that spin states of single-atom Cu site might change. Therefore, the Cu oxidation state (+ 2 or + 3) obviously had a dramatical influence on the spin states and in turn decided the Cu-3*d* electron distribution/configuration, leading to the significantly different N_2_ activation pathways on the single-atom Cu active centers over two catalysts. The investigation of the ratio evolution of Cu^III^ /Cu^II^ over electrolysis time during the electrocoupling of N_2_ and CO_2_ under operating conditions is critical to reveal the intrinsic reaction mechanisms [40]. The operando XAFS experiment was used to directly monitor the change in actual catalytic central valence state of Cu^III^-HHTP catalyst. With increased electrolysis time, the empty ${d}_{{{\text{x}}^{2}\text{-y}}^{2}}^{0}$ orbitals of Cu^III^-HHTP with a low spin state are continuously filled with σ electrons from N_2_, which may mean that the valence state of the active center decreases. First-order derivatives absorption of Cu K-edge Cu^III^-HHTP was measured for each spectrum at the corresponding time of electrolysis. As shown in Fig. 5b, the XANES dramatically changed with the electrolysis time indicating that a fall of Cu valence state in Cu^III^-HHTP. It is noted that the linear combination fitting (LCF) of the XANES spectrum of Cu^III^-HHTP, all fitted by similar method (Fig. S34), revealing that they are all composed of Cu^2+^ and Cu^3+^ (Fig. 5c). It can be obviously seen that the accumulation of electrolysis time leads to more σ electrons transfer to the empty ${d}_{{{\text{x}}^{2}\text{-y}}^{2}}^{0}$ orbitals of Cu^III−^HTP, which in turn leads to the changes of the ratio of Cu^3+^ /Cu^2+^. More importantly, 69% of the Cu^3+^ was still present in the Cu^III^-HHTP catalyst at the end of the reaction (Table S3). Furthermore, EXAFS curve-fitting (Table S4) suggested that Cu^II^-HHTP and Cu^III^-HHTP catalysts had the same Cu–O coordination number, which was also consistent with Cu K-edge wavelet transform (WT)-EXAFS (Figs. S35-S36). In addition, a weakening of the Raman intensity of the Cu–O bond was observed at the reduction potential compared to the open circuit voltage, which may be related to the breakage of the Cu–O bond during catalysis and the formation of the Cu-O_x_ intermediate species (Fig. S37) [45]. The reaction pathways of *NCONH* formation over the two catalysts are summarized in Fig. 5d. Note that the single-electron migration path occurred toward N_2_ activation during C–N coupling for Cu^III^-HHTP (*S *= 0), where the σ orbital electron of N_2_ leaped into the empty Cu-3*d* electron orbital (${d}_{{{\text{x}}^{2}\text{-y}}^{2}}^{0}$). In comparison, there were two-electron migration steps in above process for Cu^II^-HHTP (*S* = 1/2), where the σ orbital electron of N_2_ leaped in the Cu-3*d* orbital electron (${d}_{{{\text{x}}^{2}\text{-y}}^{2}}^{1}$) and then transferred into the empty N_2_-π* orbital (Fig. S38). It is obvious that more efficient N_2_ activation way existed in Cu^III^-HHTP (*S* = 0) compared with Cu^II^-HHTP (*S* = 1/2), resulting in improved catalytic activity.

**Fig. 6:**
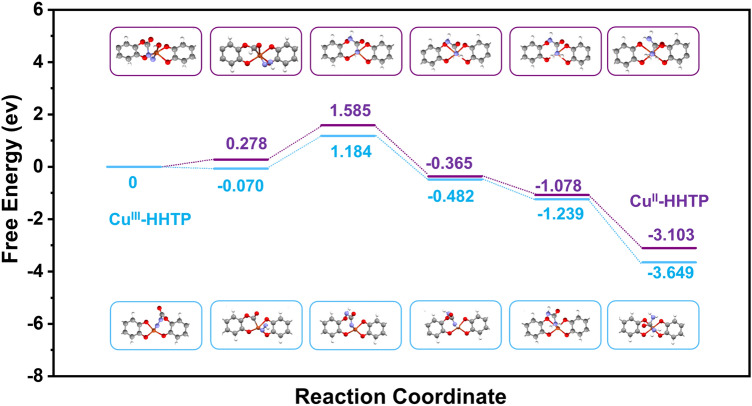
Free energy diagram over Cu^III^-HHTP (blue) and Cu^II^-HHTP (purple) catalysts with the corresponding geometry structures

The possible reaction pathways of Cu^III^-HHTP and Cu^II^-HHTP catalysts were also explored by using DFT calculations (Fig. 6 and Table S5). In order to decode the possible CO_2_ and N_2_ absorption sites over Cu^III^-HHTP, the density-functional theory (DFT) was conducted. The electron density theoretical simulation analysis (Fig. S39) revealed that the isolated Cu^III^ site and the adjacent O site in Cu^III^-HHTP possibly acted as the active centers toward the coupling of N_2_ and CO_2_ due to the electronic interaction. As shown in Fig. S40, their density of states (DOS) demonstrates that the spin states of the two materials are different. The urea reactions over Cu^III^-HHTP initiated with the reaction of N_2_ and CO_2_ and dissociated to form Cu-N_2_ and *CO subsequently. Then, an effective C–N coupling happened. Then, a series of thermodynamically downhill hydrogenation steps occurred for following reactions over two catalysts. The first two hydrogen atoms tent to bond on two nitrogen atoms, the third hydrogen was prone to bonding at the nitrogen in Cu–N owing to the smaller electron delocalization. Followed by the last hydrogenation step, urea molecule was produced. It can be seen that Cu^III^-HHTP was more favorable for the C–N coupling to produce urea than Cu^II^-HHTP. That is, the spin-state transition of single-atom Cu site in Cu^III^-HHTP greatly affected N_2_ activation and thus C–N coupling over Cu^III^ (*S* = 0) site, achieving the much high urea production rate and FE compared with previous reports (Table S2).

## Conclusions

In summary, we reported novel MOFs-based isolated copper catalysts (Cu^III^-HHTP and Cu^II^-HHTP) through a coordination method and aimed to answer the question: whether the spin states of isolated active species in a catalyst affect the electrocatalytic urea performance. As tested in the electrocatalytic urea production, Cu^III^-HHTP catalyst showed an improved urea activity of 7.78 mmol h^−1^ g^−1^ with the corresponding Faradaic efficiency of 23.09% at − 0.6 V (vs. RHE), as compared with Cu^II^-HHTP. Multiple characterizations including in situ ATR-FTIR, XES, operando XAFS measurements, in situ Raman and the theoretical calculations revealed that spin states of isolated copper sites play a key role in producing urea: Cu^III^ active center in Cu^III^-HHTP had the empty Cu-*3d* electron orbital (${d}_{{{\text{x}}^{2}\text{-y}}^{2}}^{0}$)and possibly experienced a single-electron migration path with a lower energy barrier in the C–N coupling process; while the Cu^II^ site in Cu^II^-HHTP had a single-spin state (${d}_{{{\text{x}}^{2}\text{-y}}^{2}}^{1}$) and might undergo a two-electron migration pathway, leading to produce few urea. Therefore, engineering spin states of single-atom active species in catalysts is envisaged to be a new effective approach to improve electrocatalytic activities of urea synthesis.

### Supplementary Information

Below is the link to the electronic supplementary material.

Supplementary file1 (PDF 3295 kb)
