# Supplementary material for: Engineering Spin States of Isolated Copper Species in a Metal–Organic Framework Improves Urea Electrosynthesis
Source: Nanomicro Lett. 2023 Jun 21;15:158. doi: 10.1007/s40820-023-01127-0 (PMC10284786; doi:10.1007/s40820-023-01127-0)
Supplement: Supplementary file 1 — Supplementary file1 (PDF 3295 kb) [file 40820_2023_1127_MOESM1_ESM.pdf]

Supporting Information for

Engineering Spin States of Isolated Copper Species in a Metal−Organic Framework Improves Urea Electrosynthesis

Yuhang Gao[1, 2, †], Jingnan Wang[3, †], Yijun Yang[4, *], Jian Wang[5], Chuang Zhang[1, 2, *], Xi Wang[4, *], and Jiannian Yao[1, 2]

[1] Key Laboratory of Photochemistry, Beijing National Laboratory for Molecular Sciences, Institute of Chemistry, Chinese Academy of Sciences, Beijing 100190, People's Republic of China

[2] University of Chinese Academy of Sciences, Beijing 100049, People's Republic of China

[3] Molecular Plus and Collaborative Innovation Center of Chemical Science and Engineering, Tianjin University, Tianjin 300072, People's Republic of China

[4] Department of Physics, School of Physical Science and Engineering, Beijing Jiaotong University, Beijing, 100044, People's Republic of China

[5] Research Center for Magnetic and Spintronic Materials National Institute for Materials Science Tsukuba 305-0047, Japan

[†]Yuhang Gao and Jingnan Wang contributed equally to this work.

*Corresponding authors. E-mail: zhangchuang@iccas.ac.cn (Chuang Zhang), yijunyang@bjtu.edu.cn (Yijun Yang); xiwang@bjtu.edu.cn (Xi Wang)

Supplementary Figures and Tables

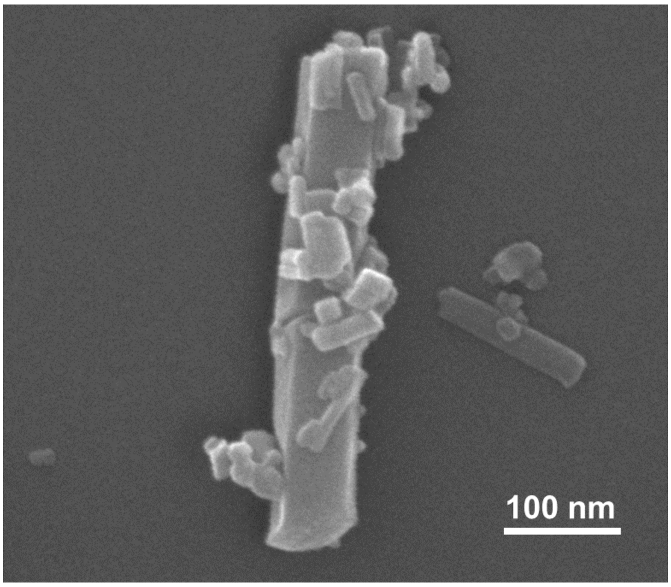

Fig. S1 SEM of image Cu^{III}-HHTP

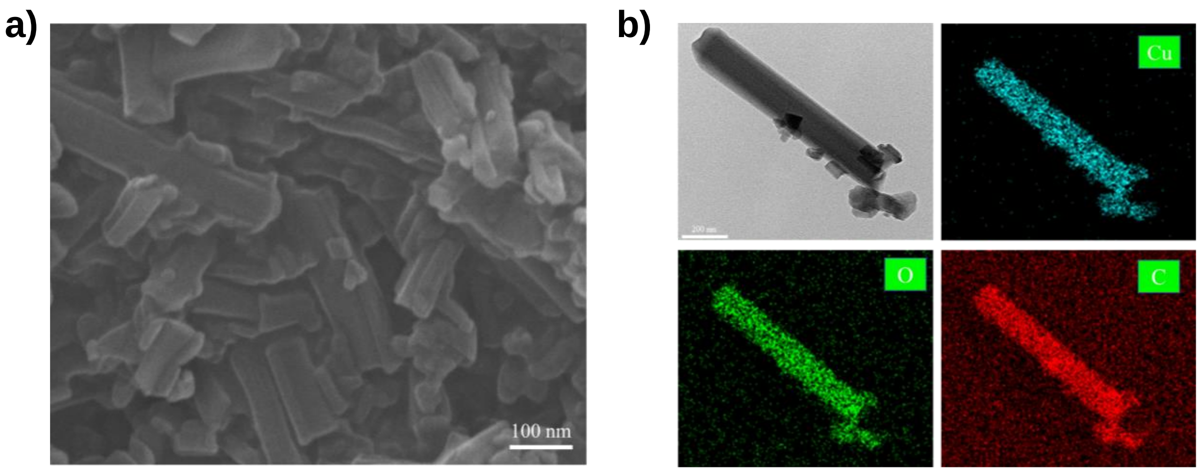

Fig. S2 a SEM and **b** TEM image and EDS elemental mapping of as-made Cu^{II}-HHTP

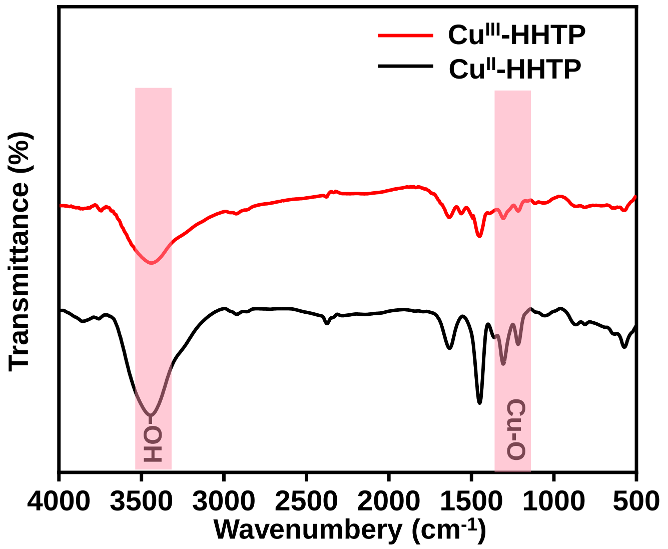

Fig. S3 FT-IR spectra of Cu^{II}-HHTP and Cu^{III}-HHTP

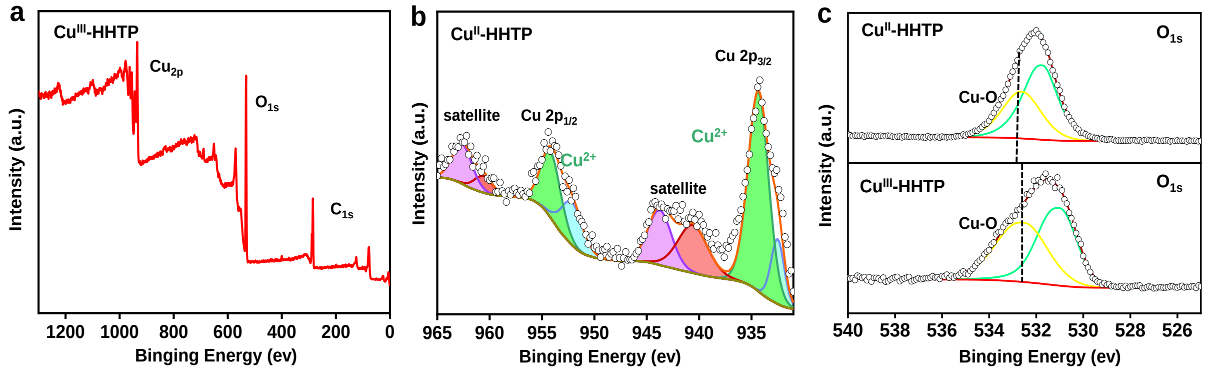

Fig. S4 a XPS survey curve of Cu^{III}-HHTP, **b** Cu 2p spectrum of Cu^{II}-HHTP and **c** O 1s spectra of Cu^{III}-HHTP and Cu^{II}-HHTP

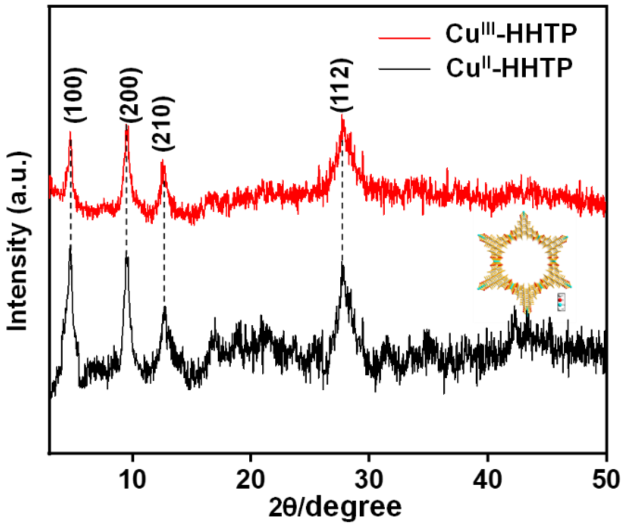

Fig. S5 XRD patterns of two catalysts

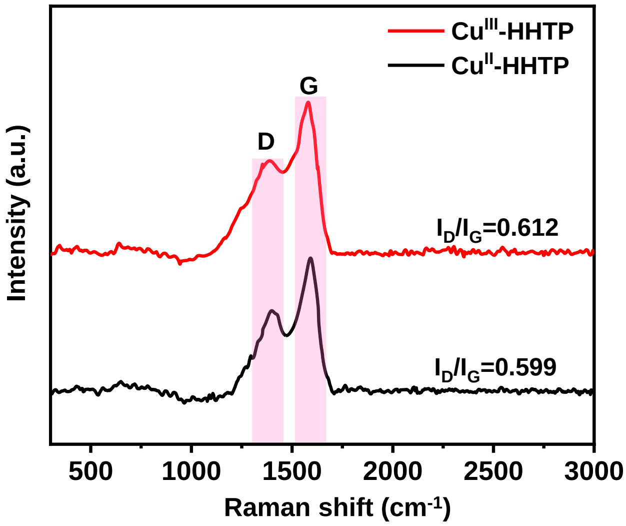

Fig. S6 Raman spectra of two catalysts

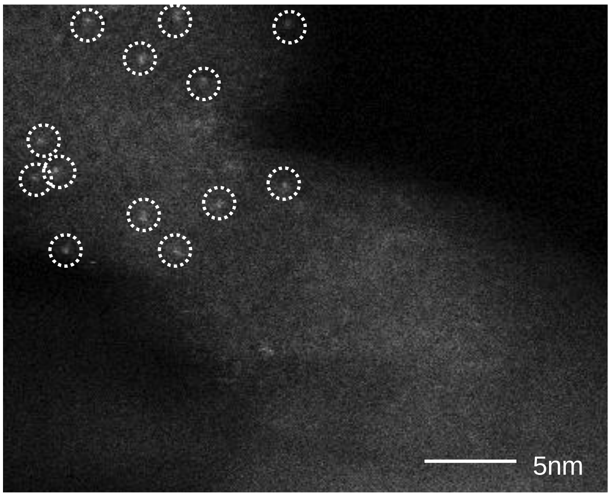

Fig. S7 HAADF-STEM image of Cu^{II}-HHTP

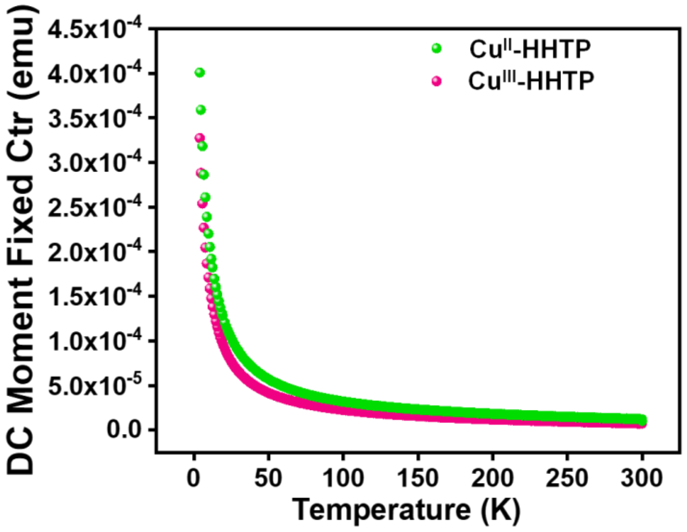

Fig. S8 Temperature-dependent susceptibility χ for Cu^{II}-HHTP and Cu^{III}-HHTP catalysts

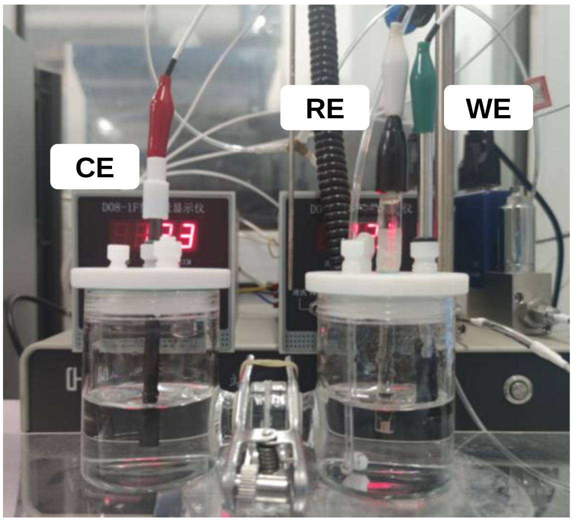

Fig. S9 The optical photograph of the H-type cell and gas purification unit for urea electrosynthesis testing

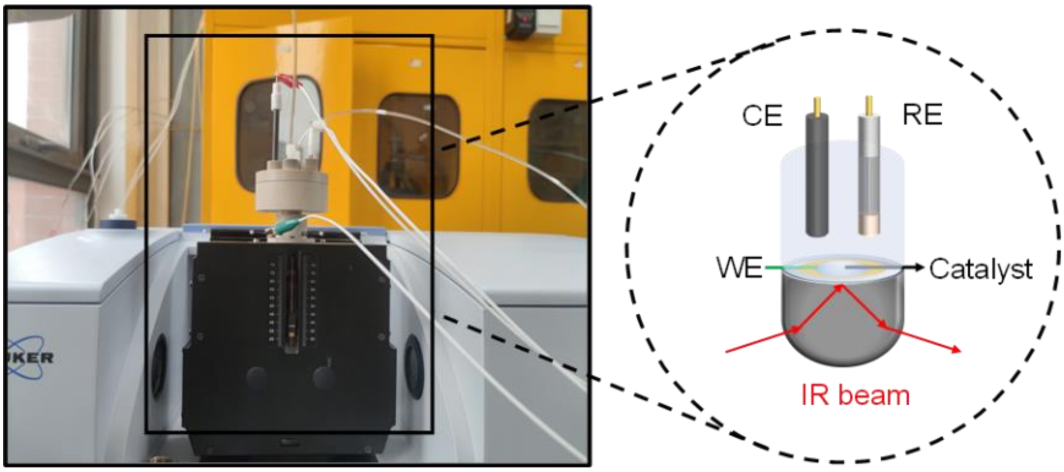

Fig. S10 The schematic diagram of *in situ* ATR-FTIR tests

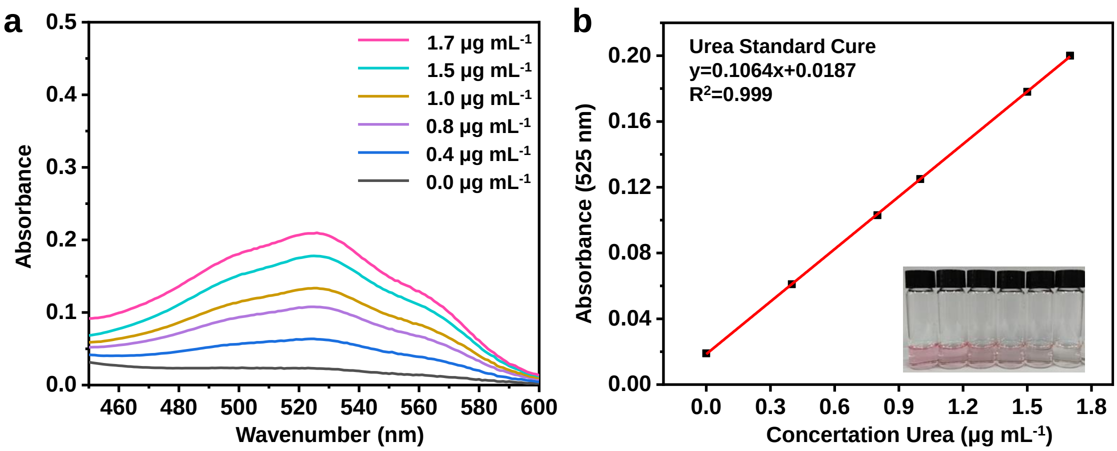

Fig. S11 **a** UV-vis curves and **b** concentration-absorbance of urea solution with a series of standard concentration (0.0-1.7 μg mL^{-1}) in 0.1 M $KHCO_3$ solution. The standard curve shown good linear relation of absorbance with urea concentration (y = 0.1064x + 0.0187, R^2=0.999)

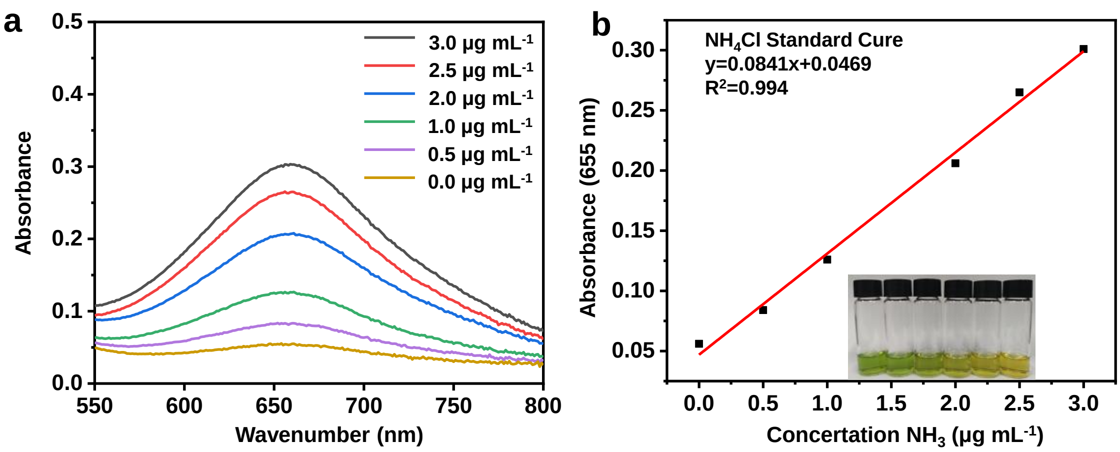

Fig. S12 **a** UV-vis curves and **b** concentration-absorbance of NH_4Cl solution with a series of standard concentration (0.0-3.0 μg mL^{-1}) in 0.1 M $KHCO_3$. b) The standard curve shown good linear relation of absorbance with NH_4Cl concentration (y=0.0841x+0.04694, R^2=0.994)

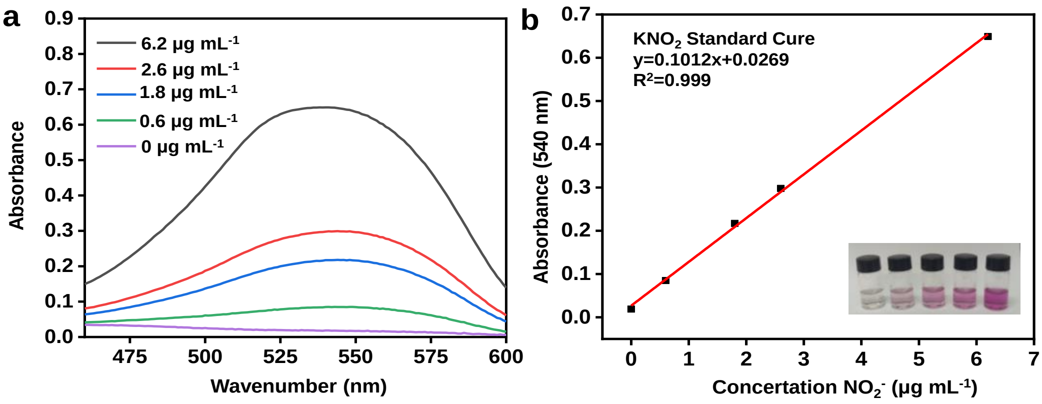

Fig. S13 **a** UV-vis curves and **b** concentration-absorbance of KNO_2 solution with a series of standard concentration (0.0-6.2 μg mL^{-1}) in 0.1 M $KHCO_3$. The standard curve shown good linear relation of absorbance with KNO_2 concentration (y = 0.1012x + 0.0269, R^2=0.999)

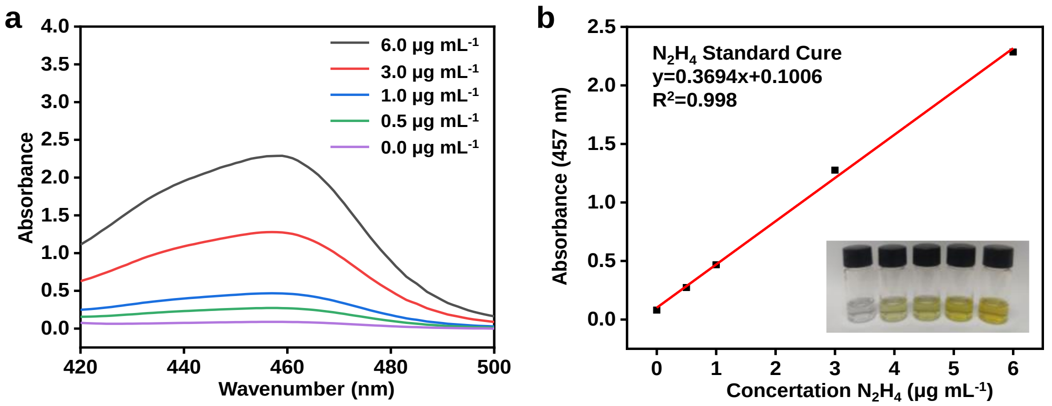

Fig. S14 a UV-vis curves and **b** concentration-absorbance of N_2H_4 solution with a series of standard concentration (0.0-6.0 μg mL^{-1}) in 0.1 M $KHCO_3$. The standard curve shown good linear relation of absorbance with N_2H_4 concentration ($y = 0.3694x + 0.1006$, R^2=0.998)

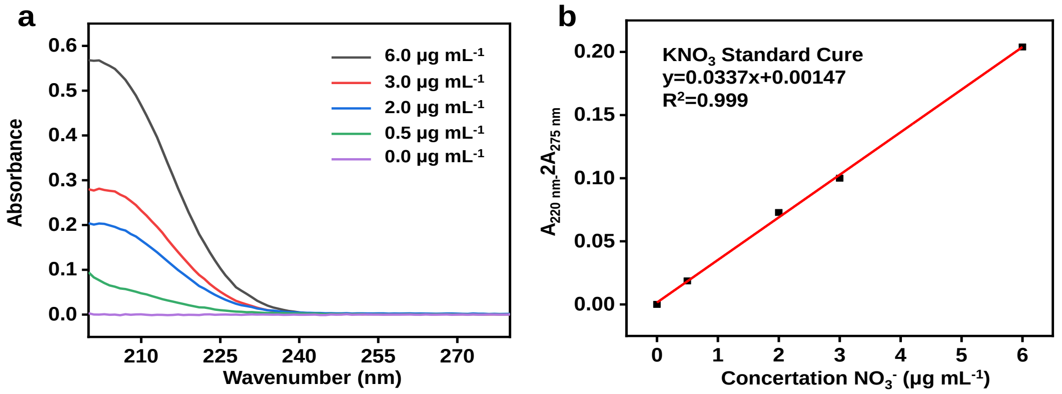

Fig. S15 a UV-vis curves and **b** concentration-absorbance of KNO_3 solution with a series of standard concentration (0.0-6.0 μg mL^{-1}) in 0.1 M $KHCO_3$. The absorbance was measured by UV-vis spectrophotometer. The standard curve shown good linear relation of absorbance with KNO_3 concentration ($y = 0.0337x + 0.00147$, R^2=0.999)

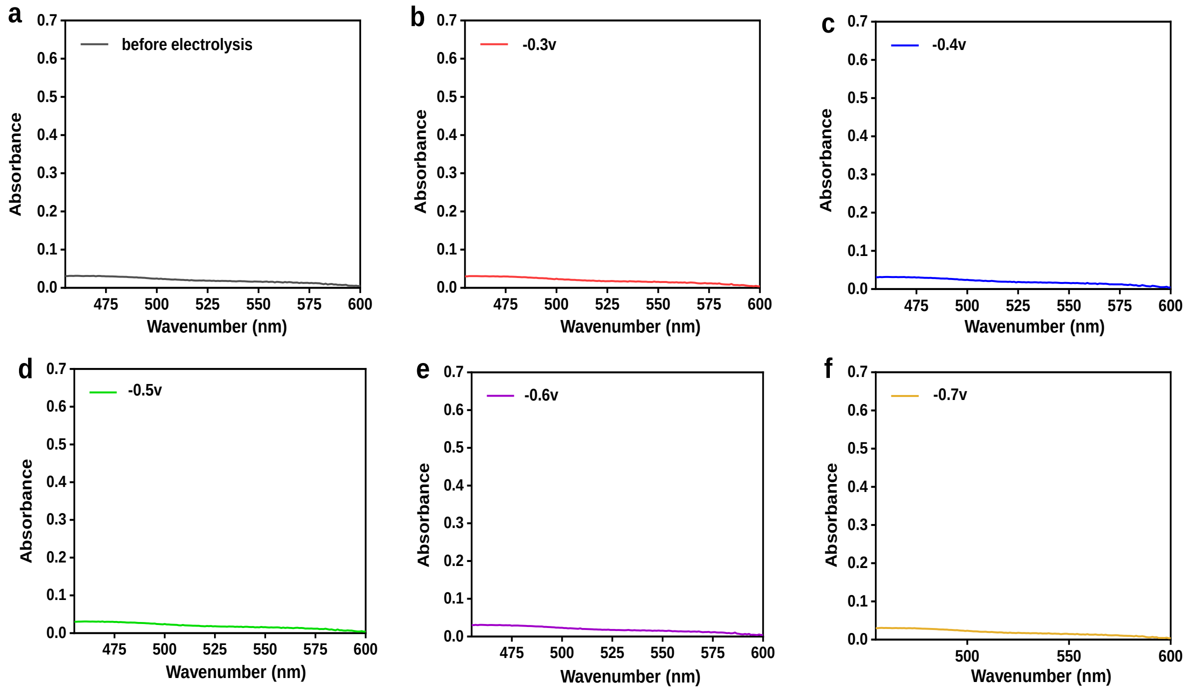

Fig. S16 Absorption spectra of the electrolyte after Cu^{III}-HHTP catalysts at various potentials for 2h in N_2 and CO_2-saturated 0.1 M $KHCO_3$ solution to quantify NO_2^-

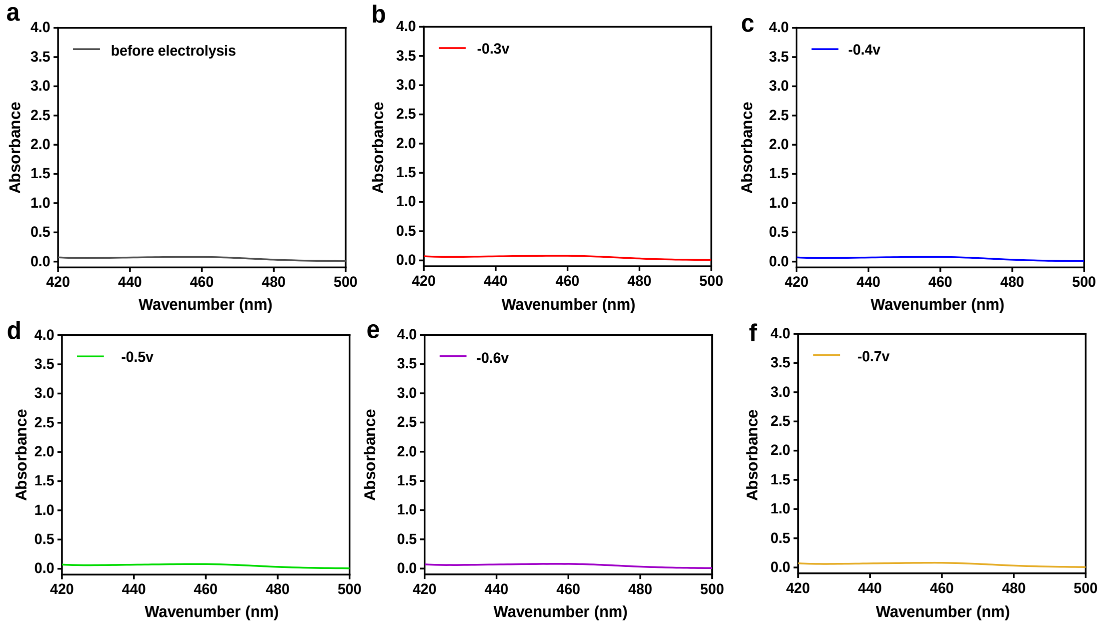

Fig. S17 Absorption spectra of the electrolyte after Cu^{III}-HHTP catalysts at various potentials for 2h in N_2 and CO_2-saturated 0.1 M $KHCO_3$ solution to quantify N_2H_4

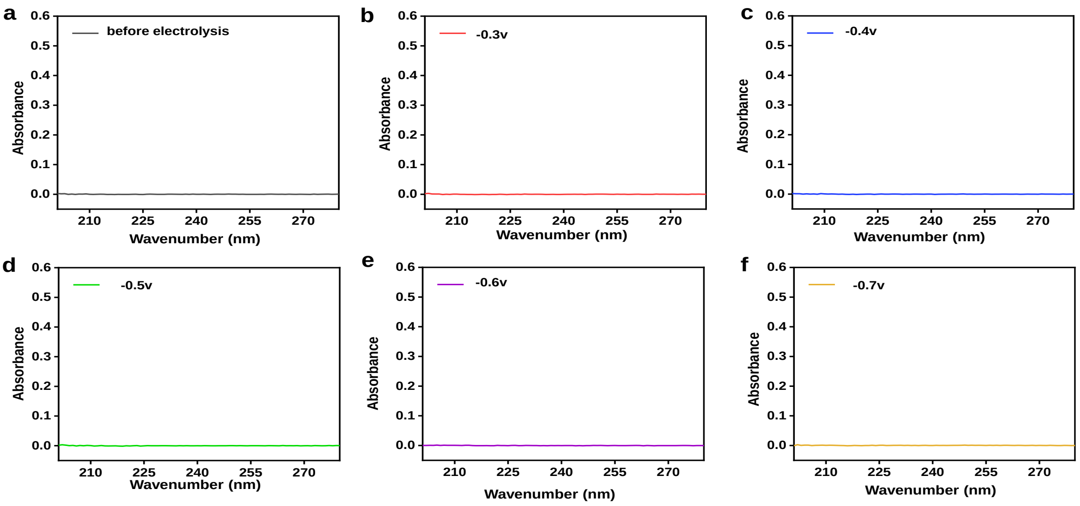

Fig. S18 Absorption spectra of the electrolyte after Cu^{III}-HHTP catalysts at various potentials for 2h in N_2 and CO_2-saturated 0.1 M $KHCO_3$ solution to quantify NO_3^-

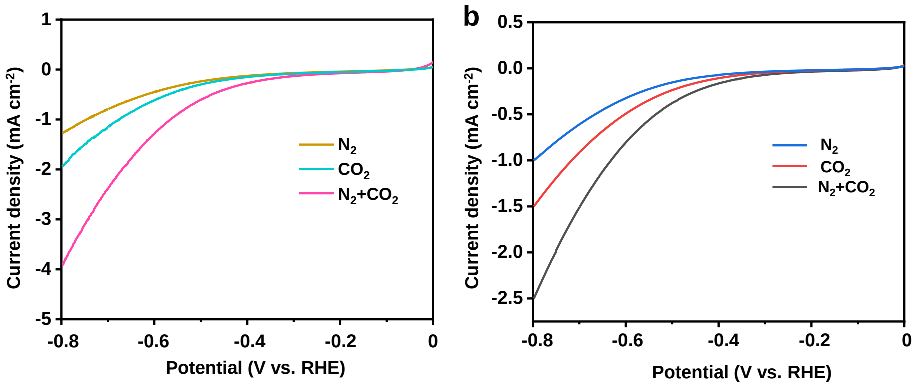

Fig. S19 The linear sweep voltammetry (LSV) of **a** Cu^{III}-HHTP and **b** Cu^{II}-HHTP catalysts in CO_2, N_2 and CO_2 + N_2 saturated electrolyte

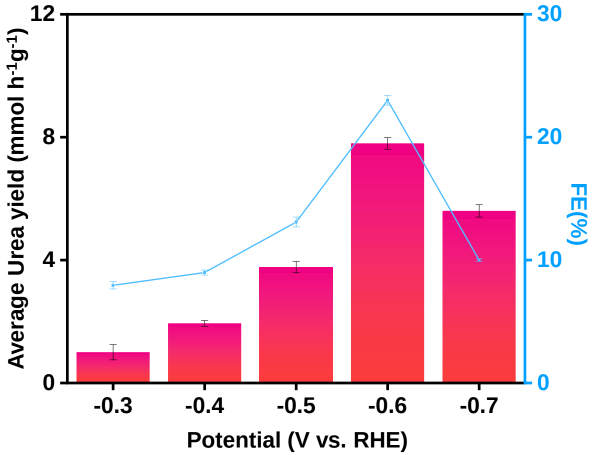

Fig. S20 The urea yield rate and Faradaic efficiencies of Cu^{III}-HHTP catalyst at the different potentials

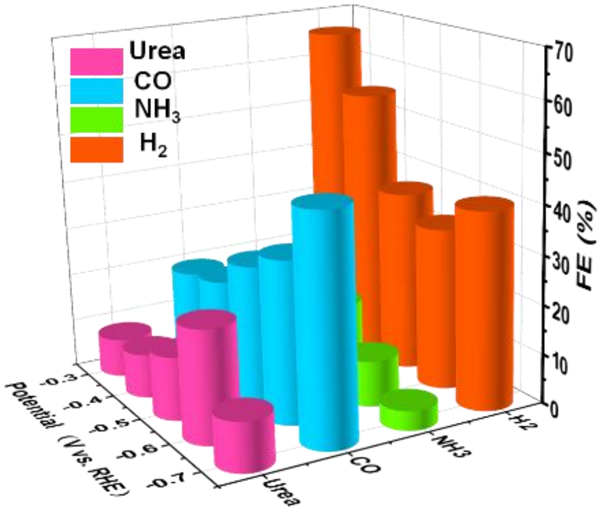

Fig. S21 The urea yield rate, Faradaic efficiencies and the corresponding product distribution of H_2 (orange), CO (blue), NH_3 (green), and urea (pink) with N_2 and CO_2 as the feeding gas at various potentials for Cu^{III}-HHTP catalysts

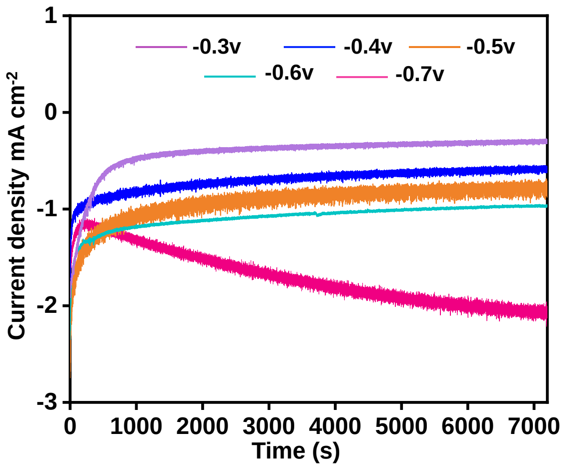

Fig. S22 The chronoamperometric curves Cu^{III}-HHTP catalysts at various potentials for 2h in N_2 and CO_2-saturated 0.1 M $KHCO_3$ solution

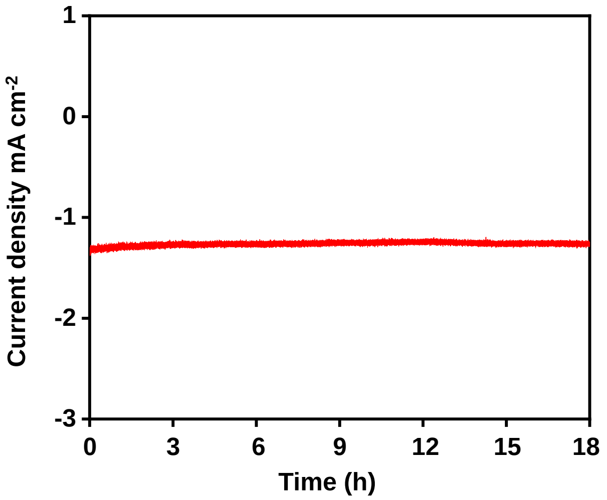

Fig. S23 The chronoamperometric curves of Cu^{III}-HHTP catalyst at -0.6 V vs. RHE for 18 h in N_2 + CO_2-saturated 0.1 M $KHCO_3$ solution

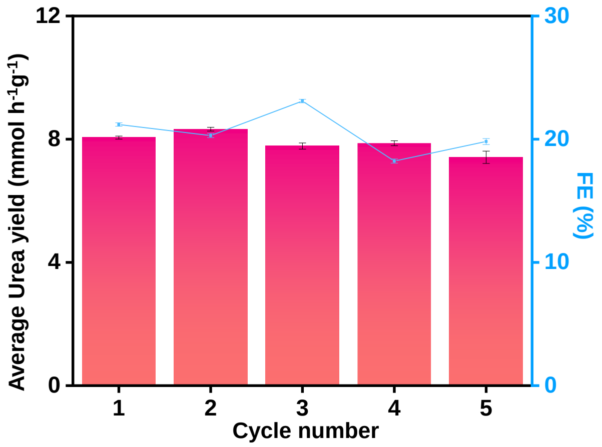

Fig. S24 The Faradaic efficiency and urea production rate of Cu^{III}-HHTP catalysts at -0.6 V vs. RHE during five recycling tests

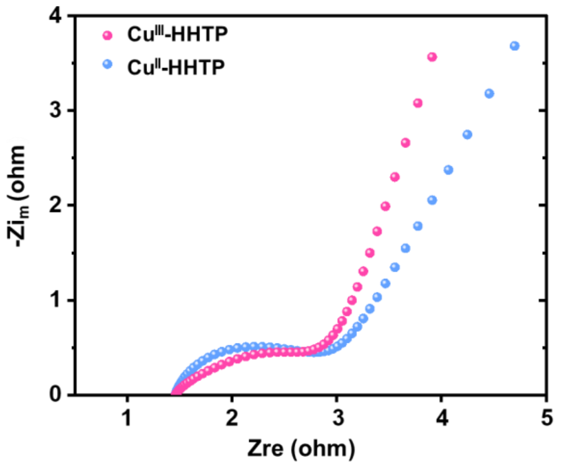

Fig. S25 Nyquist plots of electrochemical impedance spectra (EIS) of Cu^{II}-HHTP and Cu^{III}-HHTP

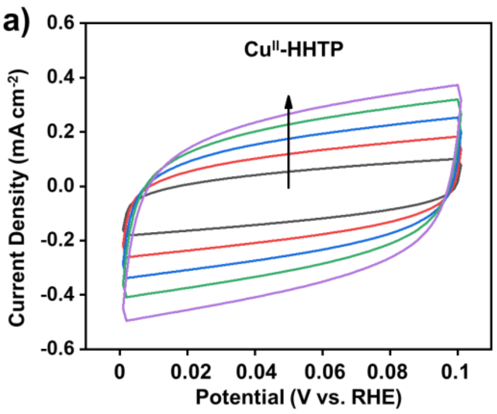

Fig. S26 CV curves of **a** Cu^{II}-HHTP, **b** Cu^{III}-HHTP under different scan rates from 4 to 20 mV s^{-1}

The electrochemically active surface area (ECSA) can be determined through the double layer capacitance (C_{dl}) varying the scan rate of cyclic voltammetry (CV) curves, which is an essential parameter for the evaluation of electrochemical reactivity. The double layer capacitance (Cdl) of the two MOFs samples was determined by the slope of the linear fit of ΔJ = Ja - Jc at 0.05 V (vs. RHE) and the scan rate (**Fig. S26**). The specific capacitance of a generally slick flat surface is between 20 and 60 μF cm^{-2}, thus 40 μF cm^{-2} is used as a reference in this work to calculate the ECSA. The ECSA of Cu^{II}-HHTP, (b) Cu^{III}-HHTP is 337.5 cm^{-2} 570.0 cm^{-2}, respectively.

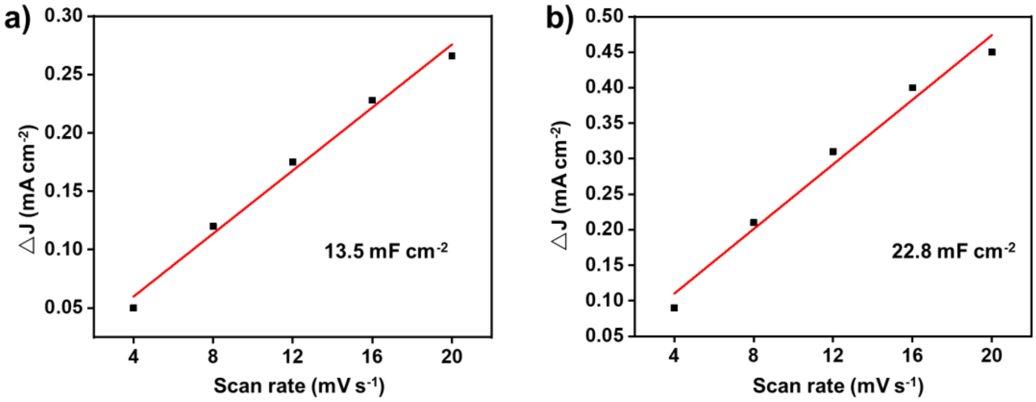

Fig. S27 The fitted linear relationship between current density and the scan rate of (**a**) Cu^{II}-HHTP, **b** Cu^{III}-HHTP

In the calculation of the electrochemically active surface area for the three samples below, we have assumed a specific capacitance of 40 μF cm^{-2} for a flat surface, which is calculated as follows：

$$A_{ECSA}^{Cu^{II}-HHTP} = \frac{13.5\ mF\ cm^{-2}}{40\ \mu F\ cm^{-2}\ per\ cm_{ECSA}^2} = 337.5\ cm_{ECSA}^2$$

$$A_{ECSA}^{Cu^{III}-HHTP} = \frac{22.8\ mF\ cm^{-2}}{40\ \mu F\ cm^{-2}\ per\ cm_{ECSA}^2} = 570.0\ cm_{ECSA}^2$$

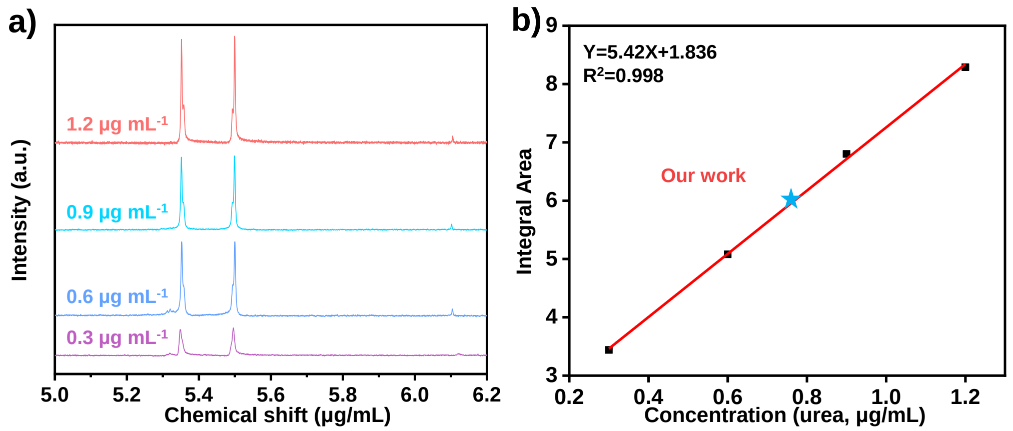

Fig. S28 **a** The calibration curves for $^{15}NH_2CO^{15}NH_2$ solution at concentrations of 0.3-1.2 μg/mL, **b** The corresponding calibration curve for $^{15}NH_2CO^{15}NH_2$ solution

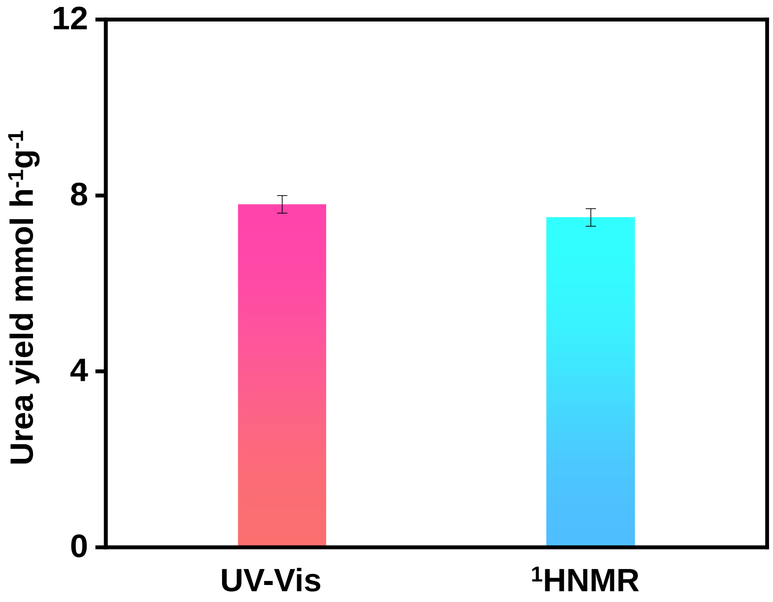

Fig. S29 The urea yield of Cu^{III}-HHTP catalyst after 2 h electrolysis detected by UV/Vis and 1H NMR spectroscopy

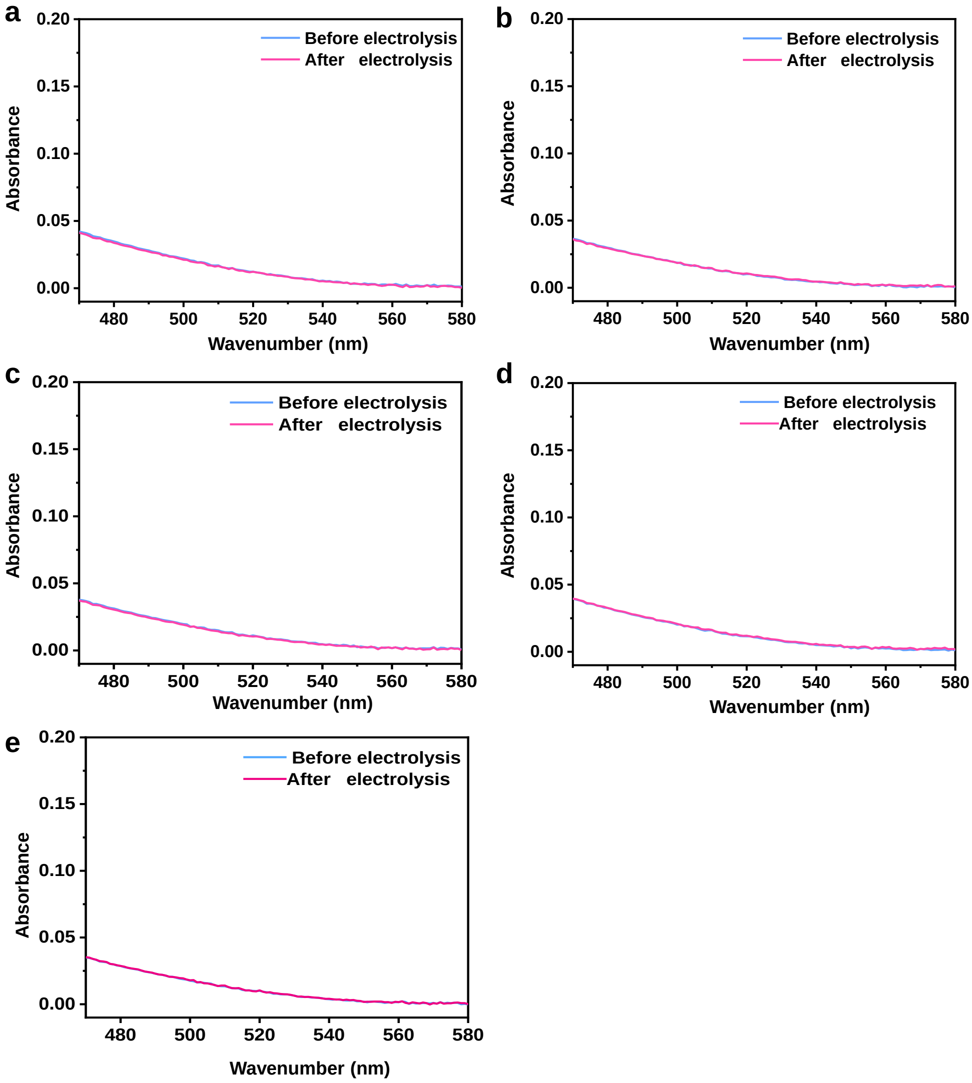

Fig. S30 a UV-Vis absorption spectra of the 0.1 M $KHCO_3$ electrolyte stained with urea color agent before and after 2 h electrolysis at -0.6 V in CO_2 saturated electrolyte, **b** UV-Vis absorption spectra of the 0.1 M $KHCO_3$ electrolyte stained with urea color agent before and after 2 h electrolysis at -0.6 V in N_2 saturated electrolyte, **c** UV-Vis absorption spectra of the 0.1 M $KHCO_3$ electrolyte stained with urea color agent before and after continuously supplying N_2 and CO_2 for 2 h without applied voltage, **d** UV-Vis absorption spectra of the 0.1 M $KHCO_3$ electrolyte stained with urea color agent before and after 2 h electrolysis at open-circuit potential under ambient conditions, **e** UV-Vis absorption spectra of the 0.1 M $KHCO_3$ electrolyte stained with urea color agent before and after 2 h electrolysis at bare carbon cloth

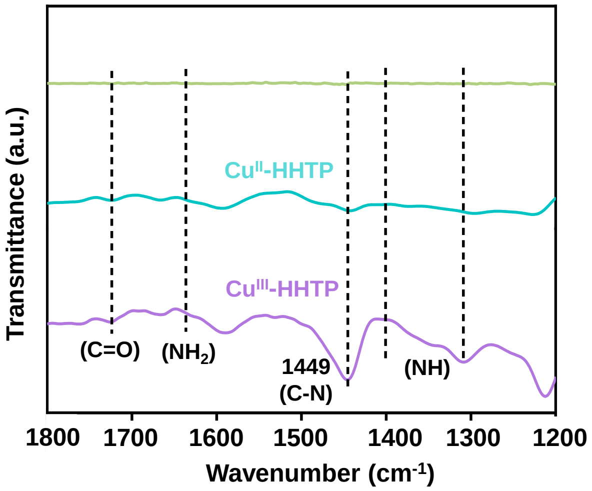

Fig. S31 Under -0.6 V versus RHE for Cu^{II}-HHTP and Cu^{III}-HHTP during the electroreduction of N_2 and CO_2 processes

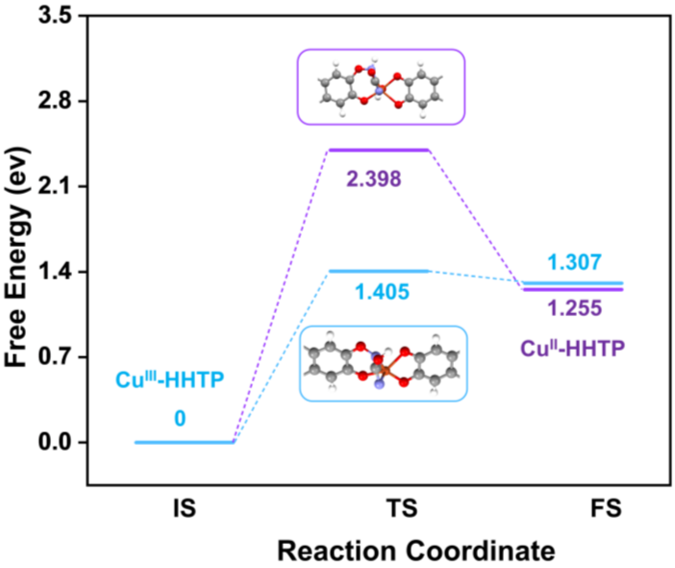

Fig. S32 The reaction pathway of *NCONH* formation over Cu^{III}-HHTP. The structures of the initial (IS), transition (TS) and final (FS) states

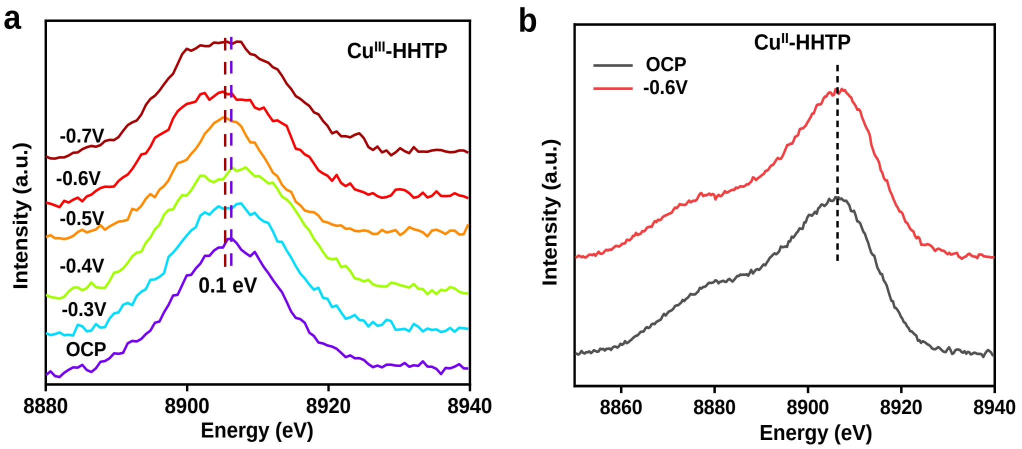

Fig. S33 In situ Cu Kβ X-ray emission spectroscopy (XES) of **a** Cu^{III}-HHTP and **b** Cu^{II}-HHTP under different potentials

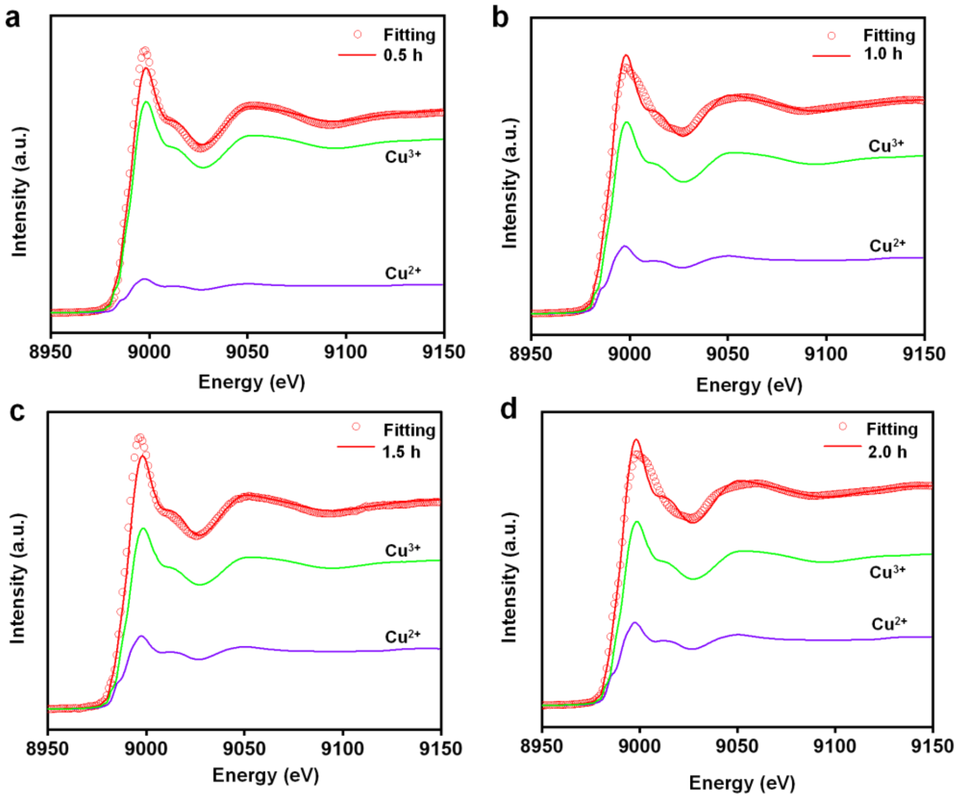

Fig. S34 The linear combination fitting (LCF) result of the Cu K-edge XANES spectra during -0.6V vs. RHE. **a** 0.5 h, **b** 1 h, **c** 1.5 h and **d** 2 h

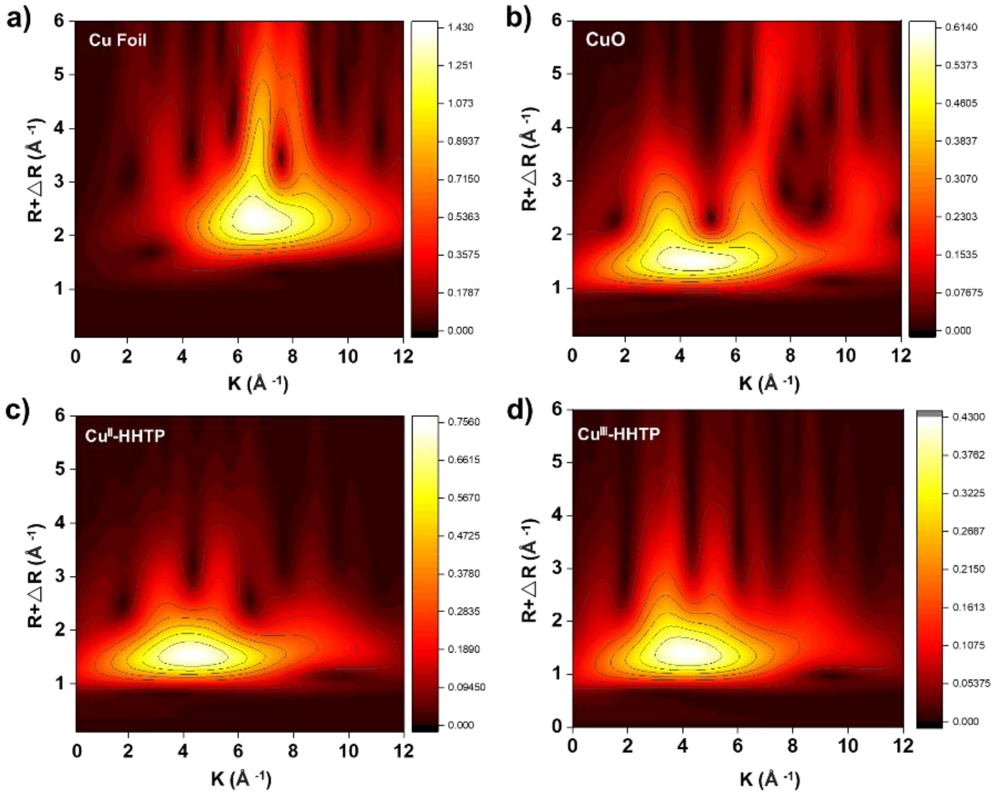

Fig. S35 The EXAFS WT signatures of **a** Cu foil, **b** CuO, **c** Cu^{II}-HHTP, and **d** Cu^{III}-HHTP.

For Wavelet Transform analysis, the χ(k) exported from Athena was imported into the Hama Fortran code. The parameters were listed as follow: R range, 1-6 Å, k range, 0-12 $Å^{-1}$; k weight, 2; and Morlet function with κ=10, σ=1 was used as the mother wavelet to provide the overall distribution

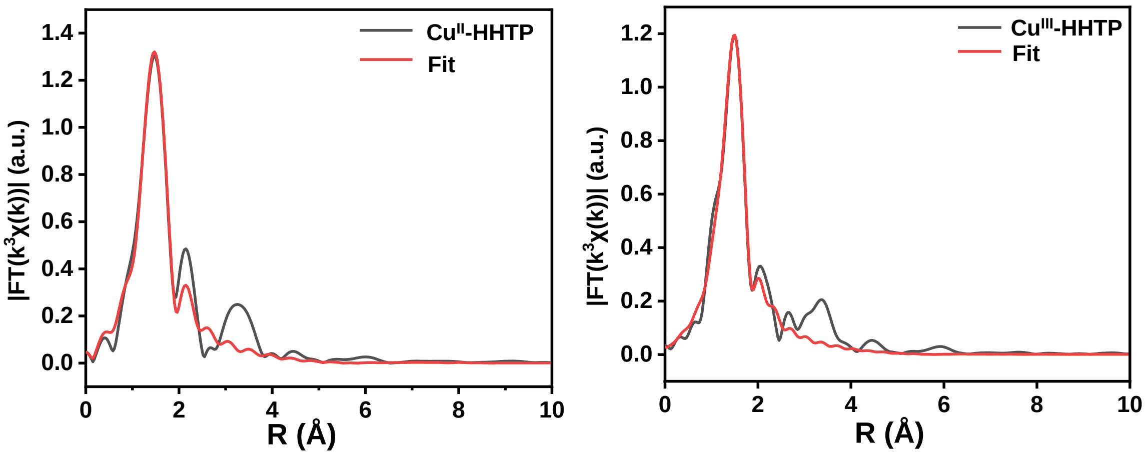

Fig. S36 Fitting results of the EXAFS spectra of Cu^{II}-HHTP and Cu^{III}-HHTP at R space

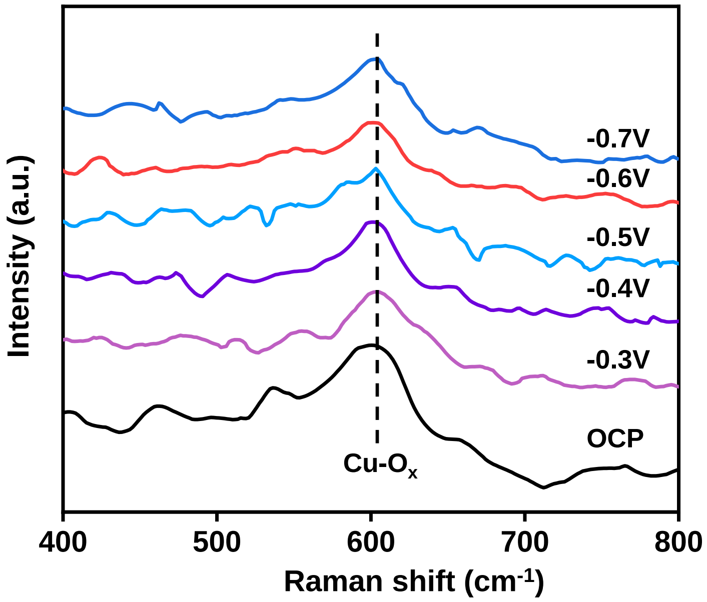

Fig. S37 *In situ* Raman spectra of Cu^{III}-HHTP during the electrocoupling of N_2 and CO_2 at various potentials

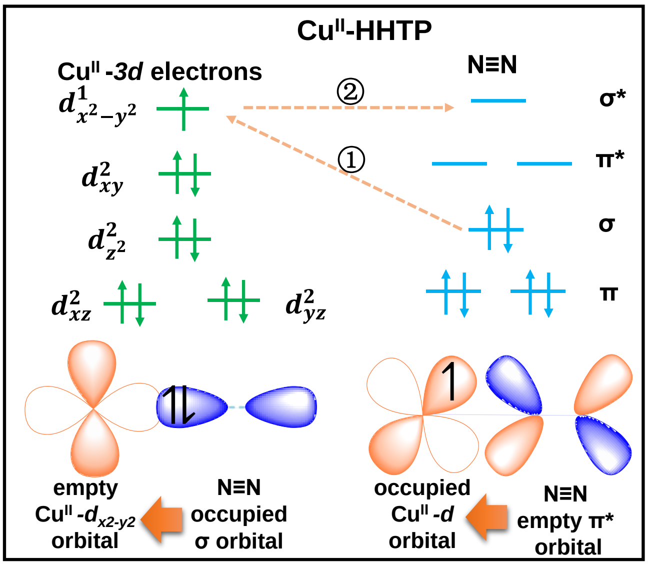

Fig. S38 Illustration of the different activation modes of N_2 interacting with Cu^{II} active site for Cu^{II}-HHTP

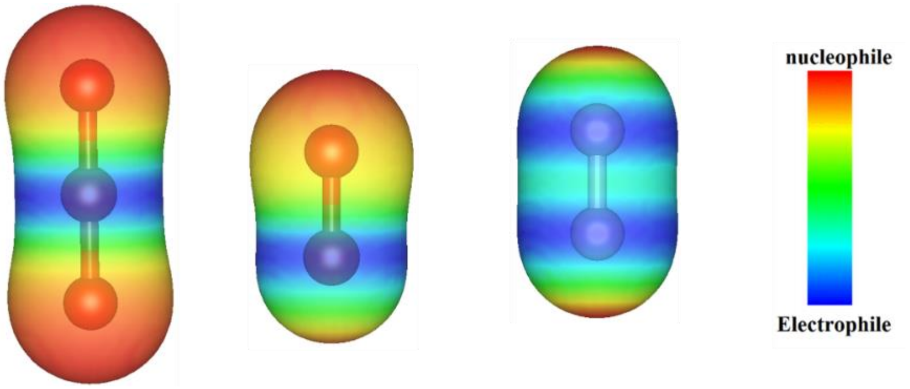

Fig. S39 Electron density isosurface of CO_2 molecule (left), CO(middle) and N_2 molecule (right), the color bar represents the electrostatic potential scale

The electron density theoretical simulation analysis (Fig. S39) reveals that an electron-rich N atom exhibits in N_2 molecule and an electron-deficient C atom in CO_2/CO. Similarly, the charge accumulation area occurs in the Cu^{III} part of Cu^{III}-HHTP and the charge depletion one is found in the O part owing to the electron transfer from Cu to O, which can also be proofed by its XPS spectra (Fig. S4). As a result, Cu^{III} and the adjacent O site in Cu^{III}-HHTP possibly acts as the active centers towards the activation and coupling of N_2 and CO_2 due to the electronic interaction.

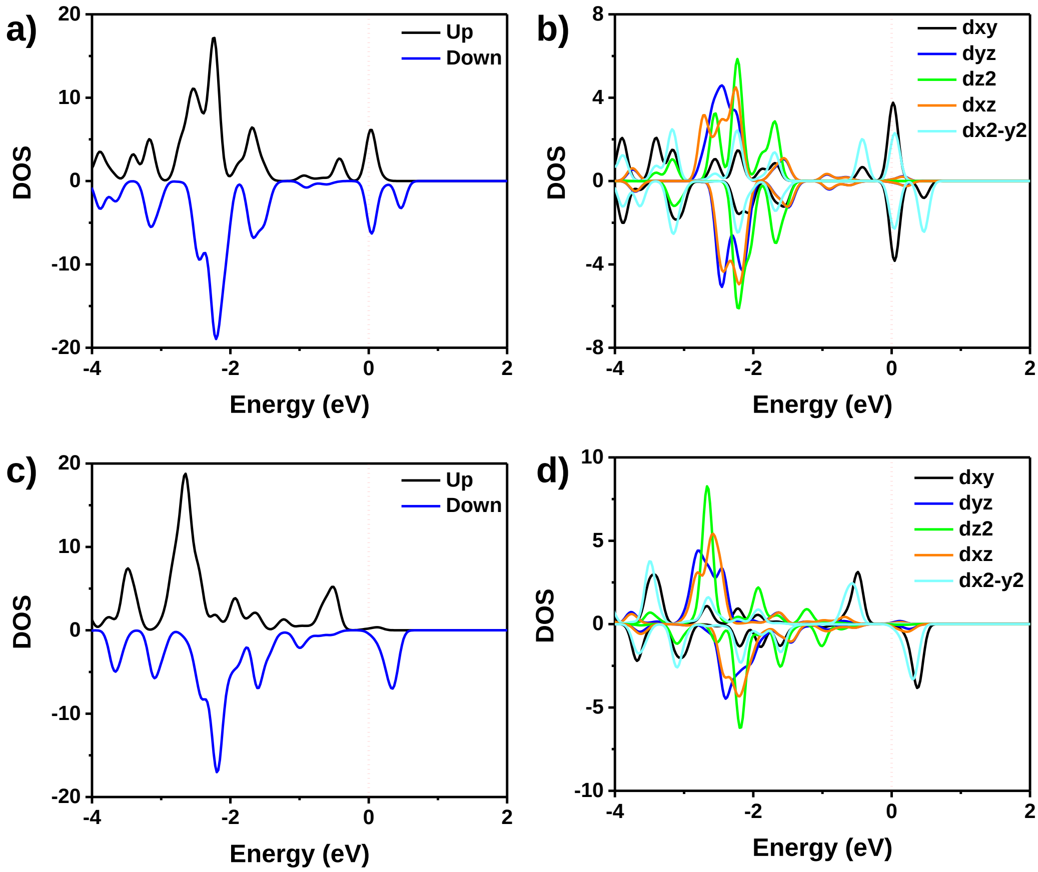

Fig. S40 DOS of **a-b** Cu^{II}-HHTP and **c-d** Cu^{III}-HHTP

Table S1 The Weiss constant (θ), the Curie point (C) and effective paramagnetic moment (μeff) of pristine Cu^{II}-HHTP and Cu^{III}-HHTP catalysts

**Sample**	**Cu^{II}-HHTP**	**Cu^{III}-HHTP**
θ	-167.18	-23
C	2.5	0.52
μ_{eff}	4.47	2.04

Table S2 Comparison of the electrocatalytic urea production activity of Cu^{III}-HHTP with previously reported urea electrosynthesis catalysts

**Catalysts**	**Reactant**	**Electrolyte**	**Potential**	**FE** [illegible]	**Urea yield**	**Refs.**
Bi-BiVO$_4$	N$_2$, CO$_2$	0.1 M KHCO$_3$	-0.4	12.55	5.91	[S1]
PdCu [illegible]	N$_2$, CO$_2$	0.1 M KHCO$_3$	-0.4	8.92	3.36	[S2]
BiFeO$_3$/BiVO$_4$	N$_2$, CO$_2$	0.1 M KHCO$_3$	-0.4	17.18	4.94	[S3]
Ni$_3$(BO$_3$)$_2$	N$_2$, CO$_2$	0.1 M KHCO$_3$	-0.5	20.36	9.70	[S4]
InOOH	N$_2$, CO$_2$	0.1 M KHCO$_3$	-0.4	20.97	6.85	[S5]
ZnO-V	NO$_2^-$, [illegible]	0.2 M [illegible]	-0.79	23.26	5.52	[S6]
Cu-TiO$_2$-V$_O$	NO$_2^-$, [illegible]	0.2 M KHCO$_3$	-0.4	43.10	4.16	[S7]
Zn nanobelts	NO, CO$_2$	0.2 M KHCO$_3$	-0.92	11.26	15.13	[S8]
Cu^{III}-HHTP	N$_2$, CO$_2$	0.1 M KHCO$_3$	-0.6	23.09	7.78	**This work**

Table S3 The ratios of Cu^{3+} and Cu^{2+} estimated by linear combination fitting (LCF) in the samples during -0.6V vs. RHE for ever-increasing eletrocatalysis time

	**Fresh**	**0.5h**	**1.0h**	**1.5h**	**2.0h**
Cu^{3+}	100%	86%	74%	71%	69%
Cu^{2+}	0%	14%	26%	29%	31%

Table S4 EXAFS fitting parameters at the Cu K-edge for various samples

**Sample**	**Shell**	**N ^{a}**	**R (Å) ^{b}**	**σ2 (Å2·10^{-3}) ^{c}**	**ΔE0 (eV) ^{d}**	**R factor (%)**
Foil	Cu-Cu	12*	2.541-/+0.004	8.64-/+0.57	3.88-/+0.71	0.4
CuO	Cu-O	4*	1.951-/+0.012	5.19-/+0.81	-1.80-/+1.57	1.6
Cu^{II}-HHTP	Cu-O4	4.2	1.961-/+0.021	4.81-/+2.23	-0.91-/+2.79	1.1
Cu^{III}-HHTP	Cu-O4	4.0	1.963-/+0.006	6.25-/+0.41	0.24-/+0.88	0.6

^{a} *N*: coordination numbers; ^{b} *R*: bond distance; ^{c} σ^2: Debye-Waller factors; ^{d} ΔE_0: the inner potential correction. *R* factor: goodness of fit. S_0^2 was set as 0.95 for Cu data, which was obtained from the experimental EXAFS fit of Cu foil reference by fixing CN as the known crystallographic value and was fixed to all the samples.

Table S5 The detailed structures of DFT calculations

1. PATH-way

1) Cu^{III}-HHTP $CO+N_2$

$CO+N_2$

1.00000000000000

21.8798999786000010 0.0000000000000000 0.0000000000000000

-10.9349554910000002 18.9466972881999993 0.0000000000000000

0.0000000000000000 0.0000000000000000 20.0552997588999986

C O Cu H N

37 14 3 13 2

Direct

0.2351540743308887	0.5937259600732726	0.5318127880943928
0.5002144994936085	0.3739048054629709	0.5195145916775885
0.3129638712883224	0.5402472860964054	0.5252131160353037
0.3638237605059209	0.6628906264106521	0.5578717966820547
0.5719396027588837	0.1228312271487815	0.5249047307915046
0.4219569579245038	0.7917172155884087	0.5709607210721983
0.2965398144092964	0.7258554417234470	0.5365980003131967
0.8315707855603289	0.4496748639932521	0.5253399218417729
0.1666136519679294	0.6555142579299003	0.5270908686980522
0.7027316661452344	0.3839221063322057	0.5239000929182336
0.4351532181283101	0.6060864519411532	0.5606043162101562
0.6312364550668377	0.4421468740264676	0.5390597410469607
0.5711043014392110	0.3161395343314198	0.5081999726587817
0.3607711206544517	0.8542836578077294	0.5309966985166676
0.5115217763127725	0.1876929125265773	0.4907545039950632
0.6381189455020050	0.2511564637716162	0.5185900895612109
0.1068324192984299	0.5257374601819832	0.5238258634656390
0.7683587279070020	0.3192203046233808	0.5189282323418208
0.5731458889965311	0.2512312758789930	0.5033720729955213
0.3783815057878742	0.5417146132330747	0.5345094574549754
0.5075358498718320	0.3148343183824202	0.5034088839956203
0.6357358519288759	0.3824370329353465	0.5244744416107271
0.1038994147947104	0.5909270850294216	0.5236358924571857
0.7664260321175834	0.4478211867572744	0.5264207025505743
0.7038974633244346	0.3177885869007626	0.5206300374006688
0.4240526267860196	0.8551726819592821	0.5573110465103732
0.6358090743287756	0.1870884439688421	0.5301114973259510
0.3615852883086348	0.7273465946834298	0.5574291658793433
0.5661208477549199	0.4413499856888479	0.5378513846378448
0.4277403876190650	0.6640549414475451	0.5704393624636571
0.3039072202953634	0.5975917458238994	0.5380234415659356
0.8326626398799610	0.3833190284291283	0.5211023528266481
0.1730871615051929	0.5291953892770201	0.5279649302458539
0.2322872991512927	0.6587377832060832	0.5313348200200001
0.5086241122666340	0.1232766566730540	0.5018101616873735
0.2983003240025380	0.7894660918836087	0.5230974155779510
0.5273294977453348	0.6000794048455977	0.6221450627325363
0.0471611637235062	0.4671112068080360	0.5214799570669711
0.3676859449391772	0.9149927723323320	0.5161525367480022
0.8921722998545191	0.5071845049227464	0.5269376754432544

0.4415820141699285 0.3711230984372968 0.5225142636656855
0.5644359682598636 0.0618372661029405 0.5396401306976224
0.4510943073564760 0.0629197188983910 0.4960355018385739
0.5603430297671776 0.4944405073927500 0.5539865482169731
0.8940405440220084 0.3881726135561498 0.5189374498831502
0.4805025067669794 0.9160770437068760 0.5639877387864383
0.3819787004640967 0.4859106819684099 0.5198353687731734
0.0411689439195453 0.5842181605770149 0.5215268449867159
0.5050622501369860 0.6189494045539097 0.5669842809017479
0.5860677443663332 0.6302205857671213 0.6437177771813007
0.4932022636624138 0.4967326466925301 0.4400649127389439
0.4656755380957024 0.9890451840224346 0.5289915907349004
0.9690291333575152 0.4864027490765626 0.5216610301395405
0.4662608594915683 0.4807818397289799 0.5292671760571392
0.4748171729698685 0.7124081914560473 0.5857556672826071
0.2522151635160618 0.7904622696395855 0.5039388610617251
0.1731458156157589 0.4794248670556092 0.5299675468857669
0.4584686071797027 0.2668425774676857 0.4916716559932002
0.6824433259369551 0.1850368095691964 0.5457456066351831
0.7678398288662115 0.4982986437344562 0.5278015014891239
0.6774849165656812 0.4916562687858060 0.5536853316778868
0.7708453330877393 0.2708904932174004 0.5142547512623210
0.4623987485456961 0.1853940443435232 0.4766252174010754
0.2698697720301614 0.4904658195758736 0.5065046425857610
0.1626367604309701 0.7031779697745316 0.5289674146880671
0.4709710166079474 0.7954138894473338 0.5877912165246620
0.4522209910174851 0.4861398907293072 0.4126718847359655
0.4709459472925703 0.5329459777334112 0.6545573971001654
0.4461488668238441 0.4781517339525270 0.6245622189569684

2) Cu^{III}-HHTP CO+N_2H

CO+N_2H
1.00000000000000
21.8798999786000010 0.0000000000000000 0.0000000000000000
-10.9349554910000002 18.9466972881999993 0.0000000000000000
0.0000000000000000 0.0000000000000000 20.0552997588999986
C O Cu H N
37 14 3 14 2
Direct
0.2339594895692932 0.5878723220754704 0.5229113656856982
0.5034122387600692 0.3810465771521267 0.5237218110497901
0.3041136523947449 0.5287234500061435 0.5495392046874945
0.3626732206617944 0.6572998685959420 0.5505046499031889
0.5683954479038795 0.1224430944941897 0.5094430732508252
0.4272763487535661 0.7865038912208289 0.5351180070384977
0.2981061404990021 0.7206489887022126 0.5175901865838366
0.8307764759979187 0.4489070686679241 0.5094917149817603
0.1678144507260267 0.6513891819599914 0.5081941720348789
0.7020530498988721 0.3837980248862649 0.5143034699078755
0.4215733474863538 0.5978187235783989 0.5963332019076981
0.6340820680938317 0.4422974055848773 0.5428382585520279
0.5690561127943065 0.3182364715424709 0.5054574832964920

0.3630444674136383	0.8492301035680234	0.5061360323460762
0.5061204849024329	0.1885319114526537	0.4890645293565424
0.6347757644363938	0.2513435910810851	0.5085997896898891
0.1055986590830093	0.5216072346800078	0.5105679297406029
0.7659645348511274	0.3181105999138207	0.5071827391485563
0.5691524939147776	0.2523835891883999	0.4987596229223211
0.3631997295969381	0.5292026775194657	0.5800904851015821
0.5067746035363460	0.3209779396396669	0.5027400971616176
0.6357021116422755	0.3830325669313977	0.5209575084034881
0.1041935534256251	0.5874450193984045	0.5059624976599543
0.7660029809466665	0.4476255826184637	0.5126918066038696
0.7018469676591444	0.3173429821399085	0.5101514737648806
0.4288568627768658	0.8502582627997098	0.5223253998081197
0.6323547879557349	0.1867398656856606	0.5152586992752328
0.3638757792986332	0.7222002413129553	0.5343987993190572
0.5695916586576391	0.4419825221024385	0.5509483298564427
0.4222489958875823	0.6589439957391745	0.5790514555149929
0.3003773996167215	0.5899106139967728	0.5395602986052055
0.8308086348790105	0.3820601408998486	0.5076695386171423
0.1709513545138448	0.5239830229933189	0.5184642298782206
0.2327968590206508	0.6537401216176953	0.5161139080353823
0.5036297436732444	0.1234133821878027	0.4937437468883499
0.2998801730805123	0.7843522847580265	0.5035994366646359
0.4957767978178519	0.5561275175277595	0.6438129110811766
0.0452252974494182	0.4635772771604876	0.5090196815732470
0.3681768864883030	0.9100488872351193	0.4943227092651808
0.8919176277228515	0.5060936986217318	0.5077850508465886
0.4475885895847107	0.3854007758502558	0.5241840660495212
0.5627987260014609	0.0611161666271999	0.5171656746196246
0.4464970296989772	0.0625094205448865	0.4872561594839507
0.5649225034399796	0.4898931797334719	0.5819816862542396
0.8919287331177848	0.3861369846102098	0.5054982578572297
0.4859290517083689	0.9114603721901127	0.5234718642980053
0.3648917079890657	0.4727867266002146	0.5948220230315514
0.0420898323337714	0.5817613393856289	0.5007506736981041
0.4764879536235315	0.6054158025160418	0.6349559642581923
0.5332213872106534	0.5591650433892476	0.6887578533802384
0.4849874713118977	0.5384718907907420	0.5056479586746406
0.4637949888515684	0.4766937783958780	0.5791896457879676
0.4653254666160910	0.9861575159691238	0.5046523658764196
0.9680644765830148	0.4842260529244785	0.5048024172780073
0.4686915016713702	0.7086384506386079	0.5927361968306394
0.2523895017089353	0.7855556704406866	0.4896502715970490
0.1692490961050633	0.4735763993008827	0.5235673609574606
0.4568211089352373	0.2750596209147341	0.4886015384718597
0.6795687282985976	0.1837260554844951	0.5261011034024622
0.7674192536848155	0.4981281316583393	0.5128357437215306
0.6816587120448406	0.4892851822931040	0.5598526873741696
0.7681526881649727	0.2695761678998447	0.5032413477878921
0.4563427183890383	0.1871357672011953	0.4801066076632658
0.2593363468099384	0.4765495051412381	0.5391699802743404

0.1647960968836595 0.6995182874647587 0.5053646319194772
0.4776033724393301 0.7894018016157086 0.5450120023785616
0.4431990233945568 0.3657091204320024 0.7290895369738868
0.4423014759484051 0.5191684413623943 0.4784176806850883
0.4671146432658630 0.4066088543117785 0.6938316613913224
0.4282501983975843 0.4069064520760982 0.6522296360148898

3) Cu^{III}-HHTP NCONH

NCONH
1.00000000000000
21.8798999786000010 0.0000000000000000 0.0000000000000000
-10.9349554910000002 18.9466972881999993 0.0000000000000000
0.0000000000000000 0.0000000000000000 20.0552997588999986
C O Cu H N
37 14 3 14 2
Direct
0.2368543300799361 0.5930785632545721 0.5271078206146607
0.5027660562712781 0.3766064418977129 0.5380646242645746
0.3137789127054972 0.5385879783353150 0.5216098427565956
0.3670884321618771 0.6636536562958991 0.5466248713005042
0.5717783628936229 0.1234509849241477 0.5210153555779500
0.4253830929426836 0.7925624343161656 0.5578900452677059
0.2981892897121192 0.7255515617136533 0.5300041910346865
0.8335482762892531 0.4496313724545894 0.5288487021177556
0.1680369093491842 0.6546133561938356 0.5280081594881008
0.7045823186455438 0.3851357629549381 0.5281231529561112
0.4395004561410085 0.6081441954750271 0.5472198601852819
0.6354063785056681 0.4463581315236986 0.5395985973097147
0.5720672021472705 0.3176217766971310 0.5213897787036571
0.3617681283265670 0.8539981700979614 0.5225760817760801
0.5112096320261804 0.1904665529533073 0.4968631229374720
0.6386733713829804 0.2519808974655328 0.5214517010159506
0.1082094358452175 0.5247556540562467 0.5231324166036377
0.7688960769336542 0.3193356888677610 0.5206841161246246
0.5734084172750861 0.2528810904030023 0.5113684162536668
0.3798868975935950 0.5413207975651487 0.5287040608994250
0.5082380637184571 0.3154779306824778 0.5268048173830578
0.6383178991473671 0.3848491153034687 0.5301223170637496
0.1051587076211022 0.5898282036513154 0.5261763253555092
0.7689891029115692 0.4485560425032595 0.5309658252138593
0.7048166046776020 0.3185221737392009 0.5238812966008396
0.4263703644791972 0.8555901605854402 0.5456358990290433
0.6360431870705027 0.1871864435351228 0.5277719115118419
0.3644419713882716 0.7277138731547746 0.5467421037640242
0.5708543730991300 0.4464479136579728 0.5416652197727433
0.4322869126376028 0.6661325224073967 0.5559119049222123
0.3058381509188813 0.5971555797857375 0.5310480487754135
0.8337189288215581 0.3830293228452354 0.5229813882518193
0.1746069963400733 0.5284618641094955 0.5240834437194615
0.2337426890514772 0.6580132722562280 0.5279740057809436
0.5080683343227955 0.1252549109997472 0.5020915908919769
0.2989437766154213 0.7886788342210975 0.5170988830413799

0.5184810296914573 0.5618403372245462 0.6513594964920975
0.0484687040706067 0.4661109626482707 0.5209508163361863
0.3674832404857561 0.9142767929557502 0.5083864408875068
0.8946147915751523 0.5067926099859813 0.5309997348116522
0.4452218068603868 0.3746387560186924 0.5465564222700893
0.5646556296195228 0.0616688559142941 0.5302553982947744
0.4505156389100993 0.0652452945718505 0.4939152417838096
0.5678857691535514 0.5025952840597915 0.5489594019732319
0.8946606531447898 0.3869947088909543 0.5199432221380997
0.4827634319985539 0.9168672396909054 0.5509467309702748
0.3836986818251115 0.4848736847217704 0.5187648851154049
0.0425673652298090 0.5831515421226650 0.5277422552387866
0.5049142526003334 0.6130116601136708 0.5527409257743388
0.5530896213613398 0.5635254014247562 0.7009891225009922
0.4838513246496434 0.4819230833382630 0.4461763673942792
0.4712181243589841 0.4871010814887052 0.5362242394738540
0.4656895740283307 0.9895541079954852 0.5200199732411698
0.9702992507316798 0.4850675104371812 0.5247374364560438
0.4796552608672072 0.7151975760928135 0.5689639515977907
0.2517375930615647 0.7891724848313817 0.5006167858252364
0.1746232141093866 0.4786451839388373 0.5238834012377582
0.4582813880144139 0.2660897618519108 0.5246760862380532
0.6827198198831095 0.1836286146131244 0.5398388038381176
0.7714032682843950 0.4994106251295956 0.5341509852505366
0.6830343355018690 0.4973535942976638 0.5461025120797495
0.7708831575782882 0.2708235202170474 0.5151494100705832
0.4625469624512610 0.1899754056922088 0.4843878368181330
0.2693217793428669 0.4872405533306517 0.5082612385951614
0.1640403533070161 0.7021893507961854 0.5315412779124925
0.4752089711742379 0.7967119674387463 0.5723786448267603
0.5819194301386817 0.6636752426713627 0.6203769000825227
0.4374053779433869 0.4608676863417797 0.4252058736428624
0.5296481878140727 0.6254516571134181 0.6216800263772494
0.4639336581839024 0.5002944831561216 0.6272130685483910

4) Cu^{III}-HHTP NHCONH

NHCONH
1.00000000000000
21.8798999786000010 0.0000000000000000 0.0000000000000000
-10.9349554910000002 18.9466972881999993 0.0000000000000000
0.0000000000000000 0.0000000000000000 20.0552997588999986
C O Cu H N
37 14 3 15 2
Direct
0.2367180159018229 0.5929000583674781 0.5277839383777772
0.5021404260127769 0.3758910002070910 0.5398577476887086
0.3129569250261939 0.5380043734198612 0.5194351100008214
0.3670107688828518 0.6625456595213995 0.5477264225595554
0.5723622027863972 0.1237503046286259 0.5210024819353085
0.4262409521972487 0.7918302024657101 0.5586595783056428
0.2989745214656178 0.7253341763322543 0.5309181973956691
0.8332730868038030 0.4505537878785125 0.5282235849660292

0.1686868901374738	0.6552327161151750	0.5286361281306372
0.7042612230464453	0.3855300847327566	0.5270987642462037
0.4386776374971118	0.6061610527871150	0.5457376766369207
0.6346769325943804	0.4465243320358614	0.5359710446991027
0.5715463902219258	0.3172637946563788	0.5217759405157333
0.3632035555338001	0.8537348742236583	0.5229000064669235
0.5112419547816925	0.1901466042169488	0.4964315700473562
0.6387105450635462	0.2523187571577352	0.5214489420134562
0.1080488350311452	0.5252886933721945	0.5240174138125351
0.7689854373870639	0.3201428886394562	0.5212809329772985
0.5732034033167384	0.2527630895999726	0.5113836230235167
0.3789182139757141	0.5402836695959240	0.5252459147394518
0.5075673272828392	0.3146911207242165	0.5292284117515292
0.6378114689750832	0.3849075795520488	0.5286163187527908
0.1055050801560916	0.5906549538840341	0.5267092608217171
0.7685347008051274	0.4491548062767413	0.5297737512927366
0.7047540462621118	0.3190485121610888	0.5238259671766837
0.4276563269986024	0.8551083409505051	0.5460633001233974
0.6364066220238875	0.1877177526367309	0.5279734933134622
0.3649825399313874	0.7270296891831476	0.5478935361369319
0.5699009604268550	0.4461046879102173	0.5387096094178482
0.4316984330274031	0.6640155980378915	0.5571853769603343
0.3054616685455988	0.5965403371568001	0.5309994094128503
0.8336889895805748	0.3840314781172458	0.5233106162964004
0.1741859566220842	0.5284732901018050	0.5251657524479548
0.2341405975610450	0.6580869512185983	0.5287746559733937
0.5085875789575904	0.1250913336374369	0.5013825627971258
0.3001730569358806	0.7885929112185885	0.5176749385016431
0.5124644904327185	0.5609146860285719	0.6555737761484000
0.0480569770134419	0.4669020090666004	0.5218696702388090
0.3691458408533271	0.9140212449085315	0.5082552048936420
0.8942765038319026	0.5078796992699524	0.5301363361977207
0.4451641600854225	0.3742816402181846	0.5519262413722282
0.5656517241253337	0.0621653459333315	0.5304814693474567
0.4515770082438343	0.0647513614881213	0.4924392927982710
0.5665196580461666	0.5022073559652930	0.5437362278562399
0.8947417601564305	0.3881975684722839	0.5208106298118439
0.4841451754222504	0.9163993485909137	0.5510380823337724
0.3827225869235115	0.4842328848850467	0.5125279667147187
0.0430815721613180	0.5843956111825024	0.5280199259448319
0.5033354598700116	0.6098118698326677	0.5514314733167655
0.5385741076509115	0.5683630308970136	0.7118997325837794
0.4810981398330996	0.4769421477171523	0.4434406972845206
0.4696117669832394	0.4863885097893804	0.5325894896728484
0.4671374790083923	0.9893186145977901	0.5196473705407896
0.9702575092425051	0.4863919565912499	0.5250525827954846
0.4789910680874720	0.7121725352373885	0.5723283174936178
0.2531454009244766	0.7893418616847208	0.5010316266743906
0.1738783811830084	0.4784915410685670	0.5254166422539920
0.4578877932259404	0.2648990063759774	0.5294648921087705
0.6831849010075921	0.1844337430856287	0.5402235051250708

0.7706439822031665 0.4998852912510201 0.5325553448308590
0.6822034963407767 0.4979929188134307 0.5401266867521383
0.7712223228236748 0.2717036399029337 0.5164613271649294
0.4626380942838832 0.1896117921543981 0.4834917816362325
0.2683035255270057 0.4872840329269779 0.5048295880031829
0.1651057211756016 0.7030554352419865 0.5320494777738427
0.4759247388606683 0.7955381517446918 0.5730525989286156
0.5804660066606723 0.6588871915901839 0.6185474965933977
0.4362863618402930 0.4649878489501881 0.4222094369476557
0.4580409372024887 0.4574379587447083 0.6539117501253296
0.5272955964105822 0.6240179722107811 0.6207774084752774
0.4652765326029984 0.4997443808605969 0.6259943335451250

5) Cu^{III}-HHTP $NHCONH_2$

$NHCONH_2$
1.00000000000000
21.8798999786000010 0.0000000000000000 0.0000000000000000
-10.9349554910000002 18.9466972881999993 0.0000000000000000
0.0000000000000000 0.0000000000000000 20.0552997588999986
C O Cu H N
37 14 3 16 2
Direct
0.2368372072754220 0.5940696087967149 0.5265467564809294
0.5034650960390952 0.3751143282602604 0.5434120025928157
0.3126625617346822 0.5383959700426009 0.5199452150747300
0.3665368590648340 0.6630586967554996 0.5474197053393397
0.5731064144777371 0.1235196967000504 0.5200016782152677
0.4255639317834605 0.7922461753621434 0.5580046746052244
0.2986242369176141 0.7262408750739409 0.5301549688047594
0.8337455455209963 0.4503523083632996 0.5269579757811340
0.1682621920529470 0.6558456792243359 0.5273558929750888
0.7049064314837908 0.3852362972839119 0.5247017221027814
0.4362314861070789 0.6048231851758362 0.5506066907198947
0.6351483017121844 0.4463332038565250 0.5316195961486525
0.5725275819362354 0.3170155095426229 0.5212935770866182
0.3626558902967207 0.8547070533669752 0.5229287968725194
0.5123310553904855 0.1902900037264082 0.4951694053862188
0.6396716283979259 0.2520349882603707 0.5202774839427617
0.1082678856874853 0.5262296515079129 0.5229817692629305
0.7698954905515483 0.3198908398103707 0.5207052051093285
0.5742270275940671 0.2525861954155124 0.5104296824992673
0.3773248089673500 0.5390712753247697 0.5298671643339520
0.5085826404404600 0.3139804744402102 0.5311391374916308
0.6384921533231189 0.3845884736343587 0.5259128783945686
0.1053815840725761 0.5913094777039212 0.5256841043345563
0.7689813121775070 0.4488520100286892 0.5276083818730362
0.7055379842851307 0.3186565755113459 0.5224447534795662
0.4269786467625655 0.8556776775837449 0.5457906711329668
0.6371964420543224 0.1872621223047967 0.5268388956372234
0.3644785269840417 0.7277723152134487 0.5470742057395149
0.5704933701040916 0.4451491927716565 0.5395264573508428
0.4306889462152803 0.6641109737855112 0.5580349291109624

0.3054630038756617 0.5973847943492929 0.5306478427771119
0.8344206283848792 0.3838090716370649 0.5226771428335009
0.1744940156760075 0.5296362527251026 0.5238443259780625
0.2340733885130797 0.6590831934293738 0.5275948023152479
0.5095643411816198 0.1251721121699011 0.5000637147953230
0.2998064485698668 0.7898290298961939 0.5176456279570800
0.5207727065766807 0.5630720453222263 0.6594505254604971
0.0485408194400095 0.4674618624215883 0.5213340064346981
0.3691135707136993 0.9154504159907466 0.5085053639187381
0.8944775741677304 0.5077405148845930 0.5294482527140659
0.4466374895074181 0.3732805472310748 0.5581009291624865
0.5658682504592191 0.0616977233447093 0.5298982420462002
0.4523717544199575 0.0651144679210456 0.4910027339369037
0.5665469517964882 0.5009452793106079 0.5476557459145044
0.8956136903883137 0.3882584158785142 0.5209064219296237
0.4837559114840194 0.9165999075041372 0.5510025846017783
0.3800344211441971 0.4811938983195133 0.5219681886841526
0.0425844893802753 0.5845518505855962 0.5273973439778439
0.5016857549388970 0.6085884646017293 0.5579981523720363
0.5609483347093068 0.5681107811737378 0.7040952063254109
0.4813160623503129 0.4778655873291987 0.4423240865583728
0.4696894934897050 0.4844237489464352 0.5323202042132508
0.4672187026594298 0.9896239047672474 0.5192071907249310
0.9707136358092936 0.4866257835729388 0.5247720211790200
0.4784145133565547 0.7127232039087683 0.5715494515139670
0.2526866860252957 0.7905365521620205 0.5012512377300256
0.1742795927187946 0.4797101002032915 0.5241196371134624
0.4592175327279238 0.2638949000433186 0.5327480037368348
0.6839275044502531 0.1839009245147776 0.5391374813615638
0.7709580963953393 0.4995134821329911 0.5303843613010638
0.6825690600665995 0.4981243169395179 0.5329080712143385
0.7722622126319414 0.2714613653348159 0.5166754946286318
0.4638256319564620 0.1899769153805309 0.4822676709447192
0.2680985374386983 0.4880745544309948 0.5042856465960933
0.1644965165447008 0.7035586693307345 0.5307088500173678
0.4753912571143919 0.7961479534157070 0.5721746547566444
0.5765144631276087 0.6639473875584728 0.6269785078989828
0.4362198414831789 0.4652284002241601 0.4213183746481258
0.4607609363481351 0.4567568139522843 0.6631398456257366
0.4151330630789802 0.4959590488347554 0.6379825337799744
0.5265050303033847 0.6213492476146038 0.6259461781190407
0.4629548801849574 0.4979653698779082 0.6369593083235012

6) Cu^{III}-HHTP NH_2CONH_2

NH_2CONH_2
1.00000000000000
21.8798999786000010 0.0000000000000000 0.0000000000000000
-10.9349554910000002 18.9466972881999993 0.0000000000000000
0.0000000000000000 0.0000000000000000 20.0552997588999986
C O Cu H N
37 14 3 17 2
Direct

0.2345503879073617	0.5939855784942346	0.5199969179978063
0.5036154782117962	0.3804464809698280	0.5500300638215648
0.3058779415692564	0.5343673367416961	0.5225364399470546
0.3665556981294100	0.6640257673306409	0.5285927179259382
0.5700512855092422	0.1260519202654546	0.5214385099850258
0.4261911744211991	0.7931640383066991	0.5391350922927511
0.2980090865595177	0.7275155606564984	0.5193085033757573
0.8321678236909716	0.4525269212569173	0.5251060725722834
0.1676667796746273	0.6570572600746455	0.5191443578728878
0.7033438808109440	0.3878760708442131	0.5256093772931270
0.4366751352404947	0.6049689144748447	0.5358764523262137
0.6349261090181951	0.4500675798299184	0.5338146581689009
0.5707917922663108	0.3204535427006711	0.5269178445158542
0.3617173856433249	0.8564110149240830	0.5163325983544037
0.5084115909823759	0.1931448912844426	0.5037798267010923
0.6371263740724398	0.2546245030169302	0.5225410365656350
0.1056162347161608	0.5263329406820632	0.5188367890663935
0.7675900448029011	0.3220380781436802	0.5213696277190378
0.5714254519655451	0.2554850907024142	0.5162695481371845
0.3701051038361122	0.5352362182183616	0.5311704005109121
0.5076643675520445	0.3186346328331758	0.5385047053409823
0.6373270896959343	0.3879061793422589	0.5286484415737615
0.1038596215638399	0.5922480458028162	0.5195757491197777
0.7676989304159338	0.4514135326243598	0.5266909033357710
0.7033972491282664	0.3211209501348745	0.5236507826729624
0.4269627863303904	0.8569817934270553	0.5326141657297948
0.6345537257250337	0.1897040763572058	0.5267751903148873
0.3642452437343562	0.7288577973237915	0.5297045557218845
0.5711013437906211	0.4502447149357457	0.5436168830503605
0.4308716866491701	0.6661737747299665	0.5317795305213660
0.3014685456120246	0.5959636694388933	0.5230835463573751
0.8323019521653565	0.3858153590789945	0.5219906706919892
0.1709842238012020	0.5293780817775529	0.5191900205823875
0.2326697095445035	0.6598500018768929	0.5187825906115519
0.5055226659514311	0.1279241543197695	0.5065973827883140
0.2985115659314588	0.7912168912011754	0.5114281766985359
0.5331439100102963	0.5426916737714524	0.6804222491817999
0.0450721408343547	0.4680879804261839	0.5190219647886862
0.3663828593933157	0.9173675615748660	0.5078426148740995
0.8932576419029349	0.5099263075953135	0.5260466830007086
0.4475803707346601	0.3793399086806196	0.5662540973521334
0.5634656400984264	0.0641810066465241	0.5282632853058441
0.4477732882540365	0.0679456181415945	0.4991680556072975
0.5688138234807245	0.5067754071693692	0.5507850658710249
0.8933540177920374	0.3897902675009061	0.5199371416637826
0.4839069747659288	0.9179755080571351	0.5389146684873520
0.3733536438145665	0.4783700798541139	0.5367140468246958
0.0423325095694125	0.5870401867061885	0.5210925378074924
0.4950036032854040	0.6067728141107729	0.5443520739058741
0.5563034278288902	0.5152169974654446	0.7182662470072465
0.4748016976255628	0.4800318205868175	0.4531968325765605

0.4705378433694747 0.4918825408429788 0.5430385903896340
0.4647095582632144 0.9918284827373157 0.5181201992583011
0.9685089306480054 0.4882063690505971 0.5215232399887003
0.4805995383431582 0.7158588423258956 0.5330681330561715
0.2505427662710962 0.7928157031769318 0.5006094555963603
0.1702715438342539 0.4792600238642418 0.5201075910582930
0.4580998795650524 0.2689606174852947 0.5424853788521871
0.6816591558869689 0.1858592484922034 0.5353916171659854
0.7699834728945162 0.5022341304542633 0.5290131020026143
0.6826320872641821 0.5016295659589489 0.5329841842658265
0.7697999456583829 0.2734770029400025 0.5182982333759824
0.4594278196610656 0.1930521977244210 0.4938728514260309
0.2589260634791080 0.4824919793349196 0.5180876694289355
0.1638261150720395 0.7047953790718572 0.5204129318013880
0.4763015117194513 0.7963412096571583 0.5505936863083623
0.6109205290980751 0.6443019324649992 0.6855826581858436
0.4263347660834945 0.4575590985768417 0.4366790000379962
0.4464801820470799 0.4480428529554108 0.6613037659007736
0.4309873945181996 0.5153870797469169 0.6534566483638015
0.5476795126938414 0.6273240632845131 0.6235953876648387
0.5619001332977005 0.6123530859381332 0.6667966940971909
0.4668402673213687 0.4990518135060495 0.6450016222586548

7) Cu^{II}-HHTP CO+N_2

CO+N_2
1.00000000000000
21.8798999786000010 0.0000000000000000 0.0000000000000000
-10.9349554910000002 18.9466972881999993 0.0000000000000000
0.0000000000000000 0.0000000000000000 20.0552997588999986
C O Cu H N
37 13 3 12 2
Direct
0.2352647229385076 0.5901109568426380 0.5315370413673168
0.5025875777795290 0.3785511736765284 0.5092220038326100
0.3103427676638368 0.5324624110420872 0.5301956897579008
0.3659416075010760 0.6591770848635842 0.5476474697863725
0.5695639984203336 0.1233026579061993 0.5259027912635361
0.4250894056670950 0.7887182704132999 0.5611433084268617
0.2981636123389174 0.7229026744279409 0.5331211269884921
0.8298588923140338 0.4505275672967010 0.5284587255430293
0.1679554387371351 0.6535083944374925 0.5278324299516156
0.7008615579561881 0.3842340676456339 0.5267918529827715
0.4355588520217987 0.6005926085153580 0.5479628841050553
0.6267404995498008 0.4390717068620484 0.5489895873402758
0.5710287246194451 0.3184070351367613 0.5035230962927626
0.3616632247639965 0.8517802425520983 0.5295284285525595
0.5085039794554617 0.1883732738626202 0.4943300269245527
0.6360772139965891 0.2517712573035156 0.5178170435382280
0.1062315446561538 0.5241136187699211 0.5256255969622712
0.7667057007439968 0.3199107227528797 0.5183180132975042
0.5713137662641312 0.2522380400805824 0.5025168639632507
0.3755846210218723 0.5330638597938991 0.5354337674108787

0.5098461621044692	0.3207581005100808	0.4893930128828134
0.6335485646138475	0.3819009171475986	0.5272890808864773
0.1045034142127659	0.5897590026169999	0.5256102470897541
0.7644692162923434	0.4483133388180482	0.5306982275794101
0.7021892751407034	0.3182266487757423	0.5206423410820761
0.4260942575681636	0.8526454753317239	0.5516080828666453
0.6335793624465738	0.1876242462508761	0.5297775746898592
0.3642481412880390	0.7243630028889608	0.5487053266081565
0.5621935979764195	0.4377995210747601	0.5433958405789159
0.4307990634440886	0.6605720346831383	0.5534152078977914
0.3038788619341205	0.5927047017076814	0.5360793605881110
0.8309645882720327	0.3841577066175199	0.5221383062369676
0.1720721513755914	0.5263006033219588	0.5288757714849633
0.2334941895713721	0.6558309864809648	0.5303070368745354
0.5056239738930340	0.1236751627752327	0.5055115553733484
0.2993281658860722	0.7869203492371255	0.5224232869939174
0.5364552163899992	0.6125384859716142	0.6054037266365806
0.0457872966399738	0.4660976067965519	0.5236944060651841
0.3675200927180142	0.9129441159819970	0.5180397980195507
0.8907142810213604	0.5077452658475833	0.5304970812260165
0.4448589867878168	0.3805951472239523	0.5036999335729911
0.5625944641820296	0.0622998328692473	0.5402666018149982
0.4479944552081009	0.0630175402778489	0.5016841576291283
0.5511680943221485	0.4871769503867532	0.5660725244448201
0.8923742765868541	0.3892169884196640	0.5194857940790367
0.4820826978288512	0.9138076090275579	0.5590957717281080
0.3774289844488029	0.4745214658587170	0.5278708215710918
0.0419561712746199	0.5840328704027588	0.5243022449836596
0.5057675746588659	0.6115600007044973	0.5472260171176407
0.5974328323463043	0.6468443905120810	0.6195831466016103
0.4526962315001780	0.4609825222410358	0.5572215974033793
0.4646406144651378	0.9881377050517186	0.5302854832073353
0.9683309040905553	0.4870391903330031	0.5242208556071360
0.4799250109582999	0.7102486995072174	0.5599617063797724
0.2522026871784920	0.7880672760805129	0.5069224151440178
0.1707049607598437	0.4758120551697750	0.5303068834550332
0.4635818130051003	0.2758683524941837	0.4675989847049890
0.6804086199037939	0.1856977180126578	0.5449675538348063
0.7651606799733752	0.4984239032675572	0.5333574978116652
0.6707883446448238	0.4851138198525564	0.5721392980826032
0.7693726158627293	0.2718403186632072	0.5118692796714441
0.4588054321107150	0.1864191038793143	0.4834680876046215
0.2644484454632372	0.4807920568410921	0.5205325074840027
0.1650843239736246	0.7017686580437781	0.5295566161899395
0.4746628912633424	0.7922926518799538	0.5759255761520897
0.4842731001720488	0.5594791457909603	0.6558530417473617
0.4524371768314432	0.4981991591904272	0.6422225590277952

8) Cu^{II}-HHTP CO+N_2H

CO+N_2H

1.00000000000000

21.8798999786000010 0.0000000000000000 0.0000000000000000

-10.9349554910000002 18.9466972881999993 0.0000000000000000
0.0000000000000000 0.0000000000000000 20.0552997588999986
C O Cu H N
37 13 3 13 2
Direct

0.2350025049310500	0.5900850147325911	0.5208980689450852
0.4977345505970742	0.3772079758612699	0.5263349196546010
0.3111701394264786	0.5338642392859698	0.5265937708913505
0.3626501034046650	0.6584646307867889	0.5524856622123432
0.5703114766772804	0.1245965216704176	0.5134983174136157
0.4266320339846723	0.7886395900461876	0.5491293523220118
0.2987826807355796	0.7227291166242293	0.5223194306398266
0.8300468198252847	0.4518304753891995	0.5153147313328347
0.1683869143741891	0.6537374456108759	0.5152653282533010
0.7007244227107449	0.3865335133594910	0.5171028759636865
0.4265346281607100	0.5969316740096839	0.5798645907079991
0.6299870578825303	0.4453086499987726	0.5351123883266140
0.5671878537906355	0.3182571464480639	0.5088677037239784
0.3645399596054421	0.8512041499178893	0.5125862062929074
0.5066719252582286	0.1888967464051663	0.4913396976661258
0.6350172299810724	0.2536128480233498	0.5125777718450459
0.1060932773940840	0.5238553149342321	0.5126233989810767
0.7657208643077982	0.3214639665724822	0.5112067760827930
0.5688617717682464	0.2533314937970791	0.5023518919374328
0.3741774076597562	0.5341815560285996	0.5460962568050175
0.5029452095935585	0.3173452448071903	0.5096932341364464
0.6334285975315493	0.3851985565712029	0.5205764944348014
0.1046535435710357	0.5896954665593888	0.5125231669920668
0.7648619587125192	0.4502567705961565	0.5173553242466030
0.7013234171497617	0.3202990057466729	0.5140077557594569
0.4291713264849644	0.8523411539325294	0.5346723440195229
0.6336317447030640	0.1894662906478294	0.5196390392542158
0.3637508437608448	0.7239829870682509	0.5427756786930482
0.5647310717342217	0.4454312496554527	0.5382736938852590
0.4215976379682429	0.6570884602451342	0.5796062580934784
0.3026353277094991	0.5926348323972621	0.5321136234502059
0.8304037366099903	0.3852820918550659	0.5120422146450537
0.1718194431905883	0.5260790759937611	0.5168266427292957
0.2334367987507659	0.6557453796733531	0.5191183171446692
0.5052530798162252	0.1244635680166372	0.4965424071225547
0.3012404030645974	0.7862719999104707	0.5073817301614696
0.4884825923330802	0.5455646157785069	0.6389158323197032
0.0453782654574873	0.4660303895487998	0.5106624294701009
0.3704597972530062	0.9116164766836602	0.4983415573175676
0.8912120157266972	0.5090251784358950	0.5154907744734846
0.4391220067599118	0.3756134050375666	0.5345598936698621
0.5654820181244348	0.0636186480425244	0.5216663051151542
0.4486857669598016	0.0631257841789555	0.4894791055644722
0.5598033135509959	0.4991752688838119	0.5511337615717520
0.8916608401604442	0.3895122817519354	0.5094035853469941
0.4859460264790909	0.9137468688094743	0.5379879090304731

0.3825610634750282 0.4806747406919179 0.5349377390348869
0.0424815940446511 0.5843980638850733 0.5110610815429162
0.4841124954807763 0.6023505878833366 0.6130328289626328
0.5288934661071161 0.5570817883278670 0.6841369168171041
0.4445639727598238 0.4590679726615947 0.5872089258302692
0.4677387079353882 0.9880512033676975 0.5108982858123006
0.9679714734395426 0.4871445576961089 0.5109732402534262
0.4646218395774694 0.7039732493161037 0.6026622725128660
0.2547752017112899 0.7875830507487535 0.4898688159741882
0.1707883511204227 0.4757762285398103 0.5194594942055905
0.4531744933465271 0.2684794276695124 0.5026164731254583
0.6811869395278689 0.1872818013962365 0.5310239070984113
0.7666419743366033 0.5009284041846302 0.5183819219888580
0.6772582728191896 0.4954639374371241 0.5461328343388032
0.7680380478695110 0.2730160704199084 0.5070897719696731
0.4567871549424007 0.1865802462597847 0.4805650882232455
0.2695661676883739 0.4841828362468393 0.5060879028113281
0.1655066793898337 0.7020214049935773 0.5167183484076953
0.4760962359433908 0.7911958406548212 0.5625449546782674
0.4496167957597569 0.3761422189944312 0.7463376965471814
0.4674475651100515 0.4144603494743935 0.7084515848220250
0.4252008879830663 0.4021327528206676 0.6650815003975175

9) Cu^{II}-HHTP NCONH

NCONH
1.00000000000000
21.8798999786000010 0.0000000000000000 0.0000000000000000
-10.9349554910000002 18.9466972881999993 0.0000000000000000
0.0000000000000000 0.0000000000000000 20.0552997588999986
C O Cu H N
37 13 3 13 2
Direct
0.2362376434540668 0.5880558219604890 0.5268139633719603
0.5041625284660346 0.3815732124269771 0.5225579462357822
0.3109714759726960 0.5305234191862224 0.5249780345004957
0.3681227608193832 0.6581692955430503 0.5383023480327849
0.5686936863559641 0.1228550315667643 0.5229395208569256
0.4276488787274018 0.7879265031179851 0.5490293150807748
0.2988928069322985 0.7210423159347102 0.5292708104163608
0.8309214405330829 0.4488082991710609 0.5303926812772425
0.1685627421953947 0.6510657689787351 0.5297723706489771
0.7018746107358447 0.3840181735917426 0.5309492167125951
0.4387428568182806 0.6004820220980615 0.5374213324847771
0.6310049780211181 0.4415348649638693 0.5554262864348748
0.5704207152755035 0.3192828180144548 0.5130615693635578
0.3616881556558330 0.8501304479637442 0.5255241903882453
0.5067818149420806 0.1893064962014794 0.5014914699890626
0.6354289528302737 0.2516882663959284 0.5201474860632364
0.1070585734903440 0.5214443132524562 0.5237573056838851
0.7662331360091451 0.3186008399578436 0.5190025568253427
0.5701318200855153 0.2529001765008482 0.5093670148591448
0.3768578878496857 0.5320471053328499 0.5292829760088361

0.5088763214446881	0.3221668616005495	0.5030151094459797
0.6350290846062038	0.3829776409986294	0.5341380192458755
0.1050835282594796	0.5870104035799288	0.5278620983569948
0.7660089821314056	0.4475403411510778	0.5340679910911593
0.7020759293918496	0.3177147773558344	0.5230812863803658
0.4276792070725890	0.8515200002501609	0.5420032952485873
0.6329011166975674	0.1869604549567310	0.5273442801160113
0.3660286148624818	0.7229145075801990	0.5399485740940856
0.5674452078580401	0.4431794234582275	0.5505302609909544
0.4335044582399032	0.6601910394942586	0.5427835945876311
0.3049364241877393	0.5908868424775446	0.5291753683659063
0.8310302877997181	0.3822203374000075	0.5228391137080196
0.1730862024294602	0.5240838082205986	0.5238323459051776
0.2340698246892410	0.6536854944762089	0.5283235016769819
0.5039297649276002	0.1240654858321469	0.5078069591089581
0.2990416447851124	0.7847247926617578	0.5208128630715448
0.5217622512908294	0.5835249839207395	0.6524284103478529
0.0466342644171919	0.4635258658874958	0.5212007961536144
0.3660573752807588	0.9108678670211228	0.5164356158723223
0.8921388021027057	0.5056692426062063	0.5322642579334365
0.4468389432801319	0.3842249858584186	0.5217230805891764
0.5623416921102083	0.0613391477638083	0.5323108294766512
0.4462266096175503	0.0635089909858894	0.5035532463931455
0.5604547000909992	0.4959739825581312	0.5674732599770825
0.8922017055011831	0.3865641710856410	0.5192467926243574
0.4838084592133438	0.9130590585243875	0.5473917464797384
0.3802552357748124	0.4745675012153289	0.5265207902853264
0.0426844118841725	0.5810587662367825	0.5297940045137364
0.5026750946418613	0.6026679565788118	0.5403715029143785
0.5552726165751077	0.5998996835910015	0.7057594853397013
0.4583650262482260	0.4704580043142078	0.5663197357435826
0.4638916272898558	0.9872530517295737	0.5249946446641015
0.9687495921976970	0.4839071392167582	0.5253790925194968
0.4823874076724619	0.7096963419071999	0.5504283899948034
0.2508649365709331	0.7856021034099403	0.5091783501491974
0.1720554891118936	0.4737870335143224	0.5222793590062341
0.4607390132476434	0.2769158013716337	0.4844384848610758
0.6800677660517095	0.1840478275712016	0.5387437750466860
0.7677236830038030	0.4980868928011367	0.5376627406153222
0.6768301759107224	0.4879225830274894	0.5759850540684864
0.7681662709313958	0.2702623291188127	0.5115224040098004
0.4568394132647832	0.1879257186350487	0.4938789559180952
0.2647466728620084	0.4782951563451602	0.5188995071483203
0.1653389127787055	0.6990137071043649	0.5337696479968896
0.4782293032595769	0.7918459256903961	0.5597329196770696
0.5921783464202834	0.6618080775739431	0.5909583804592728
0.5394338859617097	0.6365653146366631	0.6024949889911849
0.4646958178826986	0.5194273235452437	0.6424970576007645

10) Cu^{II}-HHTP NHCONH

NHCONH

1.00000000000000

21.8798999786000010	0.0000000000000000	0.0000000000000000
-10.9349554910000002	18.9466972881999993	0.0000000000000000
0.0000000000000000	0.0000000000000000	20.0552997588999986

C O Cu H N
37 13 3 14 2
Direct

0.2380109811898911	0.5909893560029359	0.5296174426467724
0.5074693628606946	0.3824981135337938	0.5038548118417713
0.3135961408151556	0.5348701501072840	0.5220994659573417
0.3683394031746751	0.6597785897071766	0.5503106505419320
0.5721535014164096	0.1245870411766850	0.5254805782401324
0.4283287847998857	0.7897400402362957	0.5614920227997909
0.3011440921272578	0.7236564410315123	0.5341415800763863
0.8330942754494952	0.4509579646627338	0.5272893268065514
0.1707378993610442	0.6543393512495549	0.5306158397139988
0.7043011539472922	0.3854182568651506	0.5245104978739280
0.4384823851969541	0.6013242507901220	0.5511262677766939
0.6317253463680480	0.4416805842565408	0.5451866209899537
0.5741787853619632	0.3202652585465616	0.5008824234890613
0.3651109344768224	0.8525105100561541	0.5296126158425855
0.5112803632395028	0.1898887758071629	0.4933987272273149
0.6388071864276256	0.2530609759111724	0.5170647112328991
0.1091402850566032	0.5245056222801141	0.5251523401023513
0.7695415300131621	0.3203819751739487	0.5181715978214064
0.5741152287516778	0.2537357672052874	0.5012555568762430
0.3792308560773734	0.5359897010366409	0.5280765297596789
0.5138121737398434	0.3233635030761680	0.4854632090950601
0.6372940271890803	0.3838769267357278	0.5244803132073343
0.1072094477218806	0.5901862373939933	0.5280901904326023
0.7679471032309881	0.4492904641519638	0.5280677024134566
0.7051356126606840	0.3191363617640397	0.5195717992684197
0.4295779521064487	0.8535805718409502	0.5514384737937602
0.6360596216446444	0.1888263805328539	0.5293981316761448
0.3669166888200585	0.7248659552737571	0.5505126826080743
0.5681762513799006	0.4426261683054896	0.5370102034195827
0.4323374884783595	0.6600488191888658	0.5612196812453409
0.3065288332011964	0.5938332841801027	0.5333549915019211
0.8338697041903124	0.3843525296677422	0.5219395234103302
0.1750339864873943	0.5269799826135145	0.5266081624266539
0.2359956449620819	0.6566042576452575	0.5309670308959752
0.5082240420192171	0.1249880525006241	0.5048485202949463
0.3024476863382357	0.7874107967904952	0.5228371921905968
0.4902903117399152	0.5563263738085382	0.6699221788300387
0.0487454788038040	0.4665830208796462	0.5225173029827298
0.3705339132613975	0.9132324154092503	0.5175283904083028
0.8939661966467394	0.5080548532238472	0.5298666113687898
0.4508945113156910	0.3856622560785415	0.4984816280037799
0.5651243696617751	0.0635823286092941	0.5398829159825302
0.4507503442966003	0.0643475154623288	0.5009808006973887
0.5592056620945823	0.4938103885721920	0.5552826107529754
0.8952913788975158	0.3892247571723727	0.5201280816468773

0.4856499171735084	0.9151132520077538	0.5581228352379188
0.3849005237765318	0.4815879936006272	0.5129388173895475
0.0448805298222876	0.5843564784895068	0.5291397107458607
0.5025332837052094	0.6039227109454535	0.5588974566474481
0.4970806785268389	0.5685989684624435	0.7309149292272836
0.4600813044318213	0.4705374553689588	0.5509794247824176
0.4675667645661412	0.9890792987101128	0.5295284921164350
0.9710679695483467	0.4869484055086483	0.5253064032632612
0.4797792348174747	0.7085681653860137	0.5757917478610818
0.2554405582581534	0.7885655831338491	0.5068910295698534
0.1741579498999835	0.4767165646056652	0.5267884848439709
0.4673310468479710	0.2784680839185291	0.4638997253128782
0.6828280718473418	0.1869115635622852	0.5448383623449824
0.7690231105157191	0.4996228896026919	0.5295446417509593
0.6759572403256955	0.4875331084121660	0.5683655211072945
0.7719554574520865	0.2720844776305681	0.5126518085897311
0.4616732379450509	0.1880251439231264	0.4821579672207396
0.2687751701727434	0.4846814241058109	0.5065051102685456
0.1676702578364576	0.7024205424048514	0.5343637741568928
0.4781301255014141	0.7932683335664050	0.5751856298895357
0.4322849543532339	0.4536109194784796	0.6757159596689253
0.5329593097534702	0.6600531265055027	0.6459020479737227
0.5288240388821607	0.6146978362722534	0.6267261538803253
0.4505125550404802	0.4931247508839295	0.6409360589799720

11) Cu^{II}-HHTP $NHCONH_2$

$NHCONH_2$

1.00000000000000

21.8798999786000010	0.0000000000000000	0.0000000000000000
-10.9349554910000002	18.9466972881999993	0.0000000000000000
0.0000000000000000	0.0000000000000000	20.0552997588999986

C	O	Cu	H	N
37	13	3	15	2

Direct

0.2368943102361367	0.5915920387647420	0.5256495501357700
0.5057072587698395	0.3809916124344173	0.5103361218002395
0.3114269549465543	0.5342227188264231	0.5210442524287248
0.3667072370123995	0.6594299920571345	0.5479193130956258
0.5710945951638621	0.1241855259724721	0.5242366554248453
0.4278243542056008	0.7895416039754648	0.5549049477817678
0.2997215967870842	0.7238571852594029	0.5318366635851466
0.8328011687177567	0.4505083298791622	0.5262861200964537
0.1691771317566776	0.6542878198983235	0.5282793544712023
0.7039178642556368	0.3853393885357356	0.5237303942194402
0.4337114274783216	0.5985819381240240	0.5553218502186777
0.6328959465159825	0.4438546842993955	0.5412895068482149
0.5731056212163147	0.3197880803227128	0.5036807583508667
0.3634504377764537	0.8527422995304271	0.5281221504745857
0.5099194265123120	0.1896035324447215	0.4953481325234810
0.6379087319641664	0.2527737609628645	0.5168488958210931
0.1080634843419327	0.5248393785014637	0.5212090907683840
0.7687109169112595	0.3200762404120132	0.5172058786353745

0.5730221715301175	0.2534904731255550	0.5029778006828352
0.3757488016791962	0.5340596213324909	0.5308620033324520
0.5113733872530503	0.3218031172428112	0.4922092446737822
0.6372106711925448	0.3843222589279498	0.5238005953496727
0.1058812751848903	0.5901875979807698	0.5255047657800591
0.7678997529645749	0.4490554498674197	0.5271717481207101
0.7044424571005248	0.3190338661830183	0.5190133855267912
0.4288087036062321	0.8534546586993571	0.5462207615768311
0.6352387897690043	0.1884468838311600	0.5278637998518960
0.3658359176889854	0.7249231418453090	0.5464398070856956
0.5691442035613379	0.4445218363765434	0.5357087802334299
0.4295649614375461	0.6587332695633404	0.5623031554452155
0.3051815439669278	0.5940104357529137	0.5306514242254107
0.8333295364749476	0.3839801154941989	0.5207581317455152
0.1740620379372264	0.5275868216901929	0.5214659528567284
0.2347757154419369	0.6569390889246732	0.5282641976098112
0.5069084127674470	0.1247747600008595	0.5053372366664284
0.3007445835729293	0.7878380635444264	0.5222842575295150
0.5091344772190672	0.5580076605278912	0.6754113198327014
0.0478701219755935	0.4665364262985700	0.5189559789353703
0.3686945597074575	0.9137915646213561	0.5179292417653832
0.8937936958387046	0.5077099555098993	0.5290750884127824
0.4473570483015218	0.3815602745594150	0.5101459551136670
0.5643944110587424	0.0631148524404941	0.5375351275467624
0.4491226320653864	0.0641149288542581	0.5015168243887668
0.5617523125509212	0.4977804745484132	0.5508612404047589
0.8945874530332482	0.3884583629558956	0.5188335622945310
0.4854319663082005	0.9146792272994888	0.5512786558303624
0.3808665770305917	0.4782248684404969	0.5184621072362934
0.0432531599727103	0.5839691372618507	0.5276267298331224
0.4965491291270730	0.5980659261398447	0.5677935790927112
0.5476338319715088	0.5648384468854116	0.7223616257630642
0.4633298703280916	0.4749267029702476	0.5461290950844210
0.4663852809976675	0.9889720526249979	0.5272681125789287
0.9702914932987534	0.4864780322565554	0.5236017509994154
0.4763879873436023	0.7062785524734380	0.5795784666288560
0.2532556789533886	0.7890318590803594	0.5082582360712470
0.1732096451214162	0.4773557972208232	0.5202245657634047
0.4633490181426027	0.2757215200781346	0.4746426759838633
0.6821687375662409	0.1861664781347091	0.5419047903244961
0.7694791138617997	0.4996135243350093	0.5288432188188871
0.6786628831613202	0.4913606850049485	0.5603230807303049
0.7709640167496175	0.2716722557439849	0.5117853037754422
0.4601745301848139	0.1877847212402494	0.4850946399328676
0.2666877881976465	0.4843329618772009	0.5046742850167621
0.1658481227851862	0.7021505255424372	0.5328958380069171
0.4782365047410911	0.7929619481366569	0.5655064128440183
0.5716289953213430	0.6546643627649841	0.6358407901281380
0.4048842352080370	0.4954979263307280	0.6475735275017833
0.4416586841953555	0.4547488604723444	0.6894357625881599
0.5199896558494362	0.6152335020463142	0.6359613467883887

0.4499846181341523 0.4934480727387510 0.6560442720107477

12) Cu^{II}-HHTP NH_2CONH_2

NH_2CONH_2

1.00000000000000

21.8798999786000010 0.0000000000000000 0.0000000000000000

-10.9349554910000002 18.9466972881999993 0.0000000000000000

0.0000000000000000 0.0000000000000000 20.0552997588999986

C O Cu H N

37 13 3 16 2

Direct

0.2358273876218271 0.5912225172038709 0.5215091603835244
0.5061021854922162 0.3900130616221344 0.5159653366194673
0.3035952477199606 0.5283036078833535 0.5169597582790056
0.3689265286840120 0.6581200911314422 0.5288275120364592
0.5708196307829866 0.1285139611713783 0.5283082499361910
0.4318513514899481 0.7890451754792174 0.5357519435059687
0.3022205178823108 0.7244069891337201 0.5244504066503886
0.8320447158581962 0.4552654846492247 0.5276917915778501
0.1712663221913504 0.6566381525670578 0.5272439642534890
0.7028044911446507 0.3907965860472457 0.5265587094296164
0.4334871534842433 0.5933155649567690 0.5292189476482448
0.6349887646677141 0.4536758602773739 0.5336169830952819
0.5701844101382597 0.3245950559801248 0.5161265723597228
0.3668691792119661 0.8536796096499033 0.5233551559662279
0.5065927317698503 0.1934106181213689 0.5111332861527147
0.6359770963547284 0.2576633276103814 0.5241625667563572
0.1065673869806148 0.5263058977196307 0.5217037099751537
0.7669324128865583 0.3248479185929838 0.5216771383121444
0.5698008795751078 0.2578961942243794 0.5160443582510417
0.3668662050380632 0.5266981859478719 0.5213484629829538
0.5075623974938491 0.3266771209983809 0.5098002433424872
0.6364862364689501 0.3907201688959416 0.5259274579278008
0.1064793838525942 0.5928752513172355 0.5261856613257549
0.7674187442838656 0.4542304746819346 0.5284268853588086
0.7026480760496048 0.3240060102138345 0.5241758106278651
0.4330636164117047 0.8537655550329426 0.5329963703034514
0.6344060445202900 0.1933196950902293 0.5316207360413346
0.3687704303057162 0.7244457646591220 0.5302652857120915
0.5719134779282384 0.4554521784737373 0.5302127911025197
0.4320775436706409 0.6567813100455910 0.5324868120162073
0.3022625341718825 0.5913862322170697 0.5217661103161191
0.8318898061868628 0.3884907903718353 0.5234395704517030
0.1713375132375576 0.5276798893018597 0.5199197939494997
0.2357226923639653 0.6578523483592399 0.5241880495009378
0.5049963273266953 0.1285447897118274 0.5163275062575629
0.3033802981257221 0.7887646775190095 0.5198393823113486
0.4867838380198992 0.5429842918898907 0.6861121679358816
0.0453135129238467 0.4689962925025702 0.5204891998344867
0.3710826027276319 0.9148218906921013 0.5188149823006746
0.8934134811779267 0.5124021801758938 0.5295701310304926
0.4492028006598503 0.3942348170904814 0.5114311185281578

0.5661935329492206 0.0673441612674362 0.5352414686794071
0.4480007304397095 0.0672112392870933 0.5124987068475908
0.5672932238517200 0.5119474097405479 0.5394327372161232
0.8929839848279354 0.3924145770832531 0.5211759192303416
0.4899205391141197 0.9151464348210366 0.5374399732690973
0.3706311859205101 0.4695658744862585 0.5191000654650937
0.0452890443198532 0.5886983089341274 0.5292133447085681
0.4904305671635550 0.5892077377851596 0.5320208739429971
0.4462691065900820 0.5581970369589824 0.7143281164573730
0.4682570240300397 0.4901335995091445 0.5307285253207474
0.4687833821134826 0.9911828926364917 0.5261687444719887
0.9692670242930045 0.4905471711701347 0.5252452173040788
0.4827808036382946 0.7050469551980731 0.5376249690362744
0.2551540153033792 0.7909898953740890 0.5129389323899586
0.1690153028319032 0.4768199875465608 0.5182578006305193
0.4571570431612140 0.2789875235492221 0.5009169305931080
0.6823385040415546 0.1911641150950558 0.5409736909440804
0.7699313690386417 0.5052031416754169 0.5294023369090529
0.6830621652293231 0.5035801655865575 0.5430567469552511
0.7690432026210128 0.2762885899744769 0.5175088096889284
0.4560784155765111 0.1910956145750780 0.5044498690803441
0.2554730246410945 0.4777545593894444 0.5103492297634192
0.1688277763284650 0.7050103513146213 0.5313860502176059
0.4824973398427269 0.7914829141461096 0.5416004826494332
0.4123912096824867 0.4411732481753731 0.6707636808280182
0.4957646665595015 0.4563072842141537 0.6605202525717790
0.5744690732061677 0.6386075621368948 0.6918795072892974
0.5828958971255125 0.5809715949920796 0.6393266840453228
0.5582268411016492 0.5877091987348073 0.6794792268747905
0.4625982615754800 0.4762751584321749 0.6560809772724113

2. CO_2 reduction reaction

1) Cu^{III}-HHTP CO_2

CO_2
1.00000000000000
21.8798999786000010 0.0000000000000000 0.0000000000000000
-10.9349554910000002 18.9466972881999993 0.0000000000000000
0.0000000000000000 0.0000000000000000 20.0552997588999986
C O Cu H
37 15 3 13
Direct
0.2365305880582631 0.5909539042794045 0.5223071527944515
0.5028060995585913 0.3853064973572224 0.5291732822917410
0.3067985564965668 0.5331898023999544 0.4977026199610530
0.3682364154919519 0.6560637214067674 0.5390234507145039
0.5737074265099199 0.1266796541562012 0.5273756843588281
0.4326101115690426 0.7867793029842720 0.5440739444499563
0.3031302207238834 0.7234746728448611 0.5300328523309794
0.8325685722877857 0.4541879532608354 0.5287464869949169
0.1719675716863791 0.6559863799122698 0.5313140298316374
0.7032257387672618 0.3893609938747704 0.5263028796444096
0.4316761445460016 0.5905056787085263 0.5361597088004417

0.6350824362780312	0.4520365303353442	0.5227556251357015
0.5697560843718094	0.3215116103201843	0.5237533795658940
0.3691647948515593	0.8524164169513233	0.5265974155581411
0.5080162951275478	0.1908910443516432	0.5144901285115485
0.6374881646896939	0.2558421474312979	0.5256642713604107
0.1075356972989159	0.5253536090543367	0.5238354673612301
0.7680703956848020	0.3239005901841153	0.5232899499796984
0.5706803749698123	0.2553012123708160	0.5208733858840408
0.3703414622425661	0.5314810892535090	0.5024228024629953
0.5069153089439282	0.3218096367023708	0.5272446171985609
0.6368459112850925	0.3893375622224624	0.5245619687350199
0.1073198757467454	0.5920109093120061	0.5287843404645050
0.7677406409558784	0.4528491111228394	0.5291837416691431
0.7034996585221369	0.3225796958756930	0.5252244434652481
0.4348887683733788	0.8518977520983738	0.5381009821556785
0.6368508125087108	0.1916240793129457	0.5302436961334800
0.3688728954177043	0.7227144980746100	0.5396323172435962
0.5713360414391019	0.4541845277828938	0.5233084495752034
0.4285707413529466	0.6517927936714385	0.5522210322243513
0.3031702163483716	0.5918907899808966	0.5188570284869988
0.8327327851074398	0.3875245124178989	0.5246282381929798
0.1725349826010575	0.5269788102425744	0.5212127155432580
0.2363166796423699	0.6572198544699598	0.5282193016343534
0.5072852601724266	0.1262767495220866	0.5174882389475899
0.3051692354240202	0.7879566546415930	0.5232148495401988
0.5035599797087066	0.5354907048453735	0.7087610599758550
0.0463287945281066	0.4680836245808597	0.5228495447835778
0.3742862497053260	0.9136702358921855	0.5192751781143217
0.8937721504528131	0.5113567426386453	0.5310941901770649
0.4465099259362044	0.3860859666210228	0.5362986859132719
0.5695665274641458	0.0655203085360916	0.5322982540003989
0.4506044443079608	0.0650073225641572	0.5130932124095129
0.5700627527357822	0.5116428321292630	0.5222581531500590
0.8939495424531149	0.3916996922375711	0.5227441821260917
0.4923257888318994	0.9129822762284073	0.5403367135607804
0.3788508863541524	0.4811796011319129	0.4806154370745788
0.0461046315020911	0.5876730874772733	0.5315065990144762
0.4859262626577701	0.5833100719407384	0.5448280115692052
0.5122915987014534	0.4883257304441491	0.6943292698041343
0.4946840339198580	0.5825247113601042	0.7237699319821024
0.4887219705420742	0.4691489777155026	0.4110812452845982
0.4788885048075312	0.5028509681039035	0.4924089754308543
0.4720831886465070	0.9892708348211978	0.5258875289856715
0.9699681847418019	0.4896199283572269	0.5272474000782014
0.4762518887253839	0.6965456765496049	0.5722959833785264
0.2575553075426893	0.7906079630454715	0.5136872505123929
0.1713724473609288	0.4766028599180248	0.5198586102992011
0.4562708755266068	0.2730170567187951	0.5296466516279030
0.6852741354915757	0.1897444332350864	0.5366279739405535
0.7700311673763962	0.5036703635480598	0.5316117799405680
0.6834214226046931	0.5030963635925095	0.5210858974047313

0.7705699041372613 0.2755182502796324 0.5203371368111988
0.4573835815463462 0.1882502507637172 0.5076836860586599
0.2610002661206386 0.4871486185858706 0.4766481533328006
0.1694659991698565 0.7042200994221933 0.5366984548047599
0.4828244328471153 0.7881621699813107 0.5495717064135593
0.4425598415015071 0.4457314268458425 0.3893527078376298

2) Cu^{III}-HHTP COOH

COOH
1.00000000000000
21.8798999786000010 0.0000000000000000 0.0000000000000000
-10.9349554910000002 18.9466972881999993 0.0000000000000000
0.0000000000000000 0.0000000000000000 20.0552997588999986
C O Cu H
37 15 3 14
Direct
0.2366166403201392 0.5933526484766459 0.5310073774181666
0.5064428687280953 0.3845907605698108 0.5223229163372329
0.3042200030296446 0.5314010373516119 0.5440281924756867
0.3695370647449092 0.6606622398100418 0.5286422506333820
0.5736325901325162 0.1268662959466572 0.5333332058108083
0.4320463873190036 0.7903366627047590 0.5211137584183558
0.3022373152018045 0.7265170777209340 0.5304715915302669
0.8346431777033939 0.4538616540106215 0.5232665072522218
0.1713692546578527 0.6579741263680526 0.5302739037608212
0.7058876675294199 0.3886460440242096 0.5241574593893126
0.4362845421300939 0.5981704011253524 0.5373906791133989
0.6384180427083066 0.4512047839310536 0.5310859686618182
0.5732297181461428 0.3217560640739797 0.5168529280849326
0.3674077660867980 0.8555390817579921 0.5319246164880312
0.5114381166367434 0.1920698758951419 0.5068443204346126
0.6397046301972183 0.2556047421854833 0.5274835073467086
0.1078957441094003 0.5274620130462108 0.5260819211966880
0.7704783176739067 0.3234193247742888 0.5295247946730772
0.5739477657539941 0.2557241399780273 0.5158758346482959
0.3679083611599112 0.5293231437883091 0.5440035623466571
0.5103301011411415 0.3219946334222753 0.5142834751632850
0.6398660359534256 0.3888121697137533 0.5243711686181485
0.1070432555095767 0.5937977423331250 0.5287367357768067
0.7699640101040469 0.4523163441721634 0.5211660004265868
0.7060518187337473 0.3221466592044525 0.5276801274610347
0.4332134161404835 0.8549040625479681 0.5203307026576115
0.6376295920404658 0.1913748976955760 0.5370775385586438
0.3687862348178591 0.7265221812113899 0.5259675755905197
0.5749879029655941 0.4534713550468875 0.5287578094243076
0.4325671146028571 0.6604014037397964 0.5275115363586059
0.3027517318480793 0.5935051115070917 0.5350646910464401
0.8350595046711011 0.3873494789883846 0.5278414496662550
0.1728293790207395 0.5294422423270965 0.5272999628476410
0.2360608939171188 0.6597385527469872 0.5308978570399617
0.5098781480008580 0.1273653292458473 0.5117055204583717
0.3034273216900397 0.7908585206575789 0.5337919841254415

0.4785093807832420 0.4826316119406652 0.6335595870798685
0.0472479008953956 0.4697325964950634 0.5226333271000362
0.3730727996148245 0.9166746007888981 0.5402829930701104
0.8955292773286452 0.5113746852834132 0.5208612276758561
0.4492290181908951 0.3846132661919376 0.5242356490668423
0.5662227840325211 0.0660174696929539 0.5478007801246116
0.4549650055347426 0.0671308671265852 0.4986492889803603
0.5734587175840531 0.5105155534577638 0.5297571293187500
0.8963058030671364 0.3917486225818089 0.5293126873365688
0.4894354938933251 0.9149438643903919 0.5097822380774449
0.3698929668160054 0.4729128398053501 0.5496950237076998
0.0456324062432706 0.5890675696809473 0.5288173028001583
0.4936648235332207 0.5985406252058536 0.5433081517162668
0.5007430215381083 0.4472249893247585 0.6588200250456271
0.4574537100836846 0.5215587775661992 0.6690978393255065
0.4641596492385332 0.4979922503916763 0.4447915286514275
0.4715679960249886 0.4916630228271091 0.5370871696265171
0.4707793180328914 0.9911964635765144 0.5247295449741913
0.9711800613927377 0.4903354070253479 0.5251033018862996
0.4831790480753917 0.7086153095458826 0.5219992078103409
0.2553044345353981 0.7934892817446385 0.5395516503015426
0.1714344388452807 0.4790438236798424 0.5246016227001560
0.4597142246647865 0.2736519844404866 0.5092900359137814
0.6848452170176691 0.1889794807665103 0.5495483933906889
0.7718617473084960 0.5028113020072162 0.5162913788888496
0.6864827310190503 0.5016171948037513 0.5385653532099151
0.7731072689257044 0.2750269818226606 0.5312932689226975
0.4617505069504345 0.1898276866830481 0.4952433022032497
0.2560947840201752 0.4809273063945635 0.5509453938185456
0.1682175795793417 0.7061033786244382 0.5303440860311377
0.4827792306171106 0.7927547035208851 0.5167212907922044
0.4642212116277786 0.5148411834179225 0.7165751960821274
0.5107248908587194 0.5335621900953424 0.4288055611304766

3) Cu^{III}-HHTP CO

CO
1.00000000000000
21.8798999786000010 0.0000000000000000 0.0000000000000000
-10.9349554910000002 18.9466972881999993 0.0000000000000000
0.0000000000000000 0.0000000000000000 20.0552997588999986
C O Cu H
37 14 3 13
Direct
0.2355972478624329 0.5936154152693508 0.5274923287564777
0.5079875848940324 0.3817135091283671 0.5356791449159092
0.3027076939681900 0.5310624754100584 0.5345747498583163
0.3675700581461379 0.6607961968420074 0.5436016892810872
0.5748241815740534 0.1256092766660476 0.5189231980451382
0.4306214096036158 0.7908194817446170 0.5432888651587305
0.3017996879604839 0.7264149354844353 0.5255177487314568
0.8351387157029960 0.4530958766706230 0.5111304410439294
0.1709720337186492 0.6585741144170422 0.5208136923133744

0.7068628926953429	0.3870254160195551	0.5155408006816292
0.4321171084044152	0.5967634185743629	0.5606637799836343
0.6403217726005378	0.4482455150869420	0.5368227555110539
0.5741387008532904	0.3200114390491921	0.5148972304485310
0.3675020974338484	0.8551107645747984	0.5187037241555564
0.5121450544233381	0.1911134177024038	0.4979297720193563
0.6408735370529700	0.2543216876945447	0.5164900667424477
0.1072543343746101	0.5280389678181744	0.5209796012912722
0.7719055940686701	0.3224537933457368	0.5168751890526476
0.5750133247753434	0.2546850011592769	0.5076940726541354
0.3644029164971900	0.5279247745316868	0.5491126200549522
0.5111691790589274	0.3202383772588463	0.5194543164381512
0.6410485297493728	0.3866874435058426	0.5222129068981360
0.1065662289047234	0.5944147280445277	0.5190716522441943
0.7703137905912620	0.4510203884908703	0.5106224843436059
0.7073575772083995	0.3207909552053193	0.5166958420801044
0.4326721177886867	0.8550981562785912	0.5343496001143127
0.6388978471522685	0.1899168884083973	0.5232306917401921
0.3674118757724639	0.7265815678764556	0.5381294637733218
0.5770157689645024	0.4498591558441635	0.5455928966550140
0.4294889748261341	0.6599377162156264	0.5557635824204856
0.3013737403805667	0.5936524975405445	0.5344422435596003
0.8361382902304780	0.3869016016630673	0.5156324775164604
0.1717689807835047	0.5297511618251536	0.5255754198114443
0.2353244904279377	0.6599137015146789	0.5243293577860317
0.5098951469480142	0.1263407343856467	0.5035524372809067
0.3037174928332562	0.7904371585251556	0.5153042799916648
0.4601109395904624	0.4739759488751881	0.6520812568248356
0.0466426647016270	0.4700483258698686	0.5193145265558358
0.3726911710155306	0.9160214186782172	0.5087284710169322
0.8956898512012437	0.5110561120001864	0.5082937887258547
0.4506993581987555	0.3808341746487046	0.5448004028118966
0.5691970115249367	0.0643524542707765	0.5275519575039046
0.4524280762627686	0.0656578859404569	0.4981158791188152
0.5762555511591707	0.5049958776000529	0.5629807194763661
0.8975651267983444	0.3918089899645011	0.5175924442897109
0.4901618853373532	0.9160187291108942	0.5380637266850152
0.3641664792489341	0.4704508229371794	0.5545102286767631
0.0452754493597183	0.5898617668299917	0.5163957463937927
0.4879301300331227	0.5959384261858662	0.5746775678970519
0.4555956078396529	0.4688907945332358	0.7089362295345901
0.4799965268221006	0.5045452295258068	0.4684570156732306
0.4707185073123760	0.4887882420127047	0.5592313565249354
0.4708907779002597	0.9904463862105931	0.5177755136590909
0.9713364099679492	0.4908489241540230	0.5148477249208523
0.4795989723652757	0.7082699815220125	0.5632638738394982
0.2562901882347979	0.7927399581085852	0.5036284276413889
0.1696171501213478	0.4790521257462759	0.5287635915112787
0.4603428615744355	0.2719937358788282	0.5151938663498807
0.6863854591173485	0.1870067113151685	0.5328117471204972
0.7713484682104863	0.5011750056456428	0.5066345007363825

0.6889171223131699 0.4977794025556534 0.5457584795822994
0.7750933261392376 0.2743384003077939 0.5175365198658172
0.4624439281527015 0.1896066010569531 0.4882564947325500
0.2554711627135796 0.4803373759912601 0.5258203237117778
0.1680760674236041 0.7068541646382497 0.5202229768123720
0.4807221191592471 0.7933526317172416 0.5533127394095495
0.5246254559698019 0.5483057833959740 0.4614308990439304

4) Cu^{II}-HHTP CO_2

CO_2
1.00000000000000
21.8798999786000010 0.0000000000000000 0.0000000000000000
-10.9349554910000002 18.9466972881999993 0.0000000000000000
0.0000000000000000 0.0000000000000000 20.0552997588999986
C O Cu H
37 15 3 13
Direct
0.2365305880582631 0.5909539042794045 0.5223071527944515
0.5028060995585913 0.3853064973572224 0.5291732822917410
0.3067985564965668 0.5331898023999544 0.4977026199610530
0.3682364154919519 0.6560637214067674 0.5390234507145039
0.5737074265099199 0.1266796541562012 0.5273756843588281
0.4326101115690426 0.7867793029842720 0.5440739444499563
0.3031302207238834 0.7234746728448611 0.5300328523309794
0.8325685722877857 0.4541879532608354 0.5287464869949169
0.1719675716863791 0.6559863799122698 0.5313140298316374
0.7032257387672618 0.3893609938747704 0.5263028796444096
0.4316761445460016 0.5905056787085263 0.5361597088004417
0.6350824362780312 0.4520365303353442 0.5227556251357015
0.5697560843718094 0.3215116103201843 0.5237533795658940
0.3691647948515593 0.8524164169513233 0.5265974155581411
0.5080162951275478 0.1908910443516432 0.5144901285115485
0.6374881646896939 0.2558421474312979 0.5256642713604107
0.1075356972989159 0.5253536090543367 0.5238354673612301
0.7680703956848020 0.3239005901841153 0.5232899499796984
0.5706803749698123 0.2553012123708160 0.5208733858840408
0.3703414622425661 0.5314810892535090 0.5024228024629953
0.5069153089439282 0.3218096367023708 0.5272446171985609
0.6368459112850925 0.3893375622224624 0.5245619687350199
0.1073198757467454 0.5920109093120061 0.5287843404645050
0.7677406409558784 0.4528491111228394 0.5291837416691431
0.7034996585221369 0.3225796958756930 0.5252244434652481
0.4348887683733788 0.8518977520983738 0.5381009821556785
0.6368508125087108 0.1916240793129457 0.5302436961334800
0.3688728954177043 0.7227144980746100 0.5396323172435962
0.5713360414391019 0.4541845277828938 0.5233084495752034
0.4285707413529466 0.6517927936714385 0.5522210322243513
0.3031702163483716 0.5918907899808966 0.5188570284869988
0.8327327851074398 0.3875245124178989 0.5246282381929798
0.1725349826010575 0.5269788102425744 0.5212127155432580
0.2363166796423699 0.6572198544699598 0.5282193016343534
0.5072852601724266 0.1262767495220866 0.5174882389475899

0.3051692354240202 0.7879566546415930 0.5232148495401988
0.5035599797087066 0.5354907048453735 0.7087610599758550
0.0463287945281066 0.4680836245808597 0.5228495447835778
0.3742862497053260 0.9136702358921855 0.5192751781143217
0.8937721504528131 0.5113567426386453 0.5310941901770649
0.4465099259362044 0.3860859666210228 0.5362986859132719
0.5695665274641458 0.0655203085360916 0.5322982540003989
0.4506044443079608 0.0650073225641572 0.5130932124095129
0.5700627527357822 0.5116428321292630 0.5222581531500590
0.8939495424531149 0.3916996922375711 0.5227441821260917
0.4923257888318994 0.9129822762284073 0.5403367135607804
0.3788508863541524 0.4811796011319129 0.4806154370745788
0.0461046315020911 0.5876730874772733 0.5315065990144762
0.4859262626577701 0.5833100719407384 0.5448280115692052
0.5122915987014534 0.4883257304441491 0.6943292698041343
0.4946840339198580 0.5825247113601042 0.7237699319821024
0.4887219705420742 0.4691489777155026 0.4110812452845982
0.4788885048075312 0.5028509681039035 0.4924089754308543
0.4720831886465070 0.9892708348211978 0.5258875289856715
0.9699681847418019 0.4896199283572269 0.5272474000782014
0.4762518887253839 0.6965456765496049 0.5722959833785264
0.2575553075426893 0.7906079630454715 0.5136872505123929
0.1713724473609288 0.4766028599180248 0.5198586102992011
0.4562708755266068 0.2730170567187951 0.5296466516279030
0.6852741354915757 0.1897444332350864 0.5366279739405535
0.7700311673763962 0.5036703635480598 0.5316117799405680
0.6834214226046931 0.5030963635925095 0.5210858974047313
0.7705699041372613 0.2755182502796324 0.5203371368111988
0.4573835815463462 0.1882502507637172 0.5076836860586599
0.2610002661206386 0.4871486185858706 0.4766481533328006
0.1694659991698565 0.7042200994221933 0.5366984548047599
0.4828244328471153 0.7881621699813107 0.5495717064135593
0.4425598415015071 0.4457314268458425 0.3893527078376298

5) Cu^{II}-HHTP COOH

COOH
1.00000000000000
21.8798999786000010 0.0000000000000000 0.0000000000000000
-10.9349554910000002 18.9466972881999993 0.0000000000000000
0.0000000000000000 0.0000000000000000 20.0552997588999986
C O Cu H
37 15 3 14
Direct
0.2366166403201392 0.5933526484766459 0.5310073774181666
0.5064428687280953 0.3845907605698108 0.5223229163372329
0.3042200030296446 0.5314010373516119 0.5440281924756867
0.3695370647449092 0.6606622398100418 0.5286422506333820
0.5736325901325162 0.1268662959466572 0.5333332058108083
0.4320463873190036 0.7903366627047590 0.5211137584183558
0.3022373152018045 0.7265170777209340 0.5304715915302669
0.8346431777033939 0.4538616540106215 0.5232665072522218
0.1713692546578527 0.6579741263680526 0.5302739037608212

0.7058876675294199	0.3886460440242096	0.5241574593893126
0.4362845421300939	0.5981704011253524	0.5373906791133989
0.6384180427083066	0.4512047839310536	0.5310859686618182
0.5732297181461428	0.3217560640739797	0.5168529280849326
0.3674077660867980	0.8555390817579921	0.5319246164880312
0.5114381166367434	0.1920698758951419	0.5068443204346126
0.6397046301972183	0.2556047421854833	0.5274835073467086
0.1078957441094003	0.5274620130462108	0.5260819211966880
0.7704783176739067	0.3234193247742888	0.5295247946730772
0.5739477657539941	0.2557241399780273	0.5158758346482959
0.3679083611599112	0.5293231437883091	0.5440035623466571
0.5103301011411415	0.3219946334222753	0.5142834751632850
0.6398660359534256	0.3888121697137533	0.5243711686181485
0.1070432555095767	0.5937977423331250	0.5287367357768067
0.7699640101040469	0.4523163441721634	0.5211660004265868
0.7060518187337473	0.3221466592044525	0.5276801274610347
0.4332134161404835	0.8549040625479681	0.5203307026576115
0.6376295920404658	0.1913748976955760	0.5370775385586438
0.3687862348178591	0.7265221812113899	0.5259675755905197
0.5749879029655941	0.4534713550468875	0.5287578094243076
0.4325671146028571	0.6604014037397964	0.5275115363586059
0.3027517318480793	0.5935051115070917	0.5350646910464401
0.8350595046711011	0.3873494789883846	0.5278414496662550
0.1728293790207395	0.5294422423270965	0.5272999628476410
0.2360608939171188	0.6597385527469872	0.5308978570399617
0.5098781480008580	0.1273653292458473	0.5117055204583717
0.3034273216900397	0.7908585206575789	0.5337919841254415
0.4785093807832420	0.4826316119406652	0.6335595870798685
0.0472479008953956	0.4697325964950634	0.5226333271000362
0.3730727996148245	0.9166746007888981	0.5402829930701104
0.8955292773286452	0.5113746852834132	0.5208612276758561
0.4492290181908951	0.3846132661919376	0.5242356490668423
0.5662227840325211	0.0660174696929539	0.5478007801246116
0.4549650055347426	0.0671308671265852	0.4986492889803603
0.5734587175840531	0.5105155534577638	0.5297571293187500
0.8963058030671364	0.3917486225818089	0.5293126873365688
0.4894354938933251	0.9149438643903919	0.5097822380774449
0.3698929668160054	0.4729128398053501	0.5496950237076998
0.0456324062432706	0.5890675696809473	0.5288173028001583
0.4936648235332207	0.5985406252058536	0.5433081517162668
0.5007430215381083	0.4472249893247585	0.6588200250456271
0.4574537100836846	0.5215587775661992	0.6690978393255065
0.4641596492385332	0.4979922503916763	0.4447915286514275
0.4715679960249886	0.4916630228271091	0.5370871696265171
0.4707793180328914	0.9911964635765144	0.5247295449741913
0.9711800613927377	0.4903354070253479	0.5251033018862996
0.4831790480753917	0.7086153095458826	0.5219992078103409
0.2553044345353981	0.7934892817446385	0.5395516503015426
0.1714344388452807	0.4790438236798424	0.5246016227001560
0.4597142246647865	0.2736519844404866	0.5092900359137814
0.6848452170176691	0.1889794807665103	0.5495483933906889

0.7718617473084960 0.5028113020072162 0.5162913788888496
0.6864827310190503 0.5016171948037513 0.5385653532099151
0.7731072689257044 0.2750269818226606 0.5312932689226975
0.4617505069504345 0.1898276866830481 0.4952433022032497
0.2560947840201752 0.4809273063945635 0.5509453938185456
0.1682175795793417 0.7061033786244382 0.5303440860311377
0.4827792306171106 0.7927547035208851 0.5167212907922044
0.4642212116277786 0.5148411834179225 0.7165751960821274
0.5107248908587194 0.5335621900953424 0.4288055611304766

6) Cu^{II}-HHTP CO

CO
1.00000000000000
21.8798999786000010 0.0000000000000000 0.0000000000000000
-10.9349554910000002 18.9466972881999993 0.0000000000000000
0.0000000000000000 0.0000000000000000 20.0552997588999986
C O Cu H
37 14 3 13
Direct
0.2355972478624329 0.5936154152693508 0.5274923287564777
0.5079875848940324 0.3817135091283671 0.5356791449159092
0.3027076939681900 0.5310624754100584 0.5345747498583163
0.3675700581461379 0.6607961968420074 0.5436016892810872
0.5748241815740534 0.1256092766660476 0.5189231980451382
0.4306214096036158 0.7908194817446170 0.5432888651587305
0.3017996879604839 0.7264149354844353 0.5255177487314568
0.8351387157029960 0.4530958766706230 0.5111304410439294
0.1709720337186492 0.6585741144170422 0.5208136923133744
0.7068628926953429 0.3870254160195551 0.5155408006816292
0.4321171084044152 0.5967634185743629 0.5606637799836343
0.6403217726005378 0.4482455150869420 0.5368227555110539
0.5741387008532904 0.3200114390491921 0.5148972304485310
0.3675020974338484 0.8551107645747984 0.5187037241555564
0.5121450544233381 0.1911134177024038 0.4979297720193563
0.6408735370529700 0.2543216876945447 0.5164900667424477
0.1072543343746101 0.5280389678181744 0.5209796012912722
0.7719055940686701 0.3224537933457368 0.5168751890526476
0.5750133247753434 0.2546850011592769 0.5076940726541354
0.3644029164971900 0.5279247745316868 0.5491126200549522
0.5111691790589274 0.3202383772588463 0.5194543164381512
0.6410485297493728 0.3866874435058426 0.5222129068981360
0.1065662289047234 0.5944147280445277 0.5190716522441943
0.7703137905912620 0.4510203884908703 0.5106224843436059
0.7073575772083995 0.3207909552053193 0.5166958420801044
0.4326721177886867 0.8550981562785912 0.5343496001143127
0.6388978471522685 0.1899168884083973 0.5232306917401921
0.3674118757724639 0.7265815678764556 0.5381294637733218
0.5770157689645024 0.4498591558441635 0.5455928966550140
0.4294889748261341 0.6599377162156264 0.5557635824204856
0.3013737403805667 0.5936524975405445 0.5344422435596003
0.8361382902304780 0.3869016016630673 0.5156324775164604
0.1717689807835047 0.5297511618251536 0.5255754198114443

0.2353244904279377	0.6599137015146789	0.5243293577860317
0.5098951469480142	0.1263407343856467	0.5035524372809067
0.3037174928332562	0.7904371585251556	0.5153042799916648
0.4601109395904624	0.4739759488751881	0.6520812568248356
0.0466426647016270	0.4700483258698686	0.5193145265558358
0.3726911710155306	0.9160214186782172	0.5087284710169322
0.8956898512012437	0.5110561120001864	0.5082937887258547
0.4506993581987555	0.3808341746487046	0.5448004028118966
0.5691970115249367	0.0643524542707765	0.5275519575039046
0.4524280762627686	0.0656578859404569	0.4981158791188152
0.5762555511591707	0.5049958776000529	0.5629807194763661
0.8975651267983444	0.3918089899645011	0.5175924442897109
0.4901618853373532	0.9160187291108942	0.5380637266850152
0.3641664792489341	0.4704508229371794	0.5545102286767631
0.0452754493597183	0.5898617668299917	0.5163957463937927
0.4879301300331227	0.5959384261858662	0.5746775678970519
0.4555956078396529	0.4688907945332358	0.7089362295345901
0.4799965268221006	0.5045452295258068	0.4684570156732306
0.4707185073123760	0.4887882420127047	0.5592313565249354
0.4708907779002597	0.9904463862105931	0.5177755136590909
0.9713364099679492	0.4908489241540230	0.5148477249208523
0.4795989723652757	0.7082699815220125	0.5632638738394982
0.2562901882347979	0.7927399581085852	0.5036284276413889
0.1696171501213478	0.4790521257462759	0.5287635915112787
0.4603428615744355	0.2719937358788282	0.5151938663498807
0.6863854591173485	0.1870067113151685	0.5328117471204972
0.7713484682104863	0.5011750056456428	0.5066345007363825
0.6889171223131699	0.4977794025556534	0.5457584795822994
0.7750933261392376	0.2743384003077939	0.5175365198658172
0.4624439281527015	0.1896066010569531	0.4882564947325500
0.2554711627135796	0.4803373759912601	0.5258203237117778
0.1680760674236041	0.7068541646382497	0.5202229768123720
0.4807221191592471	0.7933526317172416	0.5533127394095495
0.5246254559698019	0.5483057833959740	0.4614308990439304

Supplementary References

[S1] M. Yuan, J. Chen, Y. Bai, Z. Liu, J. Zhang, et al., Unveiling electrochemical urea synthesis by co-activation of CO_2 and N_2 with mott-schottky heterostructure catalysts. Angew. Chem. Int. Ed. **60**(19), 10910-10918 (2021). https://doi.org/10.1002/anie.202101275

[S2] C. Chen, X. Zhu, X. Wen, Y. Zhou, L. Zhou, et al., Coupling N_2 and CO_2 in H_2O to synthesize urea under ambient conditions. Nat. Chem. **12**(8), 717-724 (2020). https://doi.org/10.1038/s41557-020-0481-9

[S3] M. Yuan, J. Chen, Y. Bai, Z. Liu, J. Zhang, et al., Electrochemical C-N coupling with perovskite hybrids toward efficient urea synthesis. Chem. Sci. **12**(17), 6048-6058 (2021). https://doi.org/10.1039/d1sc01467f

[S4] M. Yuan, J. Chen, Y. Xu, R. Liu, T. Zhao, et al., Highly selective electroreduction of N_2 and CO_2 to urea over artificial frustrated lewis pairs. Energy Environ Sci. **14**(12), 6605-6615 (2021). https://doi.org/10.1039/d1ee02485j

[S5] M. Yuan, H. Zhang, Y. Xu, R. Liu, R. Wang, et al., Artificial frustrated lewis pairs facilitating the electrochemical N_2 and CO_2 conversion to urea. Chem. Catal. **2**(2), 309-320 (2022). https://doi.org/10.1016/j.checat.2021.11.009

[S6] N. Meng, Y. Huang, Y. Liu, Y. Yu, B. Zhang. Electrosynthesis of urea from nitrite and CO_2 over oxygen vacancy-rich ZnO porous nanosheets. Cell. Rep. Phys. Sci. **2**(3), 100378 (2021). https://doi.org/10.1016/j.xcrp.2021.100378

[S7] N. Cao, Y. Quan, A. Guan, C. Yang, Y. Ji, et al., Oxygen vacancies enhanced cooperative electrocatalytic reduction of carbon dioxide and nitrite ions to urea. J Colloid. Interface. Sci. **577**, 109-114 (2020). https://doi.org/10.1016/j.jcis.2020.05.014

[S8] M. Yuan, J. Chen, H. Zhang, Q. Li, L. Zhou, et al., Host–guest molecular interaction promoted urea electrosynthesis over a precisely designed conductive metal–organic framework. Energy Environ Sci. (2022). https://doi.org/10.1039/d1ee03918k
